# Supplementary material for: Global Profiling of the Antioxidant Constituents in Chebulae Fructus Based on an Integrative Strategy of UHPLC/IM-QTOF-MS, MS/MS Molecular Networking, and Spectrum-Effect Correlation
Source: Antioxidants (Basel). 2023 Dec 8;12(12):2093. doi: 10.3390/antiox12122093 (PMC10741031; doi:10.3390/antiox12122093)
Supplement: Supplementary file 1 [file antioxidants-12-02093-s001.zip › antioxidants-2738643-supplementary.pdf]

Global Profiling of the Antioxidant Constituents in Chebulae Fructus Based on an Integrative Strategy of UHPLC/IM-QTOF-MS, MS/MS Molecular Networking, and Spectrum-Effect Correlation

Xiangdong Wang [1,†], Jian Xu [1,†], Li-Hua Zhang [1], Wenzhi Yang [1], Huijuan Yu [1,2], Min Zhang [1,2,*], Yuefei Wang [1,2,*] and Hong-Hua Wu [1,2,*]

[1] State Key Laboratory of Component-Based Chinese Medicine, National Key Laboratory of Chinese Medicine Modernization, Institute of Traditional Chinese Medicine, Tianjin University of Traditional Chinese Medicine, 10 Poyanghu Road, West Area, Tuanbo New Town, Jinghai District, Tianjin 301617, China

[2] Haihe Laboratory of Modern Chinese Medicine, 10 Poyanghu Road, West Area, Tuanbo New Town, Jinghai District, Tianjin 301617, China

* Correspondence: zhangm036@tjutcm.edu.cn (M.Z.), wangyf0622@tjutcm.edu.cn (Y.W.); wuhonghua2011@tjutcm.edu.cn (H.-H.W.).; Tel.: +86-22-5979-1833 (M.Z., Y.W. & H.-H.W.)

† These authors contributed equally to this work.

Table of contents .... Pages

S1. UHPLC-QTOF-MS analysis 3

S2. UPLC/IM-QTOF-MS analysis 1

S3. Evaluation of antioxidant capacity 2

Table S1. Information of the 18 batches of CF. 4

Table S2. Composition of the mixed standard solution. 5

Table S3. The currently reported constituents collated in our in-house compound library of CF. 6

Table S4. Identification of constituents by in-house compound library search. 12

Table S5. Calculated similarities for 18 batches of CF. 15

Table S6. VIP values of constituents calculated from OPLS-DA. 16

Table S7. The antioxidant capacities of the eighteen batches of CF. 17

Table S8. Stability evaluation and the result of UPLC-DPPH$^{\bullet}$ analysis. 18

Fig. S1 ^{1}H NMR (600 MHz, CD_3OD) spectrum of **chebulic acid** 19

Fig. S2 ^{13}C NMR (150 MHz, CD_3OD) spectrum of **chebulic acid** 19

Fig. S3 ^{1}H NMR (600 MHz, CD_3OD) spectrum of **gallic acid** 20

Fig. S4 ^{13}C NMR (150 MHz, CD_3OD) spectrum of **gallic acid** 20

Fig. S5 ^{1}H NMR (600 MHz, CD_3OD) spectrum of **4-galloyl-(-)-shikimic acid** 21

Fig. S6 ^{13}C NMR (150 MHz, CD_3OD) spectrum of **4-galloyl-(-)-shikimic acid** 21

Fig. S7 ^{1}H NMR (600 MHz, DMSO-d_6) spectrum of **5-galloyl-(-)-shikimic acid** 22

Fig. S8 ^{13}C NMR (150 MHz, DMSO-d_6) spectrum of **5-galloyl-(-)-shikimic acid** ... 22

Fig. S9 ^{1}H NMR (600 MHz, CD_3OD) spectrum of **corilagin** 23

Fig. S10 ^{13}C NMR (150 MHz, CD_3OD) spectrum of **corilagin** 23

Fig. S11 ^{1}H NMR (600 MHz, CD_3OD) spectrum of **1,3,6-tri-*O*-*β*-D-galloylglucose** 24

Fig. S12 ^{13}C NMR (150 MHz, CD_3OD) spectrum of **1,3,6-tri-*O*-*β*-D-galloylglucose** 24

Fig. S13 ^{1}H NMR (600 MHz, DMSO-d_6) spectrum of **chebulanin** 25

Fig. S14 ^{13}C NMR (150 MHz, DMSO-d_6) spectrum of **chebulanin** 25

Fig. S15 HSQC spectrum of **chebulanin** 26

Fig. S16 HMBC spectrum of **chebulanin** 26
Fig. S17 ^{1}H-^{1}H COSY spectrum of **chebulanin** 27
Fig. S18 ^{1}H NMR (600 MHz, CD_3OD) spectrum of **urolithin M5**...........................27
Fig. S19 ^{13}C NMR (150 MHz, CD_3OD) spectrum of **urolithin M5** 29
Fig. S20 ^{1}H NMR (600 MHz, CD_3OD) spectrum of **chebulagic acid** 29
Fig. S21 ^{13}C NMR (150 MHz, CD_3OD) spectrum of **chebulagic acid** 29
Fig. S22 ^{1}H NMR (600 MHz, CD_3OD) spectrum of **chebulinic acid**........................29
Fig. S23 ^{13}C NMR (150 MHz, CD_3OD) spectrum of **chebulinic acid**.......................30
Fig. S24 ^{1}H NMR (600 MHz, CD_3OD) spectrum of **ellagic acid** 30
Fig. S25 ^{13}C NMR (150 MHz, CD_3OD) spectrum of **ellagic acid** 31
Fig. S26 ^{1}H NMR (600 MHz, DMSO-d_6) spectrum of **terminalin**...........................31
Fig. S27 ^{13}C NMR (150 MHz, DMSO-d_6) spectrum of **terminalin**..........................32
Fig. S28 ^{1}H NMR (600 MHz, CD_3OD) spectrum of **4-*O*-(3'',4''-di-*O*-galloyl-*α*-L-rhamnosyl)ellagic acid** 32
Fig. S29 ^{13}C NMR (150 MHz, CD_3OD) spectrum of **4-*O*-(3'',4''-di-*O*-galloyl-*α*-L-rhamnosyl)ellagic acid** 33
Fig. S30 ^{1}H NMR (600 MHz, DMSO-d_6) spectrum of **1-*O*-galloyl-*β*-D-glucose**......33
Fig. S31 ^{13}C NMR (150 MHz, DMSO-d_6) spectrum of **1-*O*-galloyl-*β*-D-glucose**.....34
Fig. S32 ^{1}H NMR (600 MHz, CD_3OD) spectrum of **eschweilenol C** 34
Fig. S33 ^{13}C NMR (150 MHz, CD_3OD) spectrum of **eschweilenol C** 35
Fig. S34 Chemical structures of the identified 122 compounds. 36
Fig. S35 Annotation on nodes of precursor ion in component **6**. 41
Fig. S36 Annotation on nodes of precursor ion in component **17**. 42
Fig. S37 Annotation on nodes of precursor ion in component **13**. 43
Fig. S38 Annotation on nodes of precursor ion in component **14**. 44
Fig. S39 Annotation on nodes of precursor ion in component **4**. 45
Fig. S40 The cleavage law of **chebulic acid** in mass spectrometry 46
Fig. S41 The cleavage law of **gallic acid**, **4-*O*-galloyl-(-)-shikimic acid**, and **5-*O*-galloyl-(-)-shikimic acid** in mass spectrometry 46
Fig. S42 The cleavage law of **punicalagins A & B** in mass spectrometry 47

Fig. S43 The cleavage law of **corilagin** and **ellagic acid** in mass spectrometry.........47
Fig. S44 The cleavage law of **1,3,6-tri-*O*-galloyl-*β*-D-glucose** in mass spectrometry 49
Fig. S45 The cleavage law of **chebulanin** in mass spectrometry...............................49
Fig. S46 The cleavage law of **chebulagic acid** in mass spectrometry 49
Fig. S47 The cleavage law of **chebulinic acid** in mass spectrometry.........................49
Fig. S48 The cleavage law of **terminalin** in mass spectrometry 50
Fig. S49 The cleavage law of **4-*O*-(3'',4''-di-*O*-galloyl-*α*-L-rhamnosyl)ellagic acid** in mass spectrometry..50
Fig. S50 UPLC fingerprints of 18 batches of CF (Figure 4A). 51
Fig. S51 The representative UPLC fingerprints marked with 23 common peaks (Figure 4B)..52
Fig. S52 The heat-map of 18 batches of CF and the twenty-three constituents (Figure 4C)..53
Fig. S53 DPPH• radical scavenging activities of 18 batches of the methanol extracts from CF. 54

S1. UHPLC-QTOF-MS analysis

The accurately weighed CF crude extract (150.0 mg) was ultrasonically dissolved in 10.0 mL 50% aqueous methanol, the fraction F1 (150.0 mg) was ultrasonically dissolved in 10.0 mL water, and the fraction F2 (150.0 mg) was ultrasonically dissolved in 1.0 mL methanol to afford the tested solutions, which were then filtered through 0.22 μm microporous membrane filters (YM-0309, Tianjin Bo Jin technology Co., Ltd., Tianjin, China) and transferred into sealed vials and stored at 4 °C before analysis.

The fourteen standard substances were accurately weighed and dissolved in methanol, and then mixed to obtain the mixture solution. The mixed standard solution was then centrifuged at 17,709× g for 10 min, and the supernatant was then transferred into sealed vials and stored at 4 °C before analysis.

UHPLC-QTOF-MS Analysis was conducted on an UHPLC system (Agilent 1260 Infinity II) coupled with an Agilent 6550 QTOF™ high-resolution mass spectrometer (Agilent, CA, USA). Chromatographic separation was achieved on a COSMOSIL PBr column (2.1 mm × 100 mm, 2.6 μm) at 30 °C. The mobile phase composed of 0.1% formic acid in water (A) and methanol (B) ran at a flow rate of 0.3 mL/min. The injection volume was 5 μL and pre-equilibrated for 10 min. The elution program as the following: 0–5 min, 0%–5% B; 5–9 min, 5%–30% B; 9–12 min, 30%–33% B; 12–19 min, 33%–50% B; 19–28 min, 50%–81% B; 28–30 min, 81%–100% B; 30–33 min, 100% B, 33–36 min, 0% B; 36–40 min, 0% B. All samples were injected once in negative ion mode.

The ion source parameters of the high-resolution QTOF-MS were set as follows: Gas temperature 200 °C; Drying gas, 12 L/min; Nebulizing pressure, 40 psi; Sheath gas temperature, 350 °C; Sheath gas flow, 11 L/min; Nozzle voltage, 1.0 kV; Capillary voltage, 3.5 kV; Fragmentor, 390 V; Collision energy (CE), 30 eV. The TOF analyzer scanned over the mass-to-charge ratio (*m/z*) range of 100–1500 for MS^1 and 50–1500 for MS^2. The acquisition rates for MS^1 and MS^2 were 3 spectra/s and 4 spectra/s, respectively. Isolation width was set at the medium level (~4 *m/z*). The precursor ions giving Top 3 intensity in the MS^1 spectra were automatedly selected to trigger the MS/MS fragmentation by CID with threshold 100 counts.

S2. UPLC/IM-QTOF-MS analysis

The accurately weighed CF crude extract (150.0 mg) was ultrasonically dissolved in 10.0 mL 50% aqueous methanol, the fraction F1 (150.0 mg) was ultrasonically dissolved in 10.0 mL water, and the fraction F2 (150.0 mg) was ultrasonically dissolved in 10.0 mL methanol to afford the tested solutions. Prior to injection for analysis, all solutions were centrifuged at 17,709× g for 10 min, and the supernatants were then transferred into sealed vials and stored at 4 °C before use.

UPLC/IM-QTOF-MS Analysis was performed on an ACQUITY UPLC I-Class/Vion IMS-QTOF high-resolution LC-MS system (Waters Corporation, Milford, MA, USA). Chromatographic separation was achieved on a COSMOSIL PBr column (2.1 mm × 100 mm, 2.6 μm) at 30 °C. The binary mobile phase composed of 0.1% formic acid in water (A) and methanol (B) ran at a flow rate of 0.3 mL/min. The injection volume was 5 μL and pre-equilibrated for 10 min. The elution program as the following: 0–5 min, 0%–5% B; 5–9 min, 5%–30% B; 9–12 min, 30%–33% B; 12–19 min, 33%–50% B; 19–28 min, 50%–81% B; 28–30 min, 81%–100% B; 30–33 min, 100% B, 33–36 min, 0% B; 36–40 min, 0% B. All samples were injected once in negative ion mode.

High-resolution MS data were recorded on a Vion IMS-Q-TOF mass spectrometer (Waters Corporation, MA, USA). The ion source parameters were set as follows: Capillary Voltage, –1.0

kV; Cone Voltage, 40 kV; Source Offset, 80 V; Source temperature, 120 °C; Desolvation gas temperature, 500 °C; Desolvation gas flow (N_2), 800 L/h; Cone gas flow (N_2), 50 L/h. Default parameters were defined for the travelling wave IM separation, and CCS calibration was conducted according to the manufacture's guidelines using a mixture of calibrants. Data acquisition was controlled by the UNIFI 1.9.3.0 software (Waters Corporation, Milford, MA, USA).

S3. Evaluation of antioxidant capacity

Samples **S1**–**S12** are whole fruits with hard shells, so they were broken up in batches and the kernels were removed. Then eighteen batches of CF (**S1**−**S18**) sample were pulverized (BJ-800A, Hangzhou Baijie Technology Co., Ltd., Hangzhou, China) and sieved through 50 mesh. 2.0 g powder of sample was accurately weighed and ultrasonically extracted (600 W, Zhixin Instrument Co., Ltd., Shanghai, China) with 500 mL methanol for 20 min, respectively, then concentrated before drying in vacuum at 40 °C by rotary evaporator (Rotavapor R-250, BUCHI, Switzerland) to afford the methanol extracts (CFMEs).

S3.1 DPPH• Radical scavenging assay

Each CFME was dissolved in methanol and subjected to a stepwise dilution to eight final concentrations (50.0000, 25.0000, 12.5000, 6.2500, 3.1250, 1.5625, 0.7813, 0.3906 μg/mL). One hundred microliter of CFME solution was mixed with 100 μL of 0.2 mM DPPH$^\bullet$ solution dissolved in methanol in 96 well plates. The mixture was allowed to stand in the dark for 30 min, and its absorbance was measured at 517 nm on the SPARK® multimode microplate reader (Tecan laboratory equipment Co., Ltd., Switzerland). The DPPH$^\bullet$ scavenging percentage was determined according to Equation (1).

$$\text{DPPH}^\bullet \text{ scavenging percentage} = [1 - (A_{\text{sample}} - A_{\text{blank}})/A_{\text{control}}] \times 100\% \quad (1)$$

where A_{control}, A_{blank}, and A_{sample} are the absorbance of 100 μL DPPH$^\bullet$ solution mixed with 100 μL methanol, 100 μL methanol mixed with 100 μL sample solution, and 100 μL DPPH$^\bullet$ solution mixed with 100 μL sample solution, respectively. The IC_{50} value shows that the concentration of the tested sample solution inhibiting 50% DPPH radical.

S3.2 UPLC-DPPH• Analysis

CFME solution (**S5**, 1.0 mg/mL) and DPPH$^\bullet$ (1.0 mmol/L) were mixed in proportion (1:1) as the DPPH•-pretreated sample, while CFME solution mixed with equal volume of methanol was prepared as the control sample (n = 6). Both the DPPH$^\bullet$-pretreated sample and the control sample were then incubated in the dark at room temperature for 30 min and centrifuged at 17,709 g for 10 min before injection into the Waters H-Class plus UPLC-PDA system (Milford, MA, USA) equipped with a COSMOSIL PBr column (2.1 mm × 100 mm, 2.6 μm), with chromatographic and detection conditions optimized as follows: Chromatographic separation was performed on at 30 ℃. The mobile phase was 0.1% formic acid in water (A) and methanol (B) at a flow rate of 0.3 mL/min, and the gradient elution program was set as: 0–5 min, 0%–5% B; 5–9 min, 5%–30% B; 9–12 min, 30%–33% B; 12–19 min, 33%–50% B; 19–28 min, 50%–81% B; 28–30 min, 81%–100% B; 30–33 min, 100% B. The injection volume was 2 μL. The ultraviolet absorption spectra of all the samples were recorded in the range of 190–400 nm, and the detection wavelength was set at 254 nm and 270 nm as same as the established fingerprinting method.

S3.3 ABTS$^+$ Radical scavenging assay

The ABTS$^+$ radical scavenging activity of CFME was evaluated by an ABTS$^+$ radical scavenging assay kit (Beyotime Institute of Biotechnology, Shanghai, China). ABTS Solution (1.0 mL) and oxidizing agent (1.0 mL) were mixed to produce ABTS$^+$ solution, and the mixture was kept in the dark at room temperature for 14 h. Before use, 78.0 mL 80% ethanol was added to the resulting ABTS$^+$ solution to obtain working solution. Ten microliters of Trolox standard solutions in various concentrations, tested sample (0.05 mg/mL), and methanol (blank group) were added into 180 μL of ABTS$^+$ working solution, respectively, and react for 6 min in the dark at room temperature, before being recorded at 734 nm. The total antioxidant capacity of each tested sample was finally calculated based on the calibration curve and represented by the Trolox-Equivalent Antioxidant Capacity (TEAC).

S3.4 Ferric reducing antioxidant power (FRAP)

The antioxidant capacity of CFME was further assessed by a FRAP method (Beyotime Institute of Biotechnology, Shanghai, China) with minor revisions. A working solution was prepared by mixing TPTZ dilution, detective buffer, and TPTZ solution in a ratio of 10:1:1 (*v*/*v*), and then the working solution was incubated in a water bath at 37 °C. Five microliters of calibration solution, tested sample, and blank were added into 180 μL of working solution, respectively, and kept at 37 °C for 5 min. The absorbance was measured at 593 nm for 6 min. The ferric reducing antioxidant power of each tested sample was calculated from the linear calibration curve and expressed as millimole $FeSO_4$ per gram of the dried extracts.

Table S1. Information of the 18 batches of CF.

ID.	Variety	Part	Origin
**S1**	*Terminalia cheubla* Retz.	Fruit	India
**S2**	*Terminalia cheubla* Retz.	Fruit	India
**S3**	*Terminalia cheubla* Retz.	Fruit	India
**S4**	*Terminalia cheubla* Retz.	Fruit	India
**S5**	*Terminalia cheubla* Retz.	Fruit	India
**S6**	*Terminalia cheubla* Retz.	Fruit	India
**S7**	*Terminalia cheubla* Retz.	Fruit	Bozhou, China
**S8**	*Terminalia cheubla* Retz.	Fruit	Bozhou, China
**S9**	*Terminalia cheubla* Retz.	Fruit	Bozhou, China
**S10**	*Terminalia cheubla* Retz.	Fruit	India
**S11**	*Terminalia cheubla* Retz.	Fruit	Yunnan, China
**S12**	*Terminalia cheubla* Retz.	Fruit	Tibet, China
**S13**	*Terminalia cheubla* Retz.	Flesh	Myanmar
**S14**	*Terminalia chebula* Retz.	Flesh	Myanmar
**S15**	*Terminalia cheubla* Retz.	Flesh	Yunnan, China
**S16**	*Terminalia cheubla* Retz.	Flesh	Yunnan, China
**S17**	*Terminalia cheubla* Retz.	Flesh	Guangxi, China
**S18**	*Terminalia cheubla* Retz.	Flesh	Guangxi, China

Table S2. Composition of the mixed standard solution.

No.	Standard compounds	Concentration (μg/mL)
1	chebulic acid	250.4
2	gallic acid	105.4
3	4-*O*-galloyl-(-)-shikimic acid	25.55
4	5-*O*-galloyl-(-)-shikimic acid	33.24
5	punicalagin A and B	121.4
6	corilagin	32.76
7	1,3,6-tri-*O*-galloyl-*β*-D-glucose	34.16
8	chebulanin	11.54
9	urolithin M5	17.85
10	chebulagic acid	91.80
11	chebulinic acid	93.20
12	ellagic acid	87.66
13	terminalin	12.75
14	4-*O*-(3",4"-di-*O*-galloyl-*α*-L-rhamnosyl)ellagic acid	24.33

Table S3. The currently reported constituents collated in our in-house compound library of CF.

Serial No.	Compound name	CAS No.[#]	Molecular formula	Exact mass
1	punicalagin	65995-63-3	$C_{48}H_{28}O_{30}$	1084.0665
2	terflavin A	103744-89-4	$C_{48}H_{30}O_{30}$	1086.0822
3	chebulanin	166833-80-3	$C_{27}H_{24}O_{19}$	652.0912
4	corilagin	23094-69-1	$C_{27}H_{22}O_{18}$	634.0806
5	geraniin	60976-49-0	$C_{41}H_{28}O_{27}$	952.0818
6	chebulagic acid	23094-71-5	$C_{41}H_{30}O_{27}$	954.0974
7	chebulinic acid	18942-26-2	$C_{41}H_{32}O_{27}$	956.1131
8	ellagic acid	476-66-4	$C_{14}H_6O_8$	302.0063
9	methyl gallate	99-24-1	$C_8H_8O_5$	184.0372
10	2,3-(*S*)-HHDP-6-*O*-galloyl-D-glucose	–	$C_{27}H_{22}O_{18}$	634.0806
11	3,6-di-*O*-galloyl-D-glucose	–	$C_{20}H_{20}O_{14}$	484.0853
12	4,6-di-*O*-galloyl-D-glucose	–	$C_{20}H_{20}O_{14}$	484.0853
13	tellimagrandin II	81571-72-4	$C_{41}H_{30}O_{26}$	938.1025
14	1-*O*-galloyl-*β*-D-glucose	58511-73-2	$C_{13}H_{16}O_{10}$	332.0743
15	1,3,6-tri-*O*-galloyl-*β*-D-glucose	18483-17-5	$C_{27}H_{24}O_{18}$	636.0963
16	1,2,3,4,6-penta-*O*-galloyl-*β*-D-glucose	14937-32-7	$C_{41}H_{32}O_{26}$	940.1182
17	4-*O*-(3'',4''-di-*O*-galloyl)-*α*-L-rhamnosyl-ellagic acid	–	$C_{34}H_{24}O_{20}$	752.0861
18	urolithin M5	91485-02-8	$C_{13}H_8O_7$	276.0270
19	5-*O*-galloylshikimic acid	95719-51-0	$C_{14}H_{14}O_9$	326.0638
20	gallic acid	149-91-7	$C_7H_6O_5$	170.0215
21	punicalin	65995-64-4	$C_{34}H_{22}O_{22}$	782.0603
22	terchebulin	132854-40-1	$C_{48}H_{28}O_{30}$	1084.0665
23	casuarinin	79786-01-9	$C_{41}H_{28}O_{26}$	936.0869
24	1,2,3-tri-*O*-galloyl-6-*O*-cinnamoyl-*β*-D-glucose	–	$C_{36}H_{30}O_{19}$	766.1381

25	1,2,3,6-tetra-*O*-galloyl-4-*O*-cinnamoyl-*β*-D-glucose	–	$C_{43}H_{34}O_{23}$	918.1491
26	1,6-di-*O*-galloyl-2-*O*-cinnamoyl-*β*-D-glucose	–	$C_{29}H_{26}O_{15}$	614.1272
27	1,2-di-*O*-galloyl-6-*O*-cinnamoyl-*β*-D-glucose	–	$C_{29}H_{26}O_{15}$	614.1272
28	4-*O*-(2",4"-di-*O*-galloyl-*α*-L-rhamnosyl)ellagic acid	–	$C_{34}H_{24}O_{20}$	752.0861
29	eschweilenol C	211371-02-7	$C_{20}H_{16}O_{12}$	448.0642
30	4-*O*-(4"-*O*-galloyl-*α*-L-rhamnosyl)ellagic acid	–	$C_{27}H_{20}O_{16}$	600.0751
31	1'-*O*-methyl neochebulanin	–	$C_{28}H_{28}O_{20}$	684.1174
32	dimethyl neochebulinate	–	$C_{43}H_{38}O_{28}$	1002.1550
33	phyllanemblinin E	–	$C_{27}H_{26}O_{20}$	670.1017
34	1'-*O*-methyl neochebulinate	–	$C_{42}H_{36}O_{28}$	988.1393
35	6'-*O*-methyl neochebulagate	–	$C_{42}H_{34}O_{28}$	986.1237
36	dimethyl neochebulagate	–	$C_{43}H_{36}O_{28}$	1000.1393
37	neochebulagic acid	–	$C_{41}H_{32}O_{28}$	972.1080
38	dimethyl 4'-*epi*-neochebulagate	–	$C_{43}H_{36}O_{28}$	1000.1393
39	methyl chebulagate	–	$C_{42}H_{32}O_{27}$	968.1131
40	phyllanemblinin F	–	$C_{27}H_{26}O_{20}$	670.1017
41	chebulic acid	23725-05-5	$C_{15}H_{14}O_{10}$	356.0380
42	6'-*O*-methyl chebulate	–	$C_{16}H_{16}O_{10}$	368.0743
43	7'-*O*-methyl chebulate	–	$C_{16}H_{16}O_{10}$	368.0743
44	4-*O*-galloylshikimic acid	110082-90-1	$C_{14}H_{14}O_{9}$	326.0638
45	1,3-di-*O*-galloyl-*β*-D-glucose	–	$C_{20}H_{20}O_{14}$	484.0853
46	1,6-di-*O*-galloyl-*β*-D-glucose	23363-08-8	$C_{20}H_{20}O_{14}$	484.0853
47	1,2,3,6-tetra-*O*-galloyl-*β*-D-glucose	–	$C_{34}H_{28}O_{22}$	788.1072
48	1,3,4,6-tetra-*O*-galloyl-*β*-D-glucose	–	$C_{34}H_{28}O_{22}$	788.1072
49	6-*O*-galloyl-D-glucose	13186-19-1	$C_{13}H_{16}O_{10}$	332.0743
50	3,4,6-tri-*O*-galloyl-D-glucose	–	$C_{27}H_{24}O_{18}$	636.0963

51	tercatain	103744-87-2	$C_{34}H_{26}O_{22}$	786.0916
52	gemin D	84744-46-7	$C_{27}H_{22}O_{18}$	634.0806
53	tellimagrandin I	–	$C_{34}H_{26}O_{22}$	786.0916
54	digallic acid	536-08-3	$C_{14}H_{10}O_{9}$	322.0325
55	brevifolin carboxylic acid	18490-95-4	$C_{13}H_{8}O_{8}$	292.0219
56	punicacortein C	103488-37-5	$C_{48}H_{28}O_{30}$	1084.0665
57	punicacortein D	103488-37-5	$C_{48}H_{28}O_{30}$	1084.0665
58	chebumeinin B	–	$C_{27}H_{26}O_{20}$	670.1017
59	lithospermate B	122021-74-3	$C_{36}H_{30}O_{16}$	718.1534
60	tri-*n*-butyl chebulate	–	$C_{26}H_{36}O_{11}$	524.2258
61	methyl (*S*)-flavogallonate	–	$C_{22}H_{12}O_{13}$	484.0278
62	methyl neochebulagate	–	$C_{42}H_{34}O_{28}$	986.1237
63	isoterchebulin	–	$C_{48}H_{28}O_{29}$	1068.0716
64	ethyl gallate	831-61-8	$C_{9}H_{10}O_{5}$	198.0528
65	4-*O*-methylgallic acid	4319-02-2	$C_{8}H_{8}O_{5}$	184.0372
66	ferulic acid	1135-24-6	$C_{10}H_{10}O_{4}$	194.0579
67	vanillic acid	121-34-6	$C_{8}H_{8}O_{4}$	168.0423
68	*p*-Coumaric acid	501-98-4	$C_{9}H_{8}O_{3}$	164.0473
69	eugenol	97-53-0	$C_{10}H_{12}O_{2}$	164.0837
70	caffeic acid	331-39-5	$C_{9}H_{8}O_{4}$	180.0423
71	melilotic acid	495-78-3	$C_{9}H_{10}O_{3}$	166.0630
72	phloroglucinol	108-73-6	$C_{6}H_{6}O_{3}$	126.0317
73	pyrogallol	87-66-1	$C_{6}H_{6}O_{3}$	126.0317
74	protocatechuic acid	99-50-3	$C_{7}H_{6}O_{4}$	154.0266
75	rutin	153-18-4	$C_{27}H_{30}O_{16}$	610.1534
76	quercetin	117-39-5	$C_{15}H_{10}O_{7}$	302.0427

77	luteolin	491-70-3	$C_{15}H_{10}O_6$	286.0477
78	isoquercetin	482-35-9	$C_{21}H_{20}O_{12}$	464.0955
79	3-methoxy quercetin	480-19-3	$C_{16}H_{12}O_7$	316.0583
80	3,4'-dimethoxy quercetin	3306-29-4	$C_{17}H_{14}O_7$	330.0740
81	23-*O*-neochebuloylarjungenin 28-*O*-*β*-D-glycopyranosyl ester	–	$C_{50}H_{68}O_{21}$	1004.4253
82	23-*O*-4'-*epi*-neochebuloylarjungenin	–	$C_{44}H_{58}O_{16}$	842.3725
83	arjungenin	58880-25-4	$C_{30}H_{48}O_6$	504.3451
84	23-*O*-galloylarjunic acid	–	$C_{37}H_{52}O_{10}$	656.3560
85	arjunglucoside I	62319-70-4	$C_{36}H_{58}O_{11}$	666.3979
86	quercotriterpenoside I	–	$C_{43}H_{62}O_{15}$	818.4089
87	terminolic acid	564-13-6	$C_{30}H_{48}O_6$	504.3451
88	23-*O*-galloylterminolic acid 28-*O*-*β*-D-glucopyranosyl ester	–	$C_{43}H_{62}O_{15}$	818.4089
89	arjunolic acid	465-00-9	$C_{30}H_{48}O_5$	488.3502
90	arjunglucoside II	62369-72-6	$C_{36}H_{58}O_{10}$	650.4030
91	23-*O*-galloylarjunolic acid	–	$C_{37}H_{52}O_9$	640.3611
92	23-*O*-galloylarjunolic acid 28-*O*-*β*-D-glucopyranosyl ester	–	$C_{43}H_{62}O_{14}$	802.4140
93	arjunic acid	31298-06-3	$C_{30}H_{48}O_5$	488.3502
94	arjunetin	–	$C_{36}H_{58}O_{10}$	650.4030
95	crataegioside	–	$C_{36}H_{58}O_{10}$	650.4030
96	pinfaenoic acid 28-*O*-*β*-D-glucopyranosylester	–	$C_{36}H_{56}O_{10}$	648.3873
97	23-*O*-galloylpinfaenoic acid 28-*O*-*β*-D-glucopyranosyl ester	–	$C_{43}H_{60}O_{14}$	800.3983
98	chebuloside II	149475-28-5	$C_{36}H_{58}O_{11}$	666.3979
99	*β*-sitosterol	76772-70-8	$C_{29}H_{50}O$	414.3862
100	terflavin B	103744-86-1	$C_{34}H_{24}O_{22}$	784.0759
101	terflavin C	–	$C_{41}H_{26}O_{26}$	934.0712
102	sitogluside	474-58-8	$C_{35}H_{60}O_6$	576.4390

103	shikimic acid	138-59-0	$C_7H_{10}O_5$	174.0528
104	terflavin D	–	$C_{27}H_{20}O_{18}$	632.0650
105	chebumeinin A	–	$C_{27}H_{26}O_{20}$	670.1017
106	(-)-chebulic acid triethyl ester	–	$C_{20}H_{24}O_{11}$	440.1319
107	terchebin	20598-45-2	$C_{41}H_{30}O_{27}$	954.0974
108	maslinic acid	4373-41-5	$C_{30}H_{48}O_4$	472.3553
109	chebupentol	143086-38-8	$C_{30}H_{50}O_5$	490.3658
110	sennoside A	81-27-6	$C_{42}H_{38}O_{20}$	862.1956
111	termitomenin A	–	$C_{19}H_{22}O_4$	314.1518
112	termitomenin B	–	$C_{20}H_{24}O_5$	344.1624
113	termitomenin C	–	$C_{18}H_{20}O_4$	300.1362
114	termitomenin D	–	$C_{26}H_{28}O_{12}$	532.1581
115	termitomenin E	–	$C_{32}H_{38}O_{18}$	710.2058
116	*erythro*-guaiacylglycerol-*β*-coniferyl aldehyde ether	–	$C_{20}H_{22}O_7$	374.1366
117	(−)-balanophonin	80286-36-8	$C_{20}H_{20}O_6$	356.1260
118	rel-(2*α*,3*β*)-7-*O*-methylcedrusin	–	$C_{20}H_{24}O_6$	360.1573
119	(7*S*,8*R*)-3,3′,5-trimethoxy-4′,7-epoxy-8,5′-neolignan-4,9,9′-triol	–	$C_{24}H_{32}O_9$	464.2046
120	dehydrodiconiferyl alcohol	4263-87-0	$C_{20}H_{22}O_6$	358.1416
121	sesquimarocanol B	–	$C_{30}H_{38}O_{10}$	558.2465
122	(+)-(7*S*,8*S*,8'*S*)-9-*O*-[*β*-D-glucopyranoyl]asarininone	–	$C_{26}H_{28}O_{12}$	532.1581
123	3-methoxy-3′,4′-(methyl-enedioxy)-9,9-epoxylignan-4,7-diol	–	$C_{20}H_{22}O_6$	358.1416
124	(+)-pinoresinol	487-36-5	$C_{20}H_{22}O_6$	358.1416
125	(+)-(7*R*,7′*R*,7″*R*,7‴*R*,8*S*,8′*S*,8″*S*,8‴*S*)-4″,4‴-dihydroxy-3,3′,3″,3‴,5,5′-hexamethoxy-7,9′:7′,9-diepoxy-4,8″:4′,8‴-bisoxy-8,8′-dineolignan-7″,7‴,9″,9‴-tetraol	–	$C_{42}H_{50}O_{16}$	810.3099
126	termitomenins F	–	$C_{27}H_{32}O_{12}$	548.1894
127	termitomenins G	–	$C_{28}H_{34}O_{13}$	578.1999

128	tri-*O*-galloylshikimic acid	129159-07-5	$C_{28}H_{22}O_{17}$	630.0857
129	1,2,6-tri-*O*-galloyl-*β*-D-glucopyranose	–	$C_{27}H_{24}O_{18}$	636.0963
130	1,2,3,4-tetra-*O*-galloyl-*β*-D-glucose	–	$C_{34}H_{28}O_{22}$	788.1072
131	2,3,4,6-tetra-*O*-galloyl-D-glucose	40410-95-5	$C_{34}H_{28}O_{22}$	788.1072
132	2,4-chebuloyl-*β*-D-glucopyranoside	–	$C_{20}H_{20}O_{15}$	500.0802
133	3'-*O*-methyl-4-*O*-(3",4"-di-*O*-galloyl-*α*-L-rhamnopyranosyl) ellagic acid	–	$C_{35}H_{26}O_{20}$	766.1017
134	1,3-di-*O*-galloyl-2,4-chebuloyl-*β*-D-glucose	–	$C_{34}H_{28}O_{23}$	804.1021
135	1,6-di-*O*-galloyl-2,4-chebuloyl-*β*-D-glucose	–	$C_{34}H_{28}O_{23}$	804.1021
136	neochebulinic acid	–	$C_{41}H_{34}O_{28}$	974.1237
137	3-*O*-galloyl-(-)-shikimic acid	110080-91-2	$C_{14}H_{14}O_{9}$	326.0638
138	neochebulic acid	–	$C_{15}H_{14}O_{10}$	356.0380
139	methyl shikimate	40983-58-2	$C_{8}H_{12}O_{5}$	188.0685
140	terminaliate A	–	$C_{13}H_{10}O_{7}$	278.0427

[#] –, not available

Table S4. Identification of constituents by in-house compound library search.

No.	t_R (min)	Molecular weight	*m/z*	Quasi-molecular /Adduct ion	Molecular formula	Base peak	Peak height	Identification	Error (ppm)
**2**	1.55	174.0534	173.0461	$[M–H]^–$	$C_7H_{10}O_5$	93.0344	136708	shikimic acid	3.24
**12**	8.39	170.0223	169.0150	$[M–H]^–$	$C_7H_6O_5$	51.0245	182097	gallic acid*	4.55
**15**	9.13	332.0750	331.0677	$[M–H]^–$	$C_{13}H_{16}O_{10}$	59.0142	372487	galloyl-D-glucose	1.99
**18**	10.04	332.0747	331.0674	$[M–H]^–$	$C_{13}H_{16}O_{10}$	125.0244	296048	galloyl-D-glucose	1.11
**22**	10.88	332.0749	331.0677	$[M–H]^–$	$C_{13}H_{16}O_{10}$	124.0168	153598	galloyl-D-glucose	1.78
**30**	12.47	326.0648	325.0575	$[M–H]^–$	$C_{14}H_{14}O_9$	124.0167	823174	4-*O*-galloyl-(-)-shikimic acid*	3.00
**31**	12.71	782.0614	781.0542	$[M–H]^–$	$C_{34}H_{22}O_{22}$	600.9899	353257	punicalin	1.42
**33**	13.08	326.0646	325.0573	$[M–H]^–$	$C_{14}H_{14}O_9$	125.0245	860458	5-*O*-galloyl-(-)-shikimic acid*	2.40
**34**	13.36	326.0644	325.0572	$[M–H]^–$	$C_{14}H_{14}O_9$	125.0245	747456	galloyl-(-)-shikimic acid	1.90
**35**	13.50	484.0858	483.0786	$[M–H]^–$	$C_{20}H_{20}O_{14}$	169.0143	175454	di-*O*-galloyl-D-glucose	0.99
**38**	14.32	484.0856	483.0784	$[M–H]^–$	$C_{20}H_{20}O_{14}$	169.0143	101841	di-*O*-galloyl-D-glucose	0.54
**39**	14.79	1084.0663	1083.0591	$[M–H]^–$	$C_{48}H_{28}O_{30}$	1083.0576	75525	punicalagin A	-0.25
**40**	15.00	670.1018	334.0437	$[M–2H]^{2–}$	$C_{27}H_{26}O_{20}$	59.0142	21958	phyllanemblinin E	0.03
**41**	15.23	484.0862	483.0790	$[M–H]^–$	$C_{20}H_{20}O_{14}$	169.0144	273571	di-*O*-galloyl-D-glucose	1.75
**45**	16.07	198.0531	197.0459	$[M–H]^–$	$C_9H_{10}O_5$	78.0114	48087	ethyl gallate	1.57

**47**	16.60	1084.0688	541.0274	$[M–2H]^{2–}$	$C_{48}H_{28}O_{30}$	300.9987	352373	punicalagin B	2.13
**48**	16.78	484.0859	483.0787	$[M–H]^{–}$	$C_{20}H_{20}O_{14}$	169.0142	105679	di-*O*-galloyl-D-glucose	1.16
**49**	17.07	484.0861	483.0789	$[M–H]^{–}$	$C_{20}H_{20}O_{14}$	169.0144	150585	di-*O*-galloyl-D-glucose	1.58
**51**	17.33	684.1184	683.1113	$[M–H]^{–}$	$C_{28}H_{28}O_{20}$	169.0145	317874	1'-*O*-methyl neochebulanin	1.47
**56**	18.42	634.0825	633.0749	$[M–H]^{–}$	$C_{27}H_{22}O_{18}$	300.9995	1969666	corilagin*	3.02
**57**	18.62	786.0893	392.0374	$[M–2H]^{2–}$	$C_{34}H_{26}O_{22}$	125.0237	5097	tercatain or its isomer	-2.85
**59**	19.05	972.1076	485.0462	$[M–2H]^{2–}$	$C_{41}H_{32}O_{28}$	300.9988	5131	neochebulagic acid	-0.40
**60**	19.16	1086.0799	1085.0727	$[M–H]^{–}$	$C_{48}H_{30}O_{30}$	450.9938	20712	terflavin A	-2.10
**61**	19.27	786.0914	785.0843	$[M–H]^{–}$	$C_{34}H_{26}O_{22}$	300.9986	95274	tellimagrandin I	-0.27
**62**	19.42	684.1182	341.0519	$[M–2H]^{2–}$	$C_{28}H_{28}O_{20}$	125.0243	5238	1'-*O*-methyl neochebulanin isomer	1.17
**64**	20.01	292.0221	291.0149	$[M–H]^{–}$	$C_{13}H_{8}O_{8}$	145.0293	50137	brevifolin carboxylic acid	0.68
**65**	20.04	786.0907	785.0833	$[M–H]^{–}$	$C_{34}H_{26}O_{22}$	300.9984	7476	tellimagrandin I	-1.05
**68**	20.45	636.0951	317.0402	$[M–2H]^{2–}$	$C_{27}H_{24}O_{18}$	125.0245	1741	1,3,6-tri-*O*-galloyl-D-glucose*	-1.75
**69**	20.83	636.0972	635.0900	$[M–H]^{–}$	$C_{27}H_{24}O_{18}$	169.0144	129894	tri-*O*-galloyl-D-glucose	1.40
**75**	21.47	788.1067	787.0995	$[M–H]^{–}$	$C_{34}H_{28}O_{22}$	169.0141	40658	tetra-*O*-galloyl-β-D-glucose	-0.66
**78**	22.28	986.1241	492.0549	$[M–2H]^{2–}$	$C_{42}H_{34}O_{28}$	125.0245	72002	methyl neochebulagate	0.42
**80**	22.40	652.0928	325.0392	$[M–2H]^{2–}$	$C_{27}H_{24}O_{19}$	125.0244	143738	chebulanin*	2.49

**86**	23.28	276.0279	275.0206	[M–H]$^{–}$	$C_{13}H_{8}O_{7}$	145.0296	282799	urolithin M5*	3.13
**89**	23.61	788.1083	787.1013	[M–H]$^{–}$	$C_{34}H_{28}O_{22}$	169.0144	348048	tetra-*O*-galloyl-β-D-glucose	1.39
**92**	24.19	788.1081	787.1010	[M–H]$^{–}$	$C_{34}H_{28}O_{22}$	169.0145	26178	tetra-*O*-galloyl-β-D-glucose	1.11
**95**	24.98	954.1001	476.0429	[M–2H]$^{2–}$	$C_{41}H_{30}O_{27}$	125.0246	643427	chebulagic acid*	2.77
**97**	25.74	940.1198	469.0527	[M–2H]$^{2–}$	$C_{41}H_{32}O_{26}$	125.0245	288999	1,2,3,4,6-penta-*O*-galloyl-β-D-glucose	1.68
**98**	26.26	988.1398	987.1328	[M–H]$^{–}$	$C_{42}H_{36}O_{28}$	205.0506	163564	1'-*O*-methyl neochebulinate	0.50
**99**	26.26	610.1531	609.1459	[M–H]$^{–}$	$C_{27}H_{30}O_{16}$	300.0275	43804	rutin	-0.43
**107**	28.52	956.1146	955.1073	[M–H]$^{–}$	$C_{41}H_{32}O_{27}$	275.0200	1288328	chebulinic acid*	1.58
**110**	28.99	302.0073	301.0001	[M–H]$^{–}$	$C_{14}H_{6}O_{8}$	145.0296	2023036	ellagic acid*	3.38
**111**	29.31	448.0648	447.0576	[M–H]$^{–}$	$C_{20}H_{16}O_{12}$	299.9916	622091	eschweilenol C*	1.32
**115**	30.60	504.3459	503.3388	[M–H]$^{–}$	$C_{30}H_{48}O_{6}$	503.3381	192462	terminolic acid	1.58
**117**	31.42	504.3450	549.3434	[M+HCOOH-H]$^{–}$	$C_{30}H_{48}O_{6}$	503.3374	73203	terminolic acid	-0.11
**118**	31.53	600.0768	599.0697	[M–H]$^{–}$	$C_{27}H_{20}O_{16}$	300.9993	348037	4-*O*-(4"-*O*-galloyl-α-L-rhamnosyl)ellagic acid	2.81
**121**	32.47	752.0859	375.0357	[M–2H]$^{2–}$	$C_{34}H_{24}O_{20}$	125.0244	29954	4-*O*-(3",4"-di-*O*-galloyl-α-L-rhamnosyl)ellagic acid*	-0.23
**122**	32.51	752.0866	751.0795	[M–H]$^{–}$	$C_{34}H_{24}O_{20}$	300.9991	597246	4-*O*-(2",4"-di-*O*-galloyl-α-L-rhamnosyl)ellagic acid	0.69

* The constituents identified by comparisons with the standard compounds.

Table S5. Calculated similarities for 18 batches of CF.

Sample	S1	S2	S3	S4	S5	S6	S7	S8	S9	S10	S11	S12	S13	S14	S15	S16	S17	S18	Reference
**S1**	1	0.995	0.999	0.998	0.991	0.778	0.804	0.728	0.752	0.736	0.759	0.74	0.642	0.696	0.43	0.457	0.713	0.5	0.89
**S2**	0.995	1	0.991	0.996	0.985	0.724	0.749	0.671	0.695	0.678	0.701	0.682	0.601	0.666	0.384	0.408	0.685	0.458	0.846
**S3**	0.999	0.991	1	0.997	0.992	0.792	0.819	0.743	0.768	0.753	0.774	0.756	0.648	0.702	0.44	0.468	0.718	0.509	0.9
**S4**	0.998	0.996	0.997	1	0.986	0.764	0.787	0.712	0.738	0.72	0.741	0.722	0.612	0.67	0.394	0.422	0.69	0.468	0.873
**S5**	0.991	0.985	0.992	0.986	1	0.764	0.804	0.718	0.743	0.731	0.756	0.739	0.716	0.771	0.525	0.549	0.786	0.591	0.904
**S6**	0.778	0.724	0.792	0.764	0.764	1	0.989	0.994	0.996	0.994	0.992	0.99	0.563	0.573	0.415	0.457	0.583	0.474	0.939
**S7**	0.804	0.749	0.819	0.787	0.804	0.989	1	0.984	0.99	0.99	0.995	0.993	0.672	0.677	0.533	0.571	0.68	0.588	0.973
**S8**	0.728	0.671	0.743	0.712	0.718	0.994	0.984	1	0.997	0.997	0.994	0.995	0.562	0.567	0.427	0.468	0.576	0.486	0.924
**S9**	0.752	0.695	0.768	0.738	0.743	0.996	0.99	0.997	1	0.998	0.997	0.996	0.581	0.587	0.44	0.481	0.595	0.501	0.937
**S10**	0.736	0.678	0.753	0.72	0.731	0.994	0.99	0.997	0.998	1	0.998	0.998	0.593	0.597	0.462	0.503	0.603	0.518	0.936
**S11**	0.759	0.701	0.774	0.741	0.756	0.992	0.995	0.994	0.997	0.998	1	0.999	0.635	0.638	0.5	0.538	0.644	0.559	0.952
**S12**	0.74	0.682	0.756	0.722	0.739	0.99	0.993	0.995	0.996	0.998	0.999	1	0.626	0.628	0.498	0.536	0.634	0.556	0.945
**S13**	0.642	0.601	0.648	0.612	0.716	0.563	0.672	0.562	0.581	0.593	0.635	0.626	1	0.986	0.957	0.962	0.974	0.977	0.786
**S14**	0.696	0.666	0.702	0.67	0.771	0.573	0.677	0.567	0.587	0.597	0.638	0.628	0.986	1	0.922	0.929	0.994	0.953	0.804
**S15**	0.43	0.384	0.44	0.394	0.525	0.415	0.533	0.427	0.44	0.462	0.5	0.498	0.957	0.922	1	0.998	0.897	0.988	0.637
**S16**	0.457	0.408	0.468	0.422	0.549	0.457	0.571	0.468	0.481	0.503	0.538	0.536	0.962	0.929	0.998	1	0.905	0.986	0.669
**S17**	0.713	0.685	0.718	0.69	0.786	0.583	0.68	0.576	0.595	0.603	0.644	0.634	0.974	0.994	0.897	0.905	1	0.939	0.81
**S18**	0.5	0.458	0.509	0.468	0.591	0.474	0.588	0.486	0.501	0.518	0.559	0.556	0.977	0.953	0.988	0.986	0.939	1	0.696
**Reference**	0.89	0.846	0.9	0.873	0.904	0.939	0.973	0.924	0.937	0.936	0.952	0.945	0.786	0.804	0.637	0.669	0.81	0.696	1

Table S6. VIP values of twenty three common constituents calculated from OPLS-DA.

**Var ID**	**M2.VIP[2+1+0]**	**Constituents**
**68**	1.1254	1,3,6-tri-*O*-galloyl-*β*-D-glucose
**60**	1.1134	terflavin A
**72**	1.0938	1,2,6-tri-*O*-galloyl-*β*-D-glucose
**47**	1.0911	punicalagin B
**107**	1.0899	chebulinic acid
**95**	1.0235	chebulagic acid
**97**	1.0229	1,2,3,4,6-penta-*O*-galloyl-*β*-D-glucose
**3**	1.0142	chebulic acid
**36**	0.9948	2,3-di-*O*-galloyl-D-glucose
**30**	0.9941	4-*O*-galloyl-(-)-shikimic acid
**118**	0.9887	4-*O*-(4"-*O*-galloyl-*α*-L-rhamnosyl)ellagic acid
**34**	0.9887	3-*O*-galloyl-(-)-shikimic acid
**39**	0.9801	punicalagin A
**12**	0.9796	gallic acid
**89**	0.9733	2,3,4,6-tetra-*O*-galloyl-*β*-D-glucose
**121**	0.9732	4-*O*-(3",4"-di-*O*-galloyl-*α*-L-rhamnosyl)ellagic acid
**86**	0.9703	urolithin M5
**110**	0.9671	ellagic acid
**80**	0.9447	chebulanin
**56**	0.9422	corilagin
**74**	0.9125	3,4-di-*O*-galloylshikimic acid
**33**	0.9094	5-*O*-galloyl-(-)-shikimic acid
**15**	0.8558	1-*O*-galloyl-*β*-D-glucose

Table S7. The antioxidant capacities of the eighteen batches of CF.

Samples	DPPH•[a]	ABTS+[b]	FRAP[c]
S1	5.303	8.477±0.945	1.215±0.009
S2	4.155	13.637±1.044	1.590±0.124
S3	3.600	11.244±0.485	1.396±0.049
S4	4.057	5.870±1.281	0.869±0.067
S5	2.993	14.47±1.106	1.566±0.182
S6	3.465	5.983±0.869	1.069±0.102
S7	4.214	11.291±0.370	1.354±0.137
S8	3.585	20.161±0.313	0.971±0.085
S9	3.326	10.264±0.741	1.273±0.087
S10	3.760	13.375±1.847	0.977±0.090
S11	3.960	10.592±0.547	0.614±0.030
S12	4.519	10.981±1.302	0.747±0.083
S13	8.089	0.470±3.689	1.469±0.069
S14	8.573	1.202±0.278	1.140±0.091
S15	7.487	8.884±1.502	0.911±0.024
S16	5.761	2.318±1.055	0.626±0.063
S17	8.766	2.849±0.449	0.867±0.083
S18	10.110	1.234±1.066	0.643±0.106

[a] DPPH• Radical scavenging activity (IC_{50} values in μg/mL, n = 3); [b] The Trolox-equivalent antioxidant capacity (mmol/g) of the tested sample solution at the concentration of 0.05 mg/mL (n = 6); [c] Ferric reducing antioxidant power (mmol/g) of the tested sample solution at the concentration of 0.5 mg/mL (n = 3).

Table S8. Stability evaluation and the result of UPLC-DPPH$^{\bullet}$ analysis.

No	t_R (min)	Blank (RSD, %)	Tested (RSD, %)	Change of the peak area (%)*	Compounds
**3**	3.75	2.15	1.61	–4.19	chebulic acid
**12**	7.39	1.12	0.75	–8.31	gallic acid
**15**	8.09	4.29	2.43	–4.87	1-*O*-galloyl-*β*-D-glucose
**33**	11.19	2.38	2.74	–18.44	5-*O*-galloyl-(-)-shikimic acid
**34**	11.37	2.19	2.84	1.73	3-*O*-galloyl-(-)-shikimic acid
**38**	12.39	4.66	2.79	–51.03	1,2-di-*O*-galloyl-D-glucose
**39**	12.79	3.80	7.34	–19.66	punicalagin A
**47**	14.49	0.94	4.96	–43.16	punicalagin B
**56**	16.58	1.69	4.07	–31.06	corilagin
**68**	18.17	2.90	2.04	–16.96	1,3,6-tri-*O*-galloyl-*β*-D-glucose
**72**	18.55	1.85	7.15	–31.48	1,2,6-tri-*O*-galloyl-*β*-D-glucose
**80**	19.95	3.60	9.44	–10.68	chebulanin
**86**	21.20	2.00	3.31	–18.48	urolithin M5
**95**	22.65	0.85	2.44	–27.18	chebulagic acid
**97**	23.48	1.92	7.23	–17.98	1,2,3,4,6-penta-galloyl-*O*-*β*-D-glucose
**107**	26.45	1.31	3.73	–22.01	chebulinic acid
**110**	26.78	2.09	3.08	–8.69	ellagic acid
**112**	27.62	1.74	4.39	–9.00	terminalin
**121**	30.86	2.73	2.25	–20.85	4-*O*-(3",4"-di-*O*-galloyl-*α*-L-rhamnosyl)ellagic acid

*Change of the peak area (%) = $[(A_{\text{Tested}} - A_{\text{Blank}})/A_{\text{Blank}}] \times 100\%$, where A_{Tested} and A_{Blank} are the UPLC peak areas of the tested and blank samples.

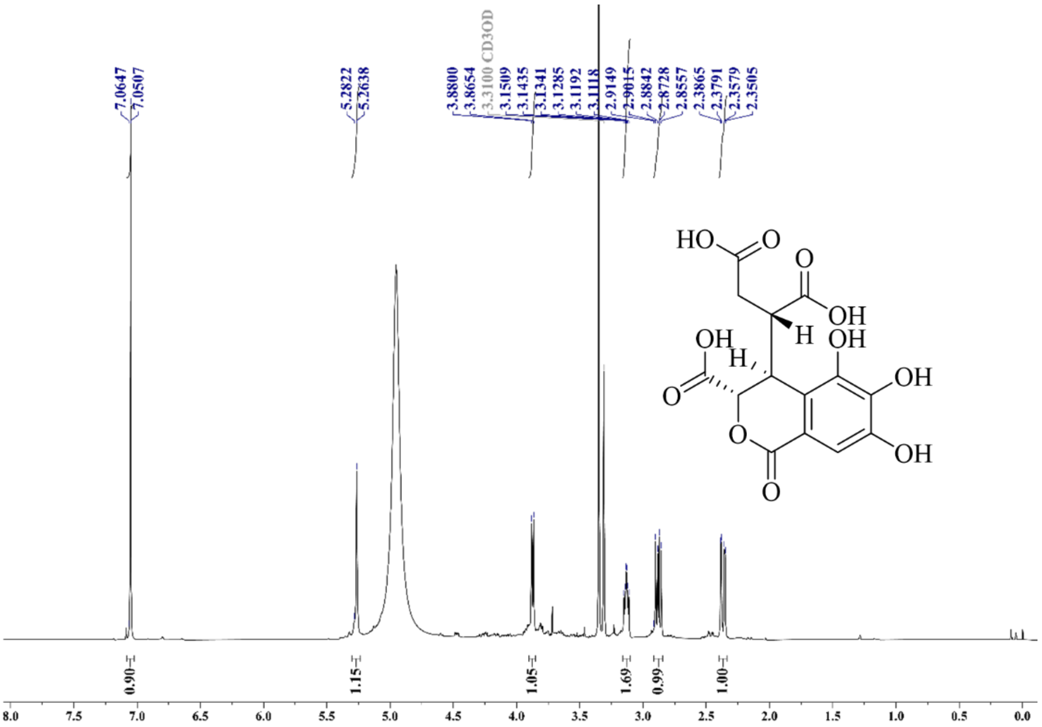

Fig. S1 ^{1}H NMR (600 MHz, CD$_3$OD) spectrum of **chebulic acid**

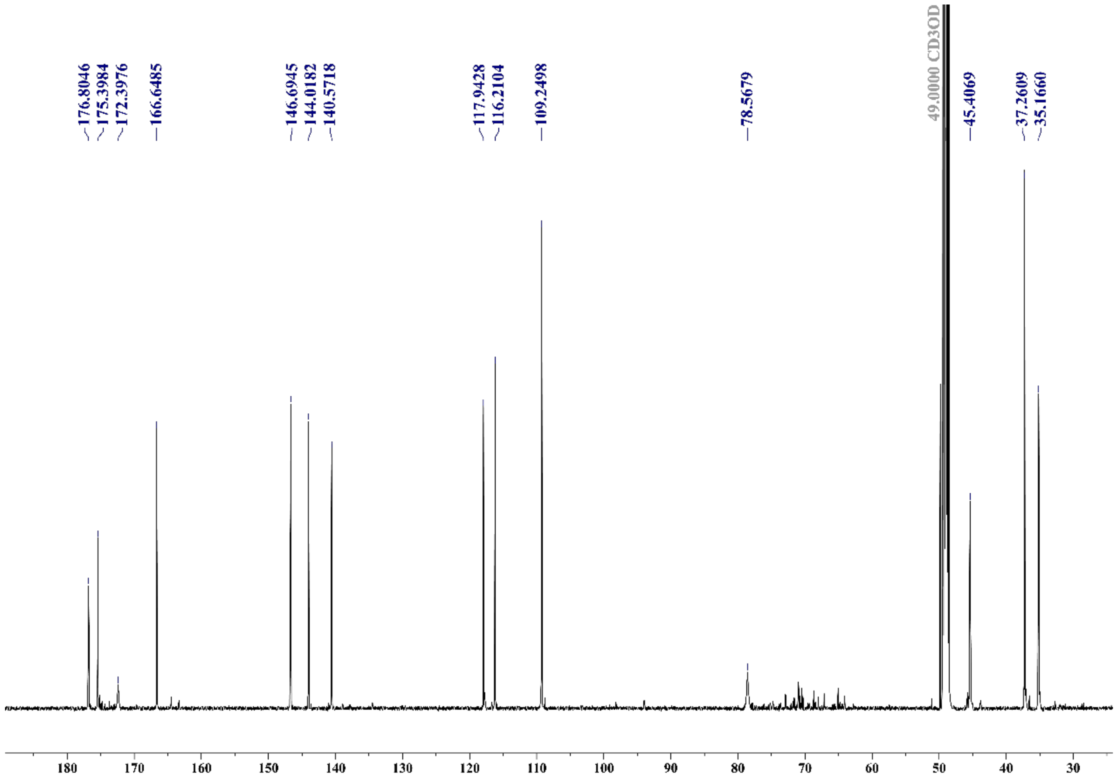

Fig. S2 ^{13}C NMR (150 MHz, CD$_3$OD) spectrum of **chebulic acid**

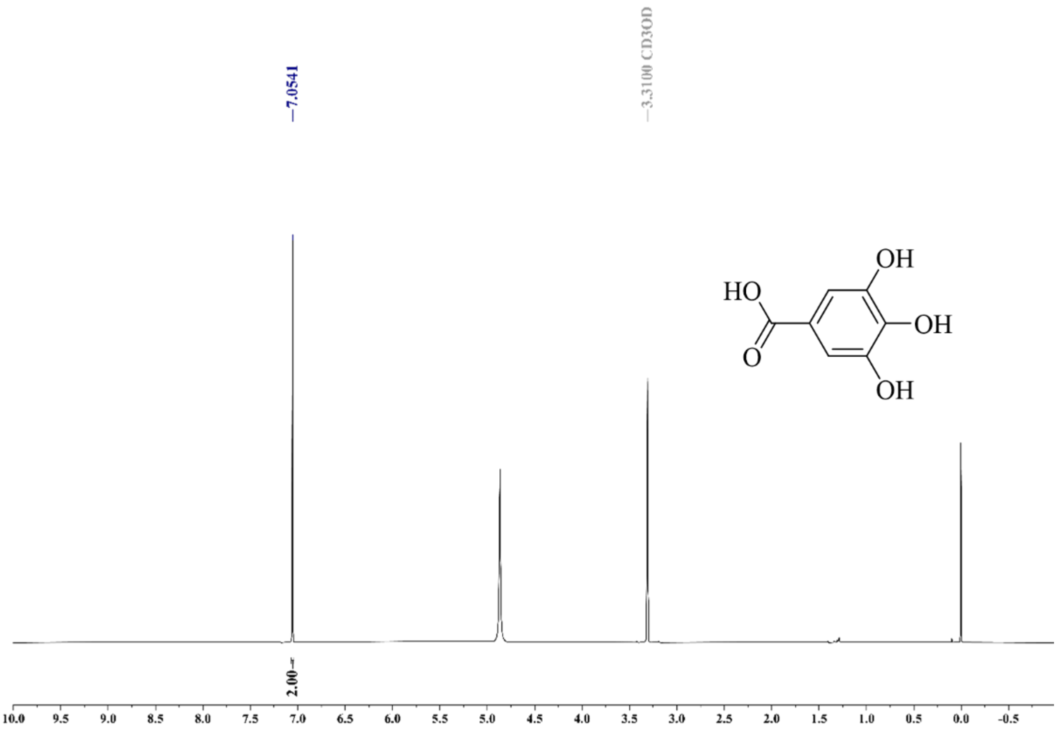

Fig. S3 ^{1}H NMR (600 MHz, CD_3OD) spectrum of **gallic acid**

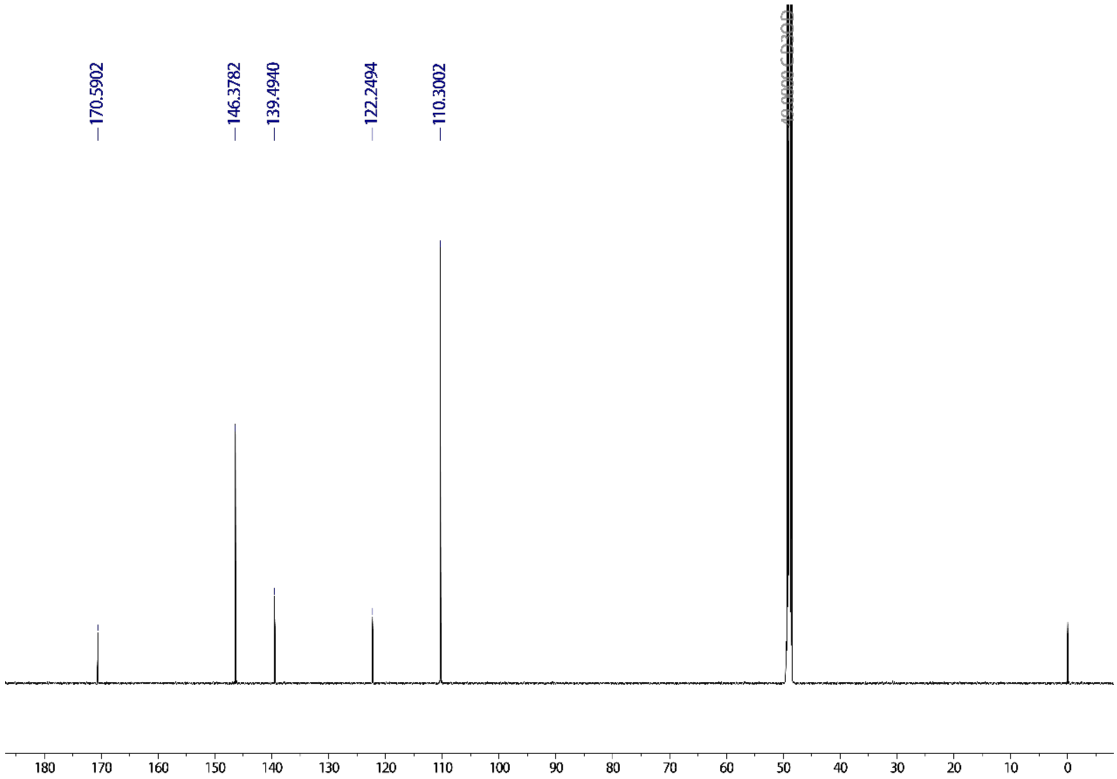

Fig. S4 ^{13}C NMR (150 MHz, CD_3OD) spectrum of **gallic acid**

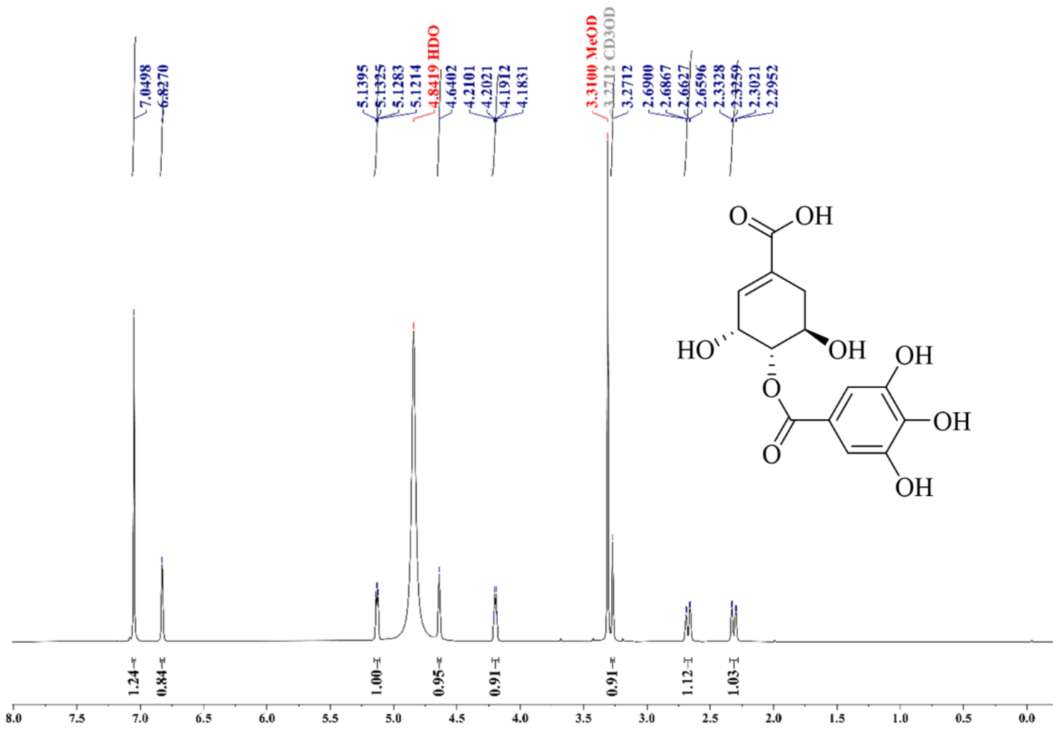

Fig. S5 ^{1}H NMR (600 MHz, CD_3OD) spectrum of **4-galloyl-(-)-shikimic acid**

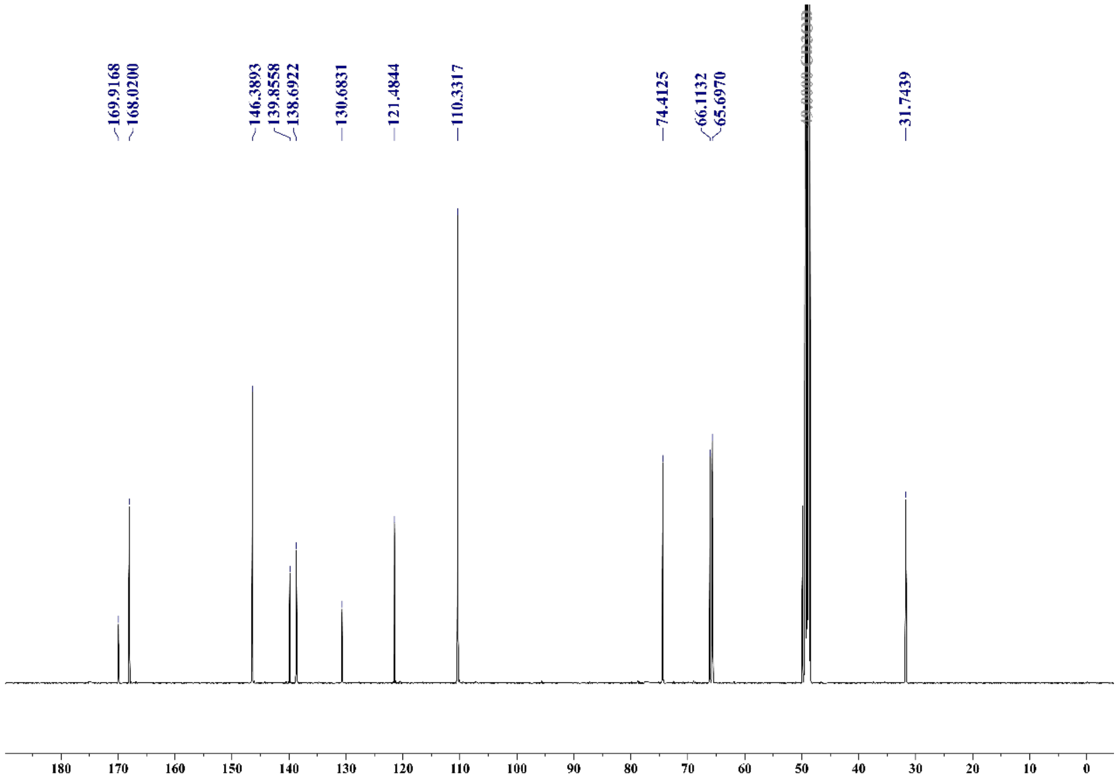

Fig. S6 ^{13}C NMR (150 MHz, CD_3OD) spectrum of **4-galloyl-(-)-shikimic acid**

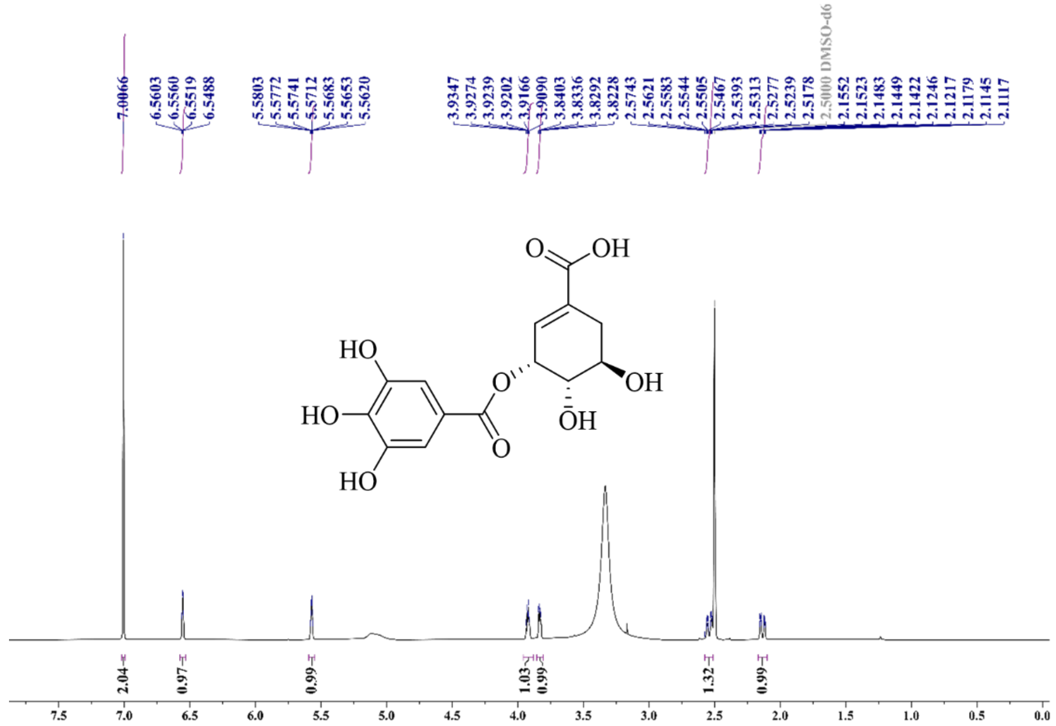

Fig. S7 ^{1}H NMR (600 MHz, DMSO-d_6) spectrum of **5-galloyl-(-)-shikimic acid**

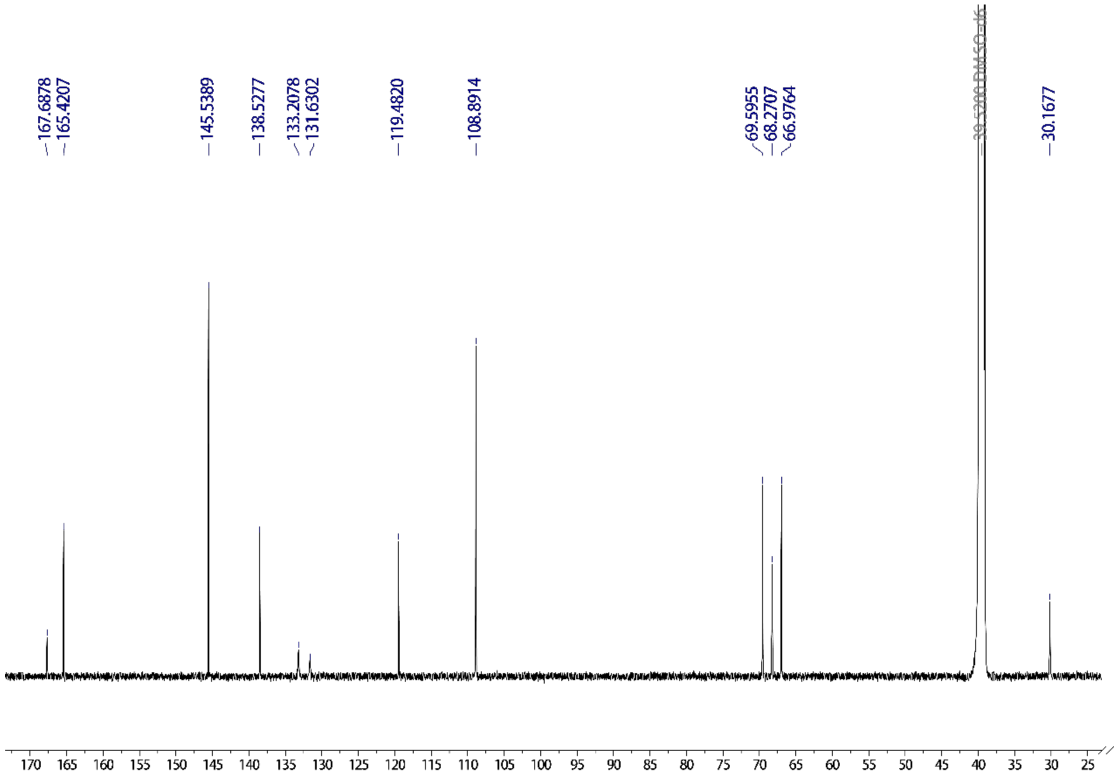

Fig. S8 ^{13}C NMR (150 MHz, DMSO-d_6) spectrum of **5-galloyl-(-)-shikimic acid**

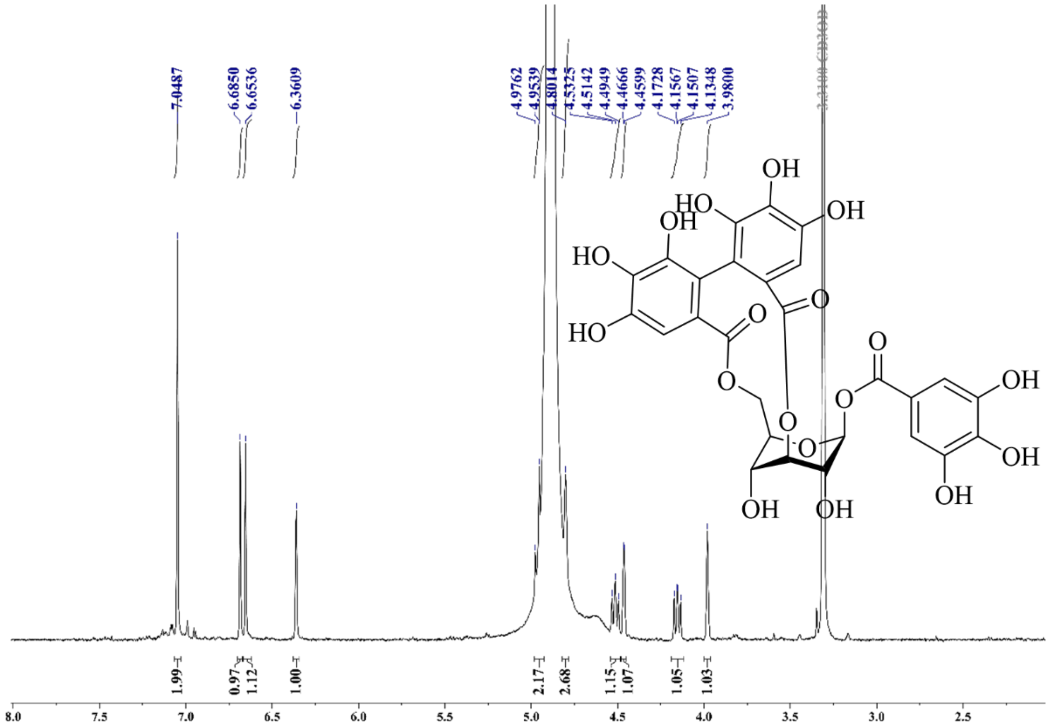

Fig. S9 ^{1}H NMR (600 MHz, CD_3OD) spectrum of **corilagin**

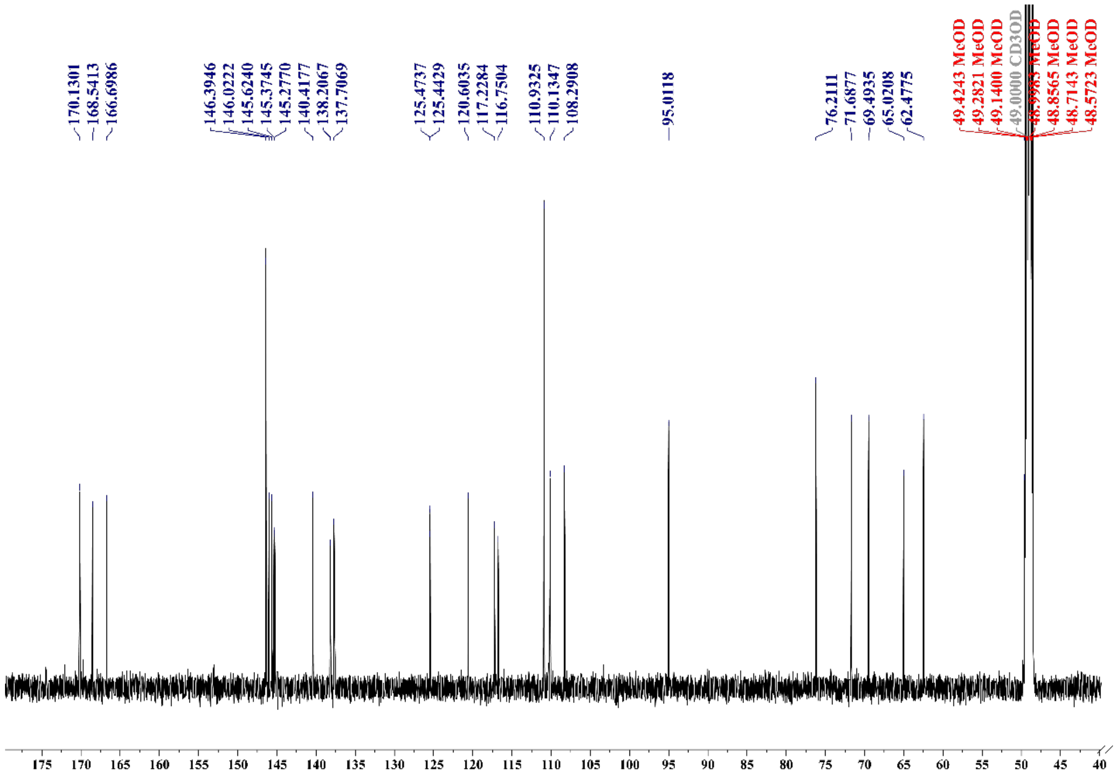

Fig. S10 ^{13}C NMR (150 MHz, CD_3OD) spectrum of **corilagin**

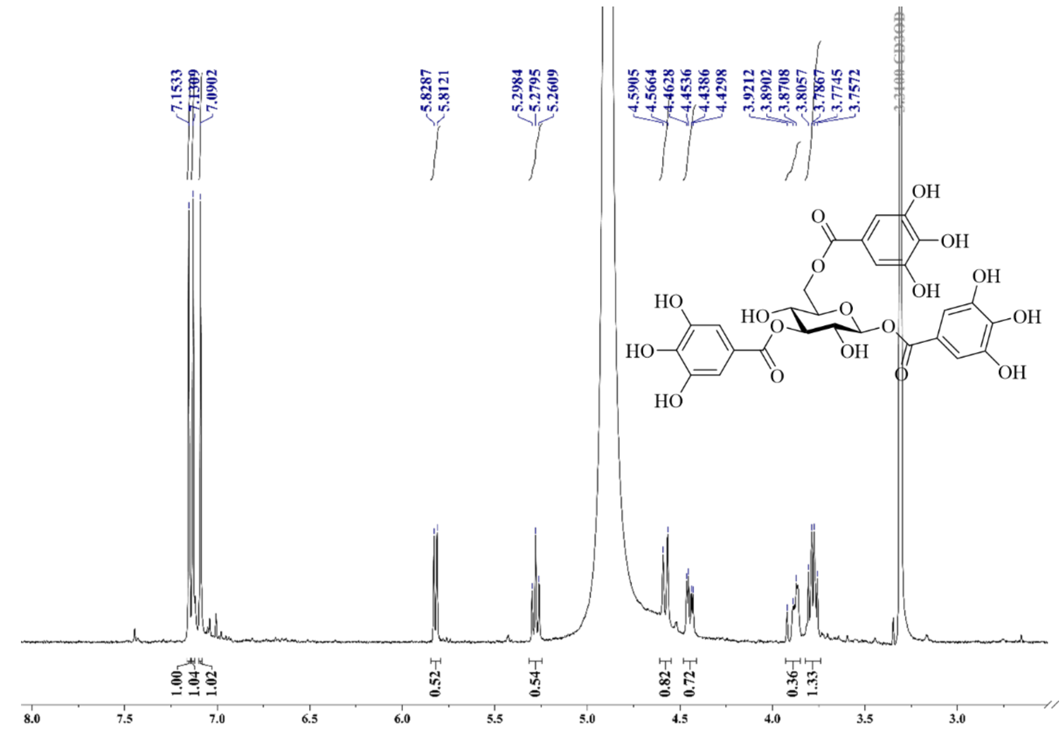

Fig. S11 ^{1}H NMR (600 MHz, CD_3OD) spectrum of **1,3,6-tri-*O*-*β*-D-galloylglucose**

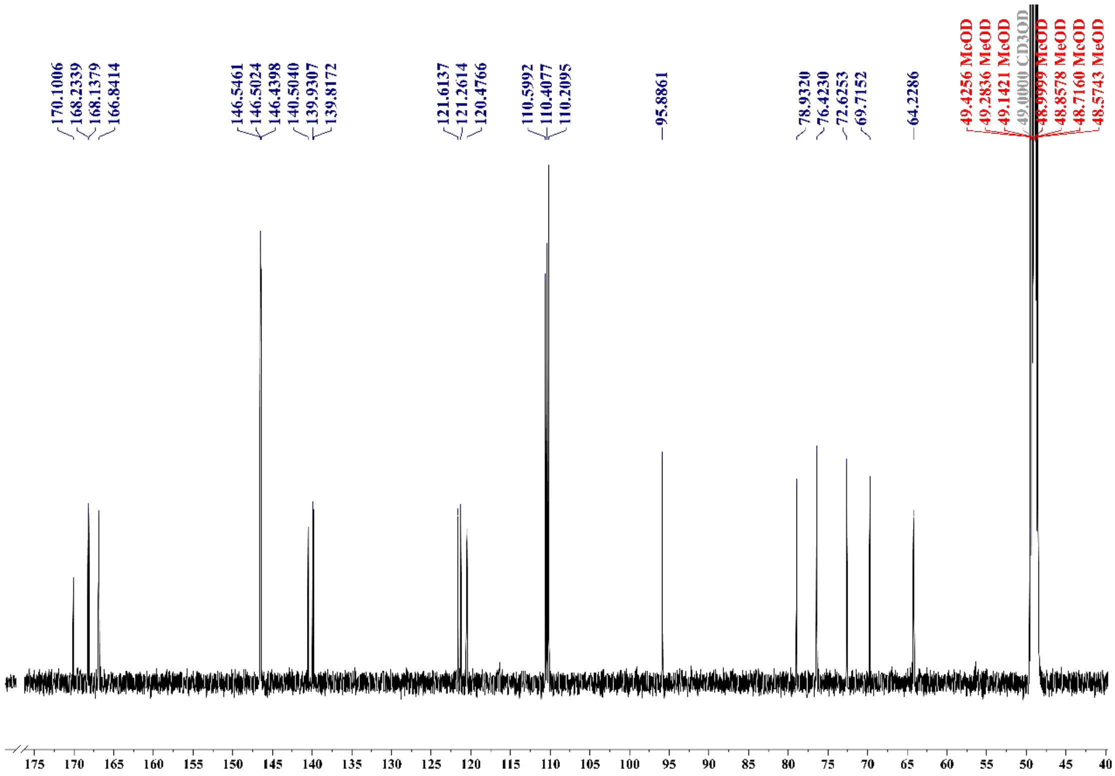

Fig. S12 ^{13}C NMR (150 MHz, CD_3OD) spectrum of **1,3,6-tri-*O*-*β*-D-galloylglucose**

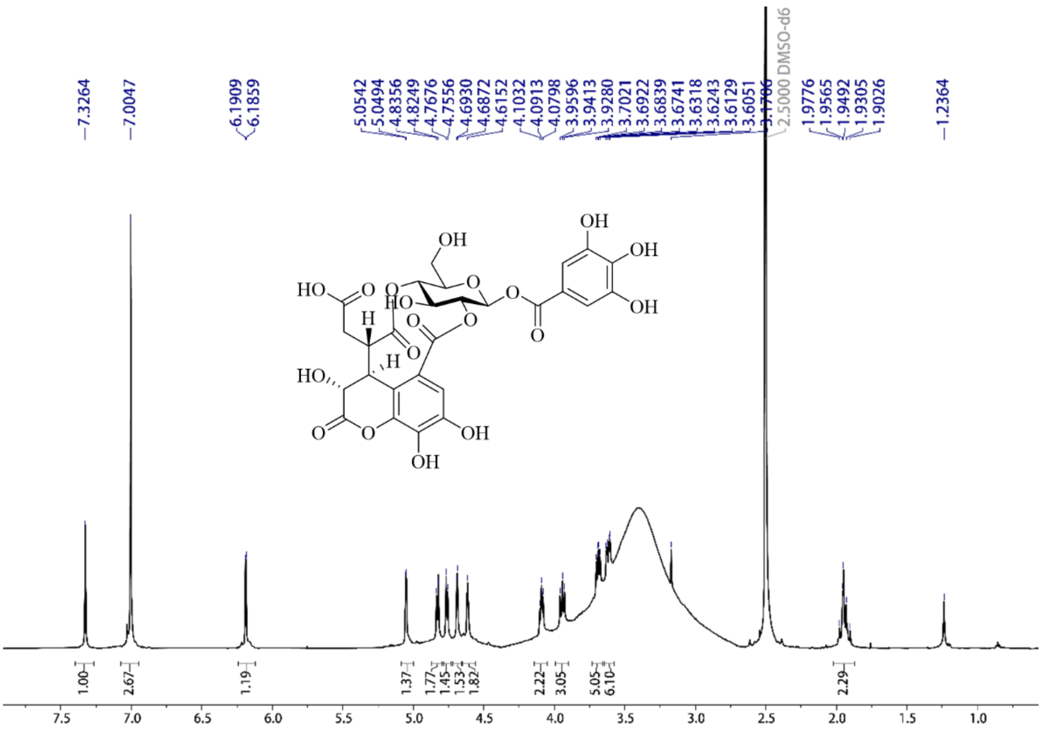

Fig. S13 ^{1}H NMR (600 MHz, DMSO-d_6) spectrum of **chebulanin**

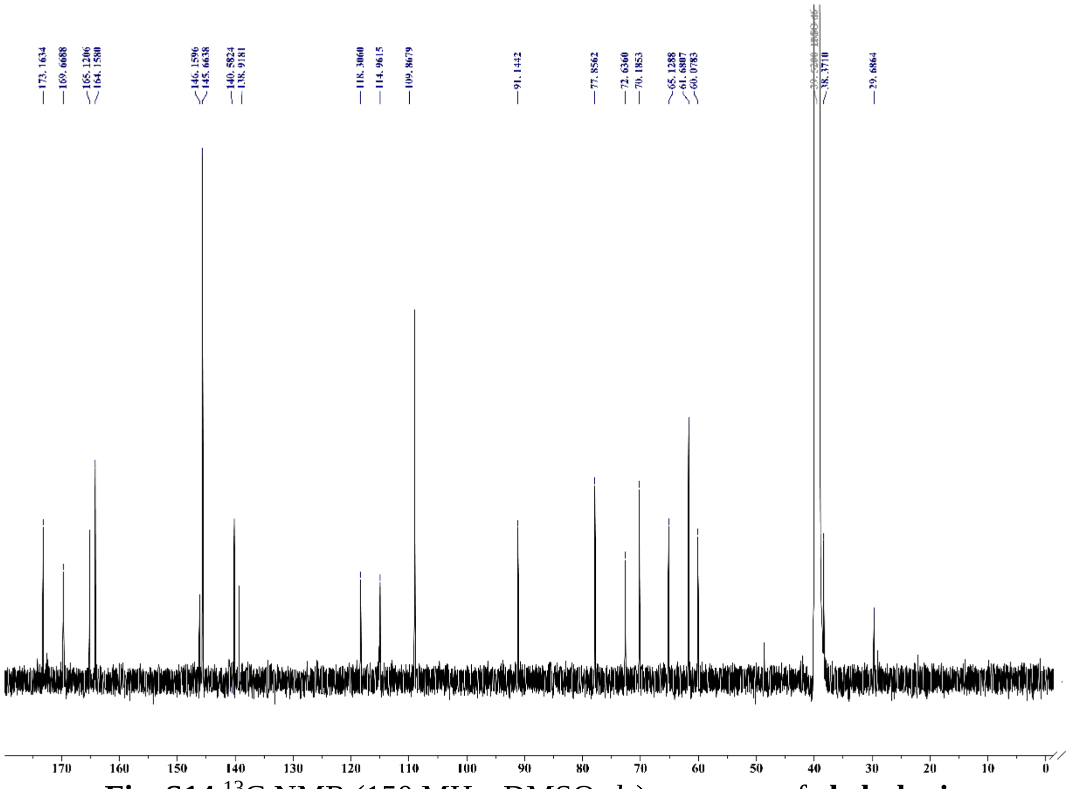

Fig. S14 ^{13}C NMR (150 MHz, DMSO-d_6) spectrum of **chebulanin**

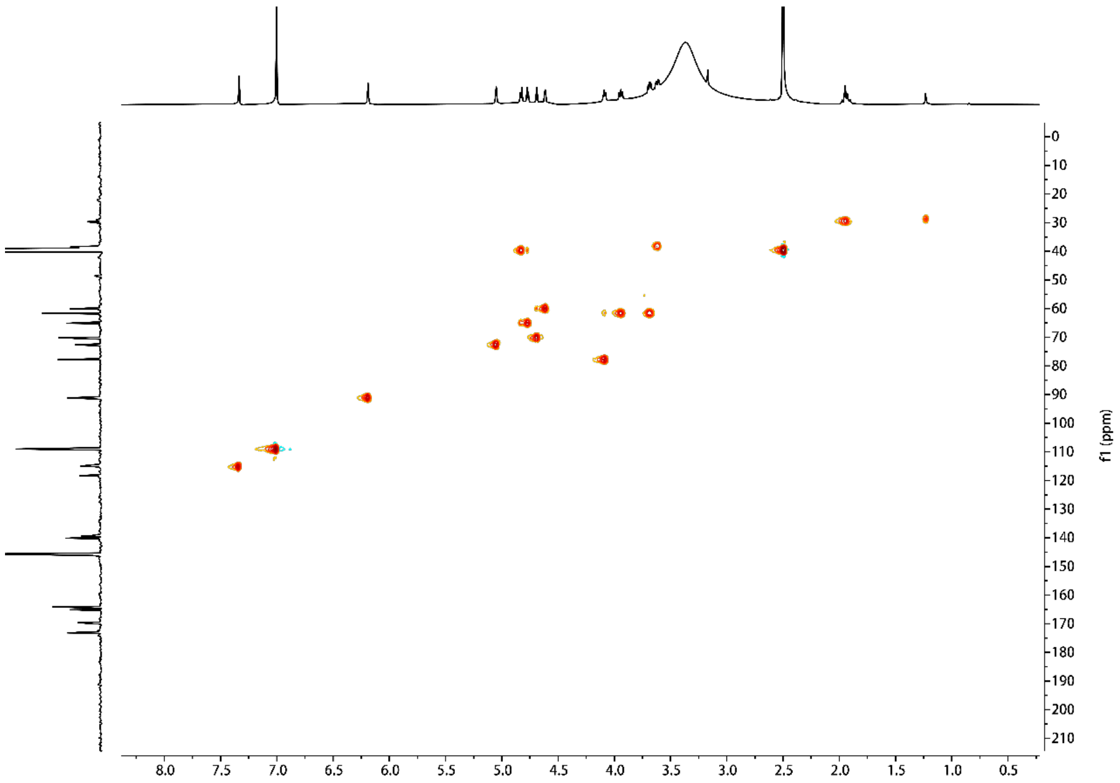

Fig. S15 HSQC spectrum of **chebulanin**

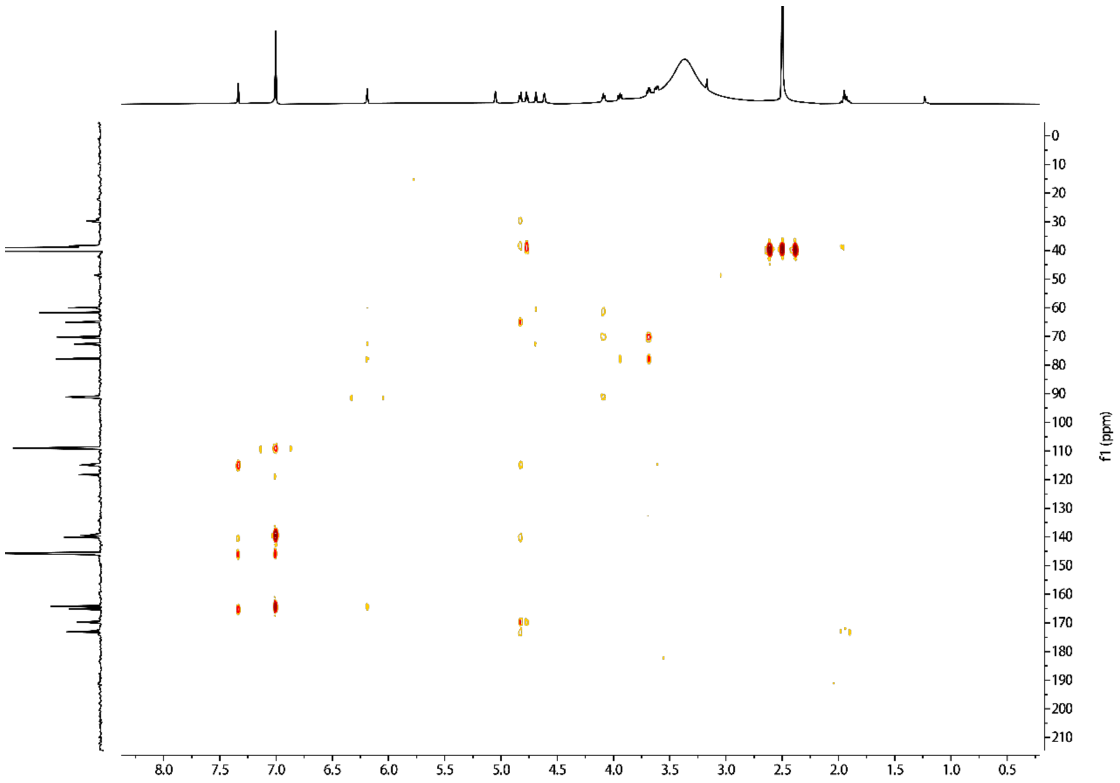

Fig. S16 HMBC spectrum of **chebulanin**

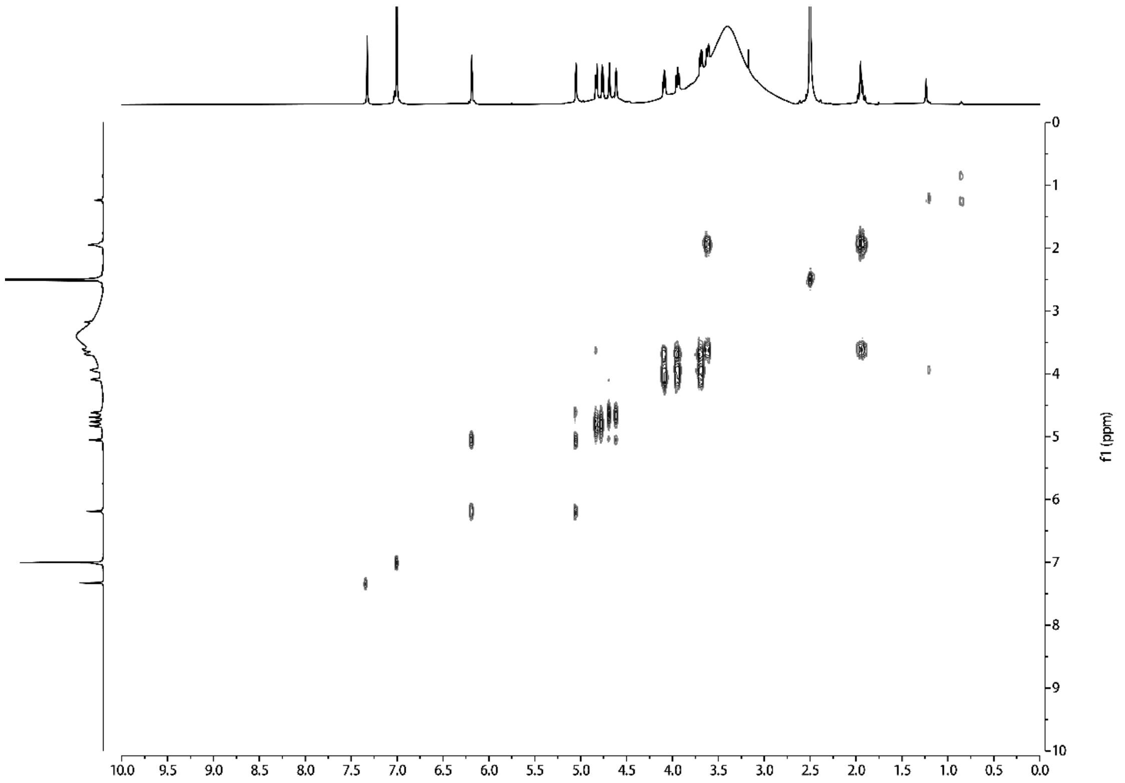

Fig. S17 ^{1}H-^{1}H COSY spectrum of **chebulanin**

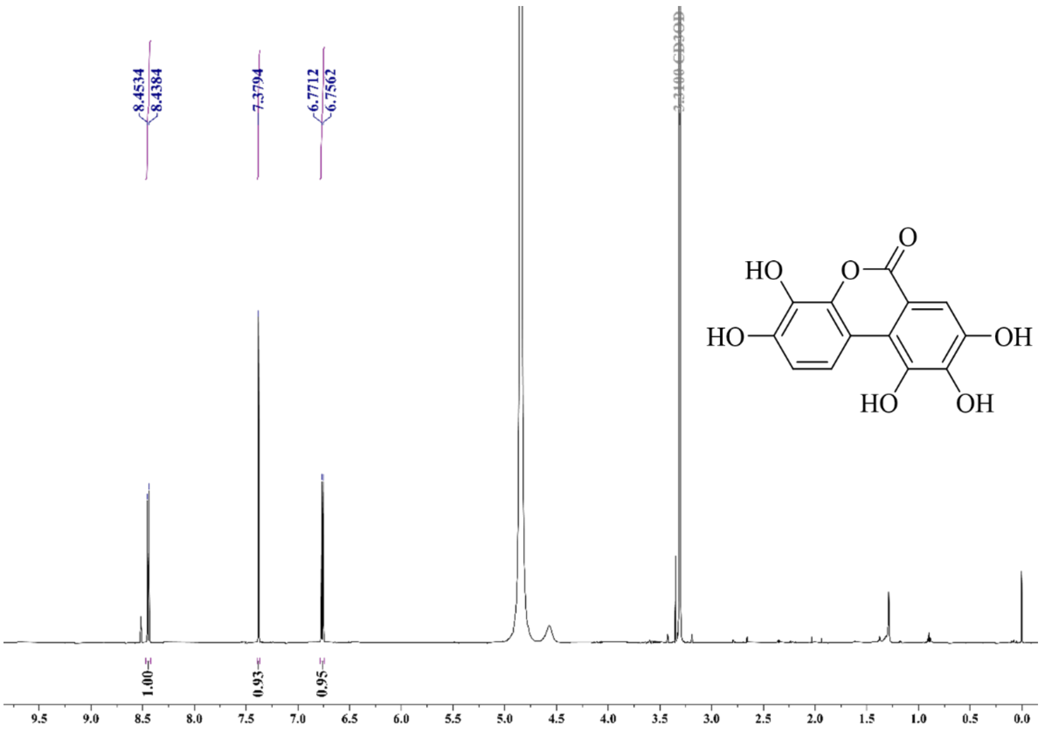

Fig. S18 ^{1}H NMR (600 MHz, CD3OD) spectrum of **urolithin M5**

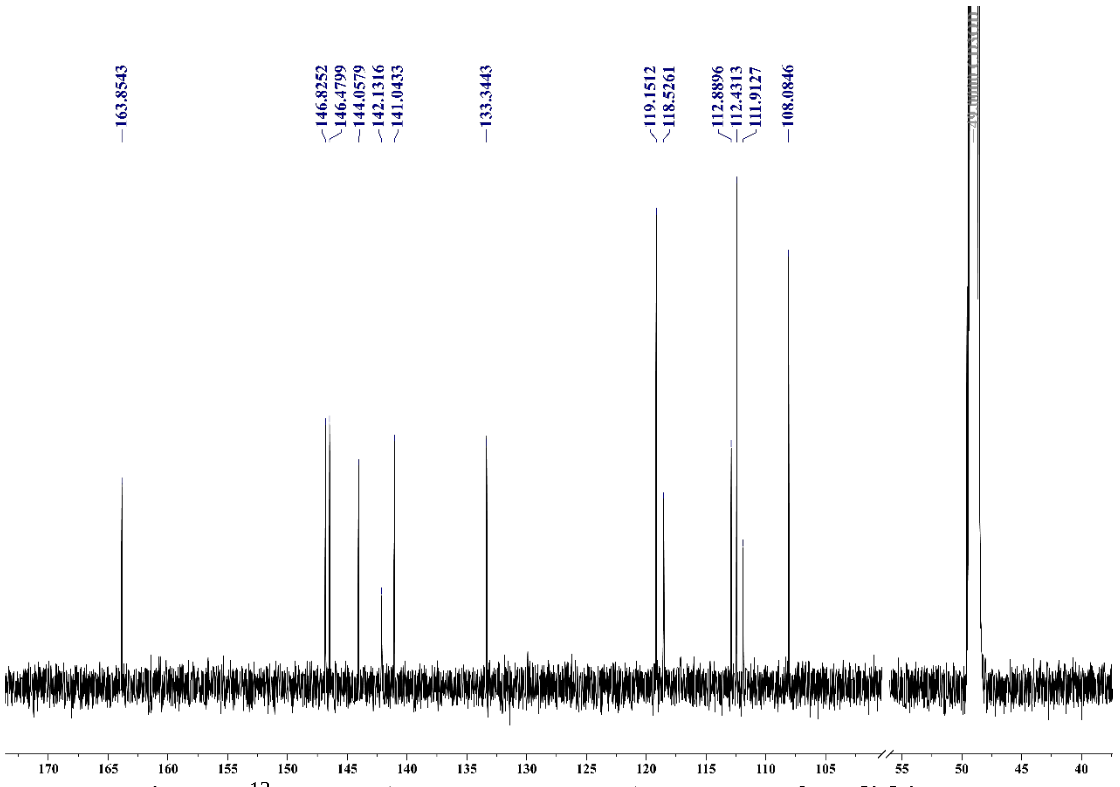

Fig. S19 ^{13}C NMR (150 MHz, CD3OD) spectrum of **urolithin M5**

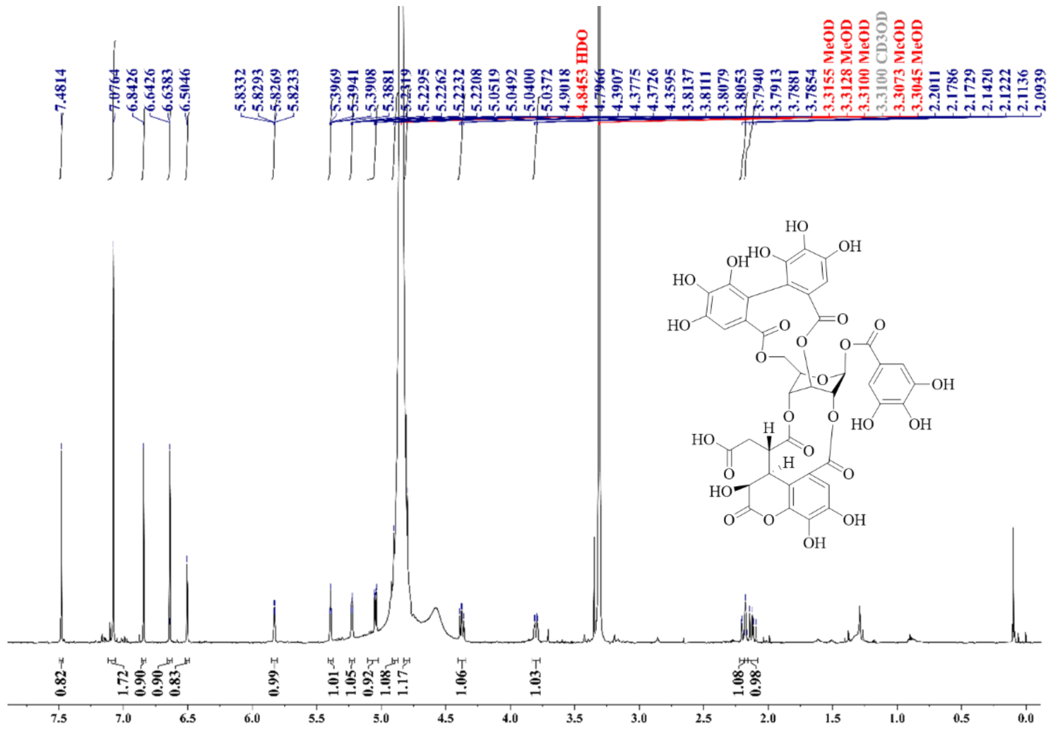

Fig. S20 ^{1}H NMR (600 MHz, CD3OD) spectrum of **chebulagic acid**

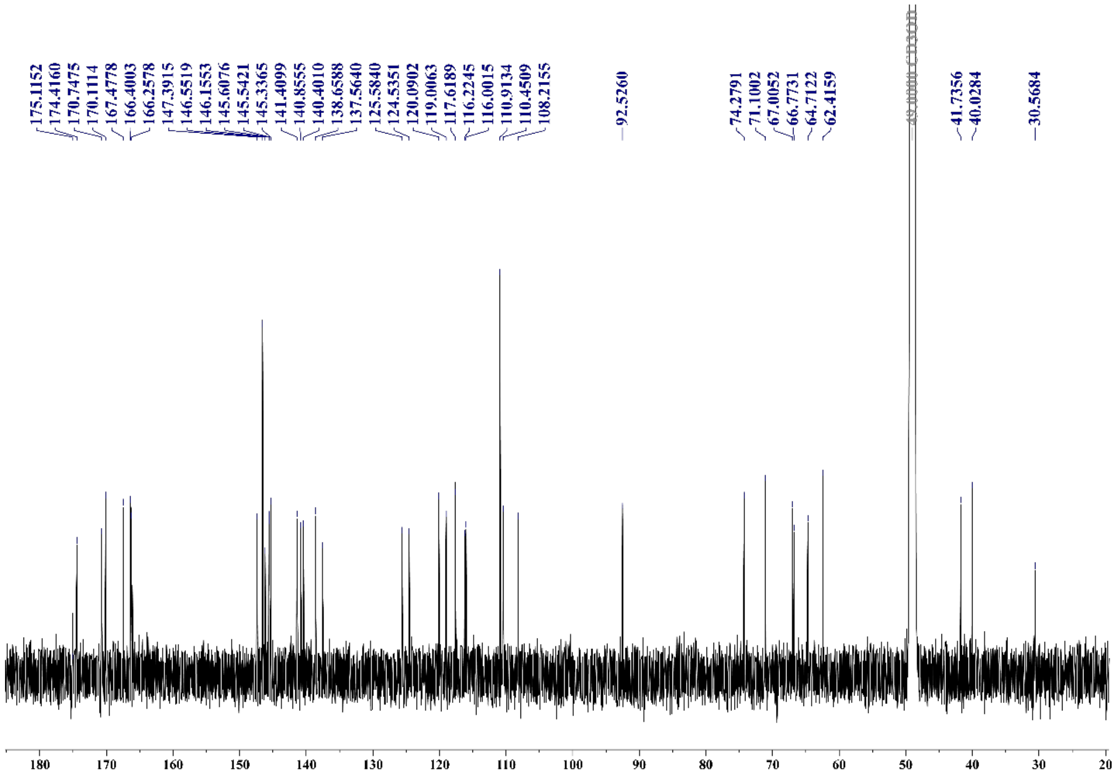

Fig. S21 ^{13}C NMR (150 MHz, CD_3OD) spectrum of **chebulagic acid**

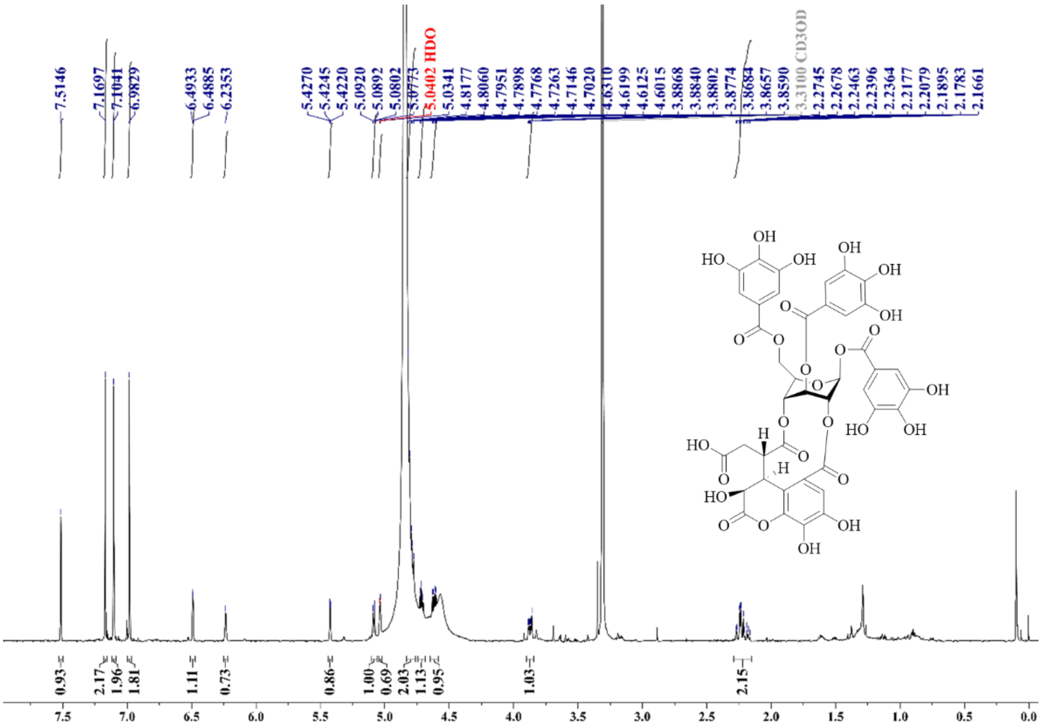

Fig. S22 ^{1}H NMR (600 MHz, CD_3OD) spectrum of **chebulinic acid**

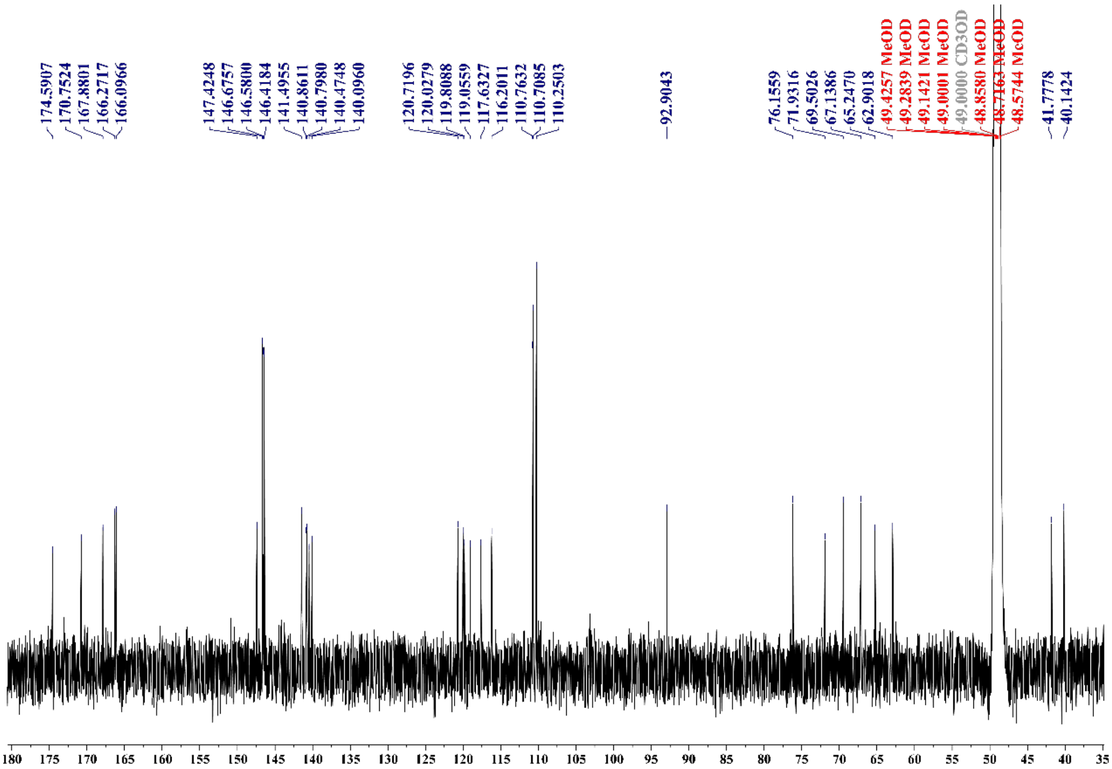

Fig. S23 ^{13}C NMR (150 MHz, CD_3OD) spectrum of **chebulinic acid**

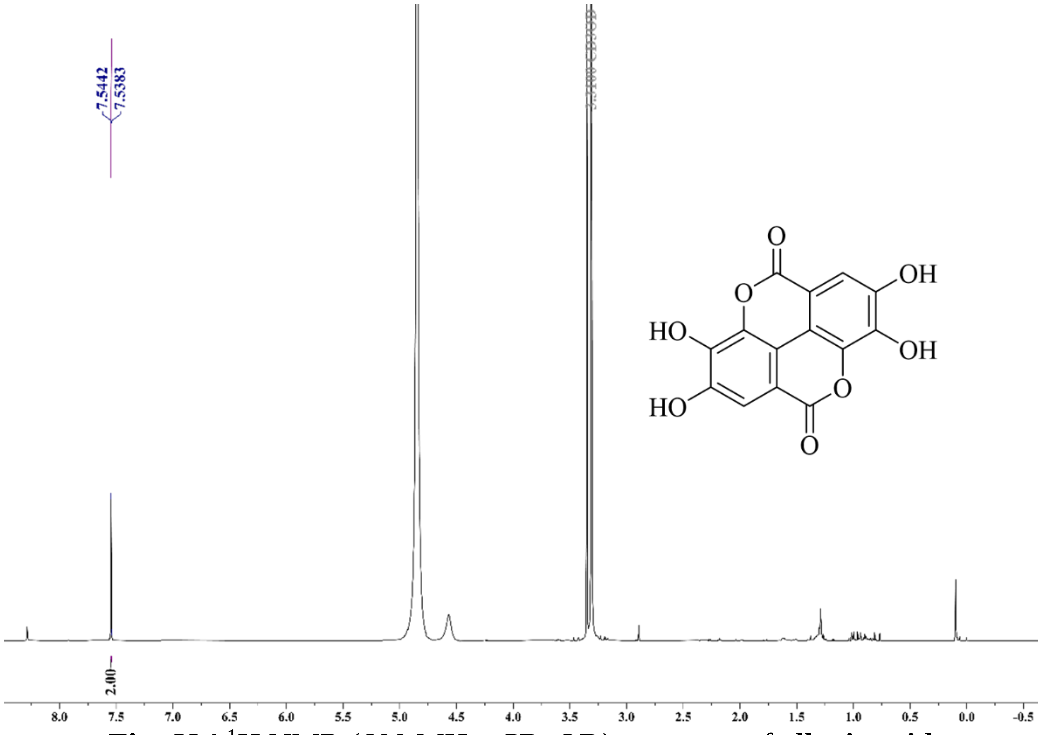

Fig. S24 ^{1}H NMR (600 MHz, CD_3OD) spectrum of **ellagic acid**

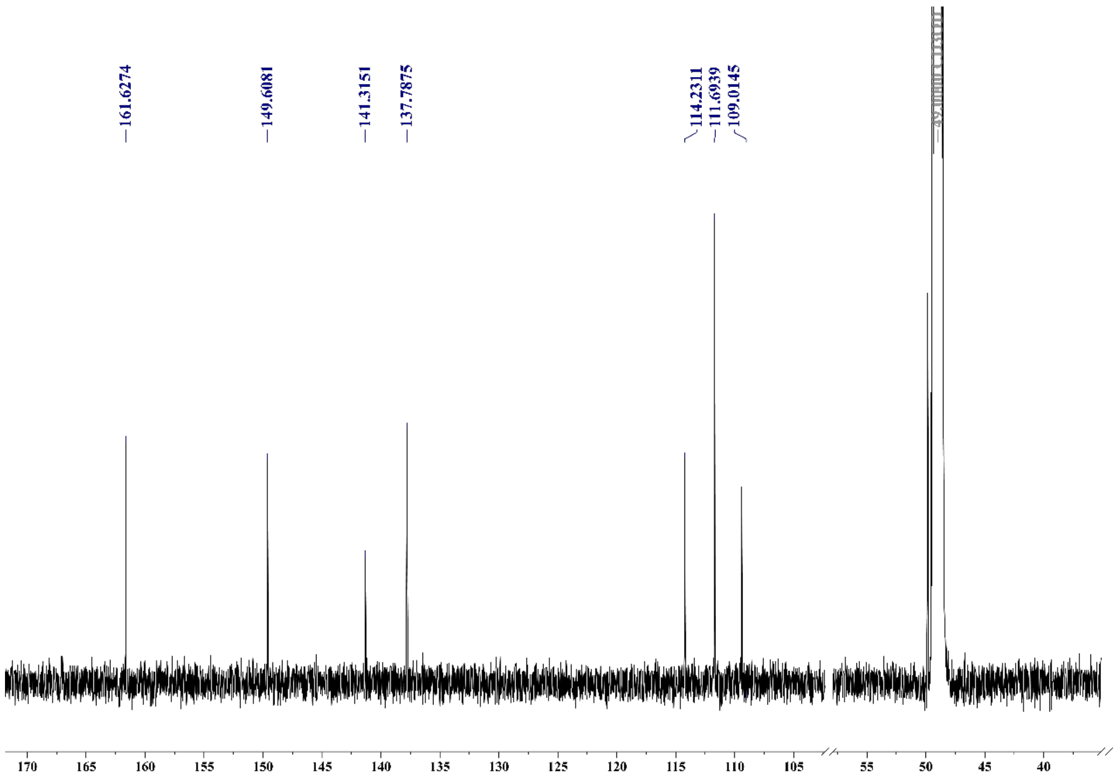

Fig. S25 ^{13}C NMR (150 MHz, CD_3OD) spectrum of **ellagic acid**

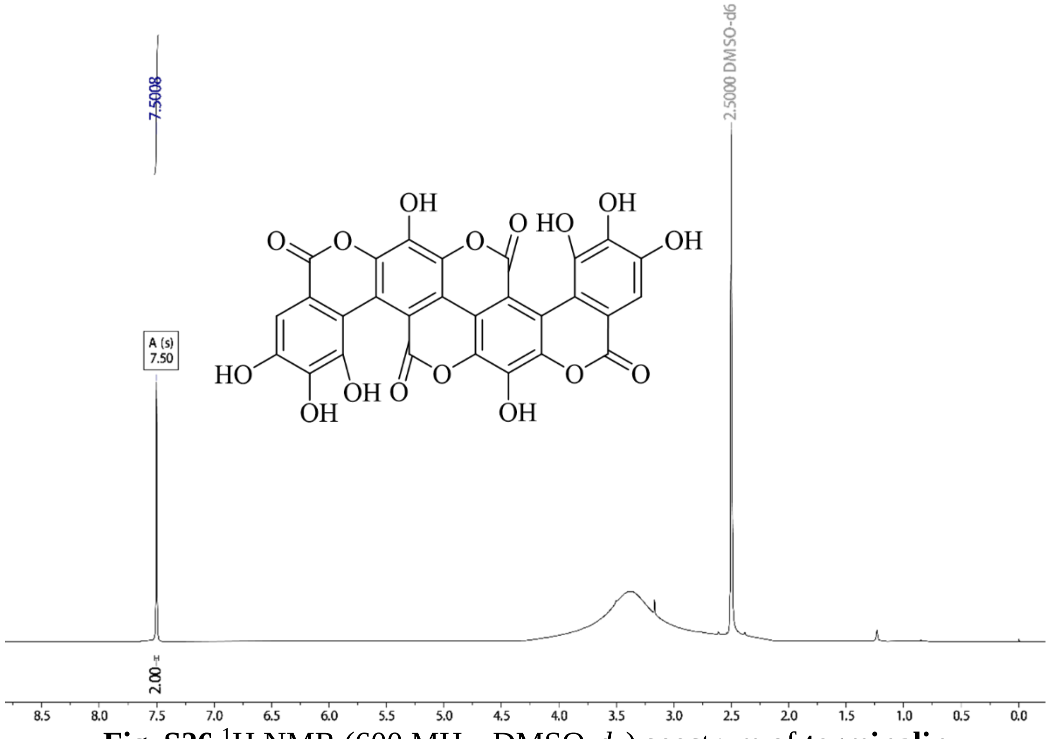

Fig. S26 ^{1}H NMR (600 MHz, DMSO-d_6) spectrum of **terminalin**

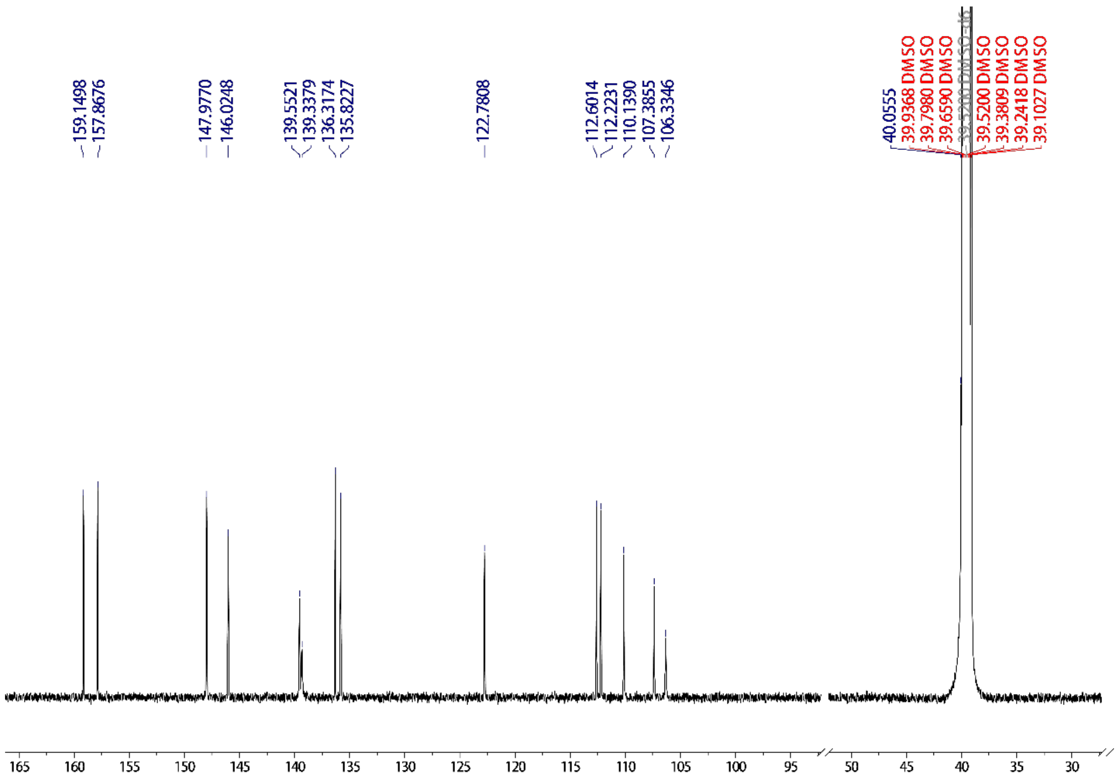

Fig. S27 ^{13}C NMR (150 MHz, DMSO-d_6) spectrum of **terminalin**

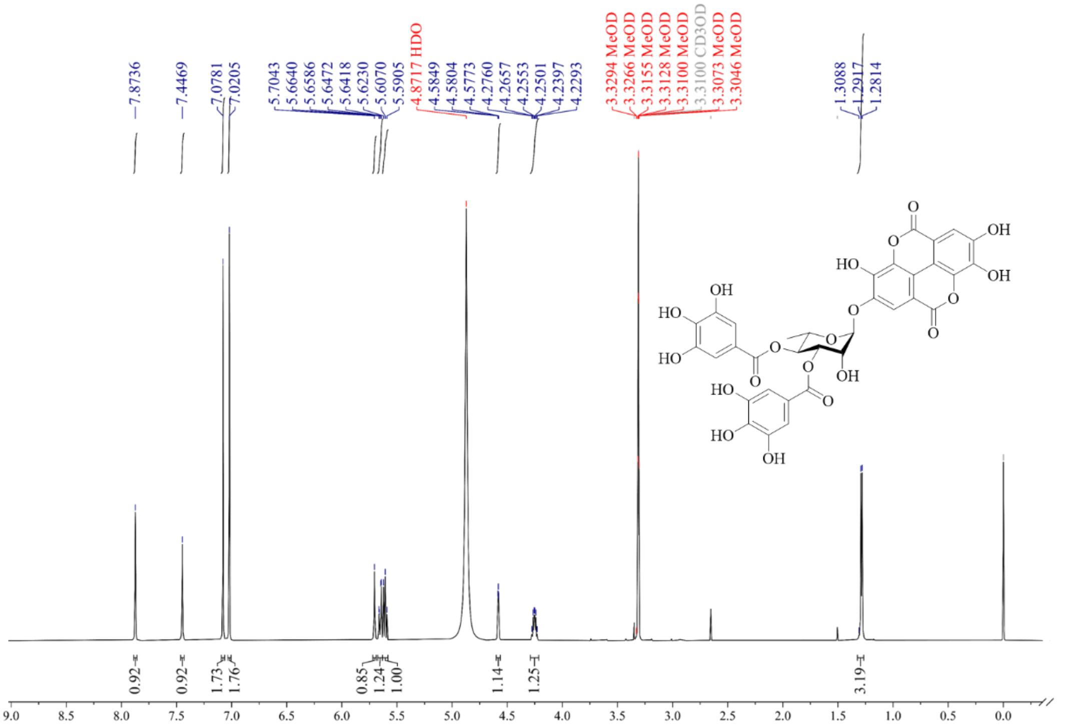

Fig. S28 ^{1}H NMR (600 MHz, CD_3OD) spectrum of **4-*O*-(3'',4''-di-*O*-galloyl-*α*-L-rhamnosyl)ellagic acid**

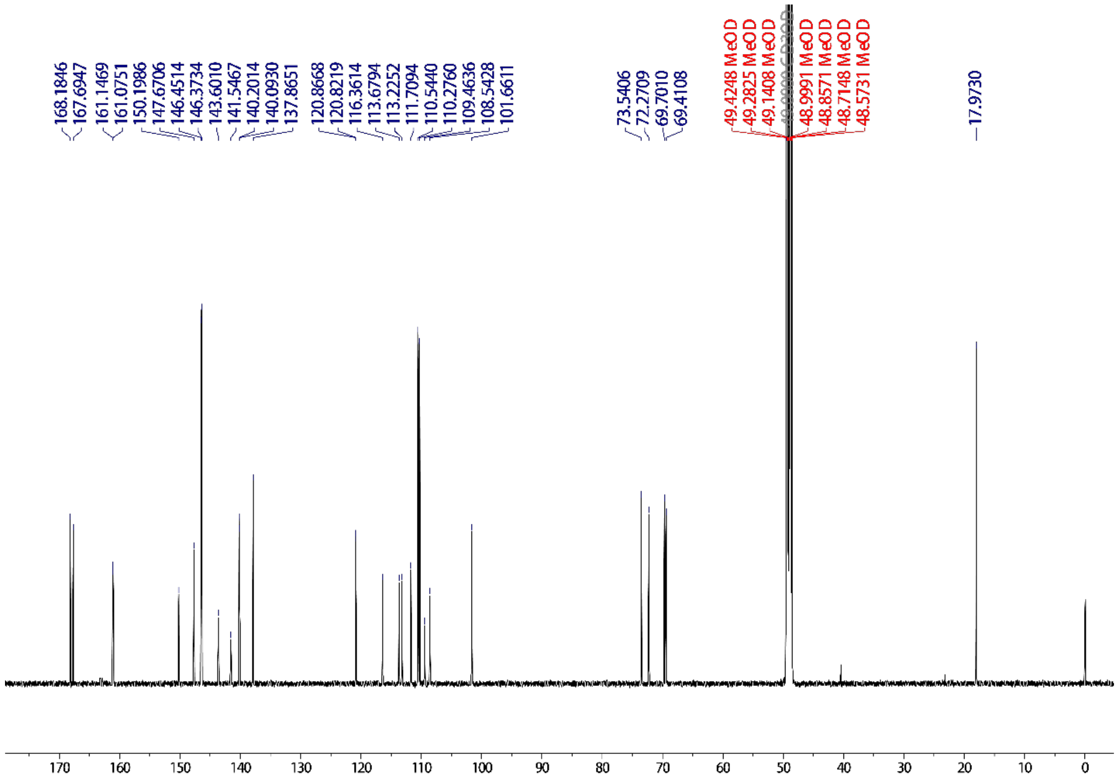

Fig. S29 ^{13}C NMR (150 MHz, CD_3OD) spectrum of **4-*O*-(3'',4''-di-*O*-galloyl-*α*-L-rhamnosyl)ellagic acid**

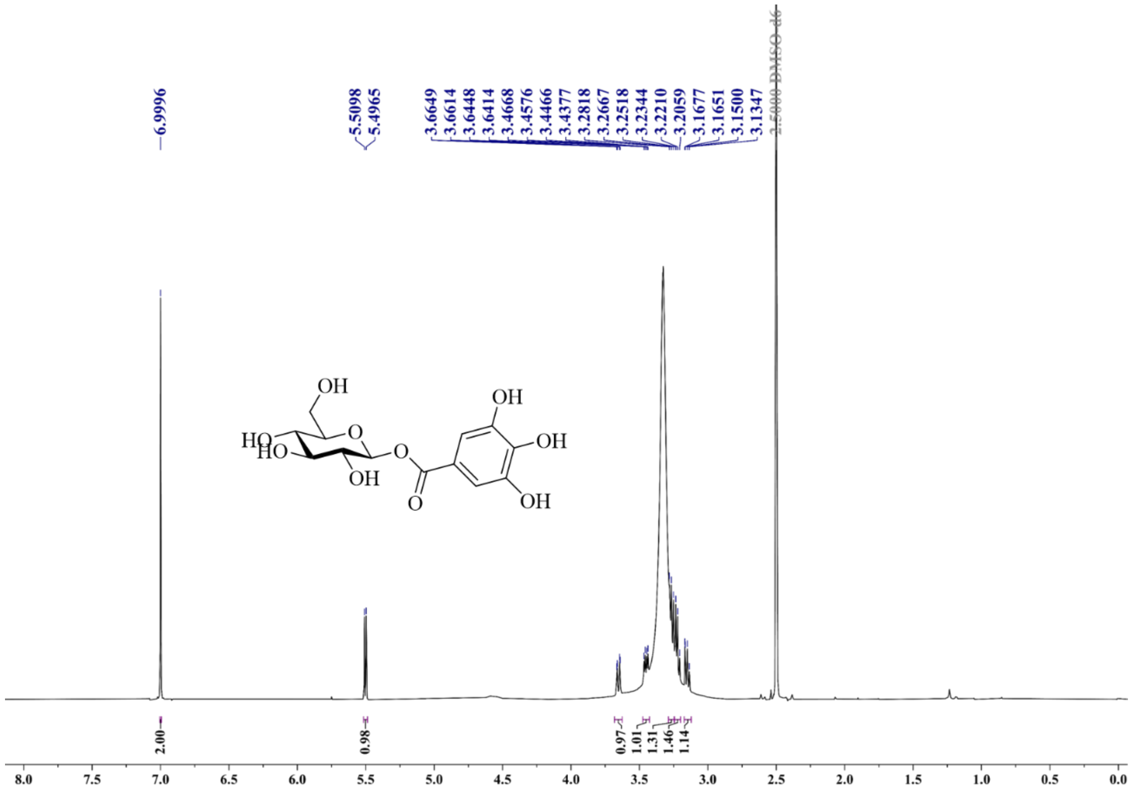

Fig. S30 ^{1}H NMR (600 MHz, DMSO-d_6) spectrum of **1-*O*-galloyl-*β*-D-glucose**

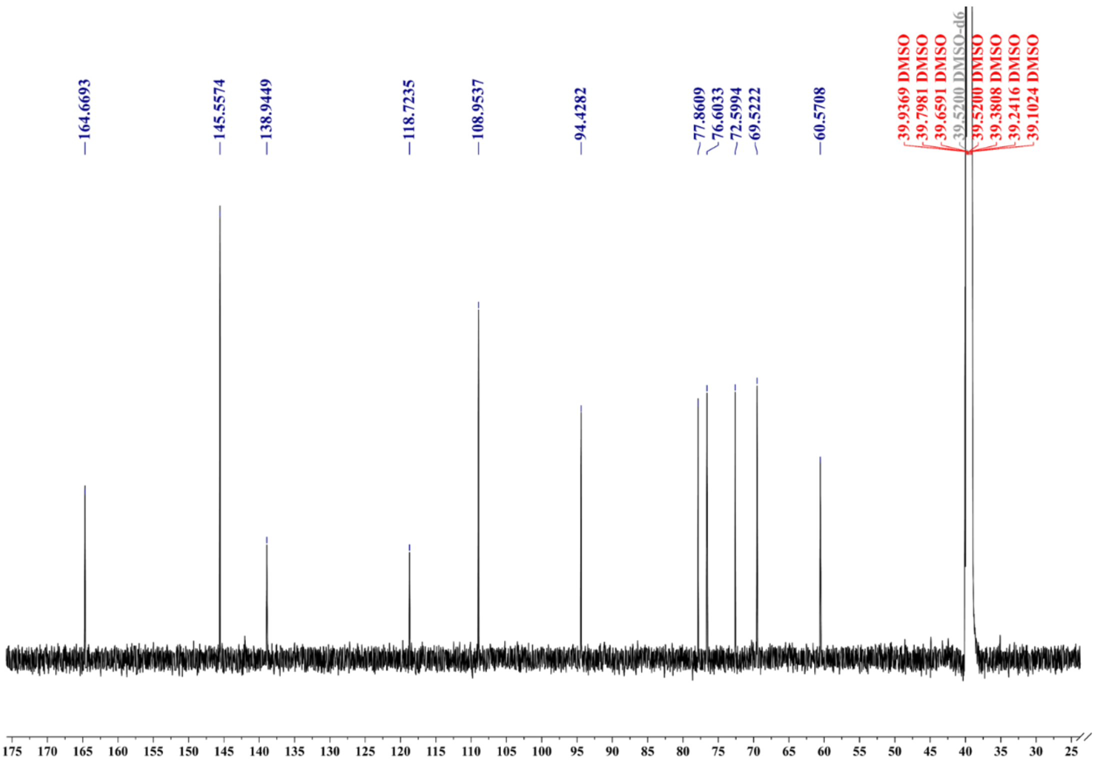

Fig. S31 ^{13}C NMR (150 MHz, DMSO-d_6) spectrum of **1-*O*-galloyl-*β*-D-glucose**

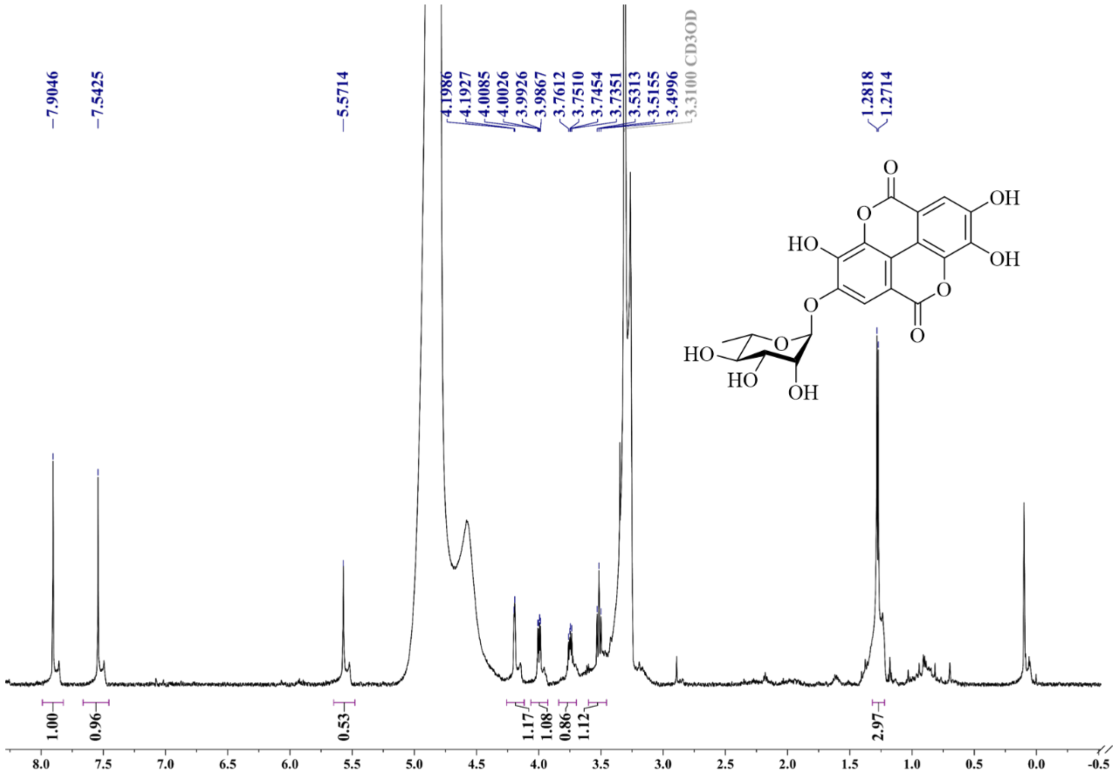

Fig. S32 ^{1}H NMR (600 MHz, CD_3OD) spectrum of **eschweilenol C**

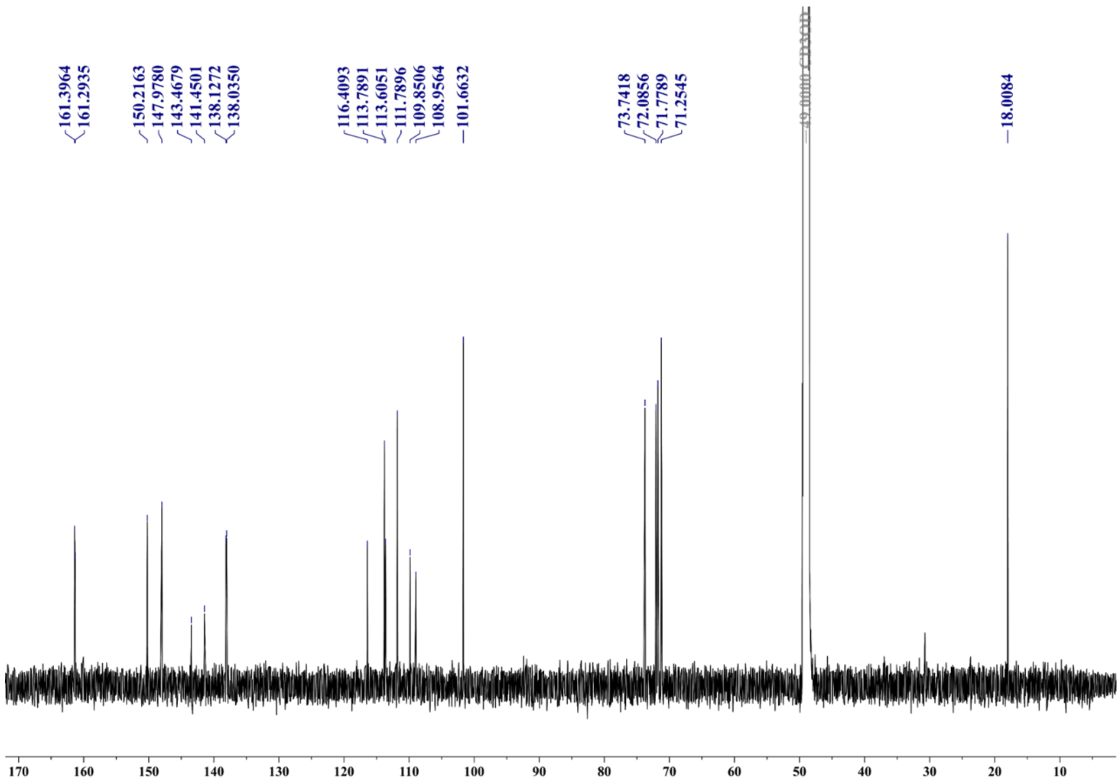

Fig. S33 ^{13}C NMR (150 MHz, CD_3OD) spectrum of **eschweilenol C**

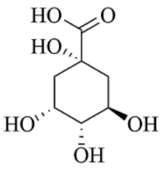

1

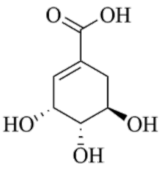

2

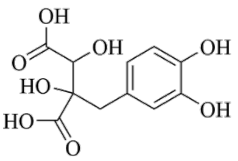

17

phenolcarboxylic acids

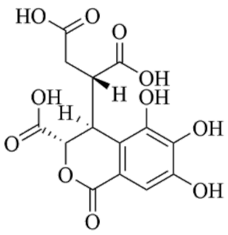

3

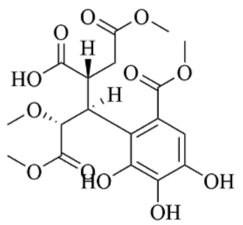

10

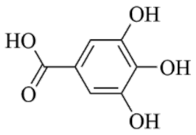

12

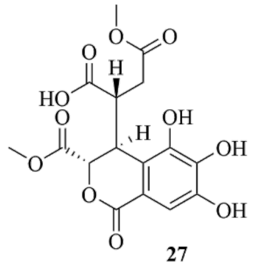

27

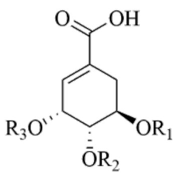

30: R_2 = G, R_1 = R_3 = H
33: R_3 = G, R_1 = R_2 = H
34: R_1 = G, R_2 = R_3 = H
58: R_2 = H, R_1 = R_3 = G
67: R_1 = H, R_2 = R_3 = G
74: R_3 = H, R_1 = R_2 = G
94: R_1 = R_2 = R_3 = G

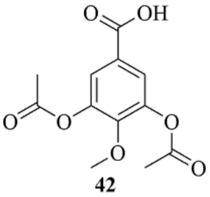

42

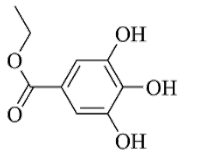

45

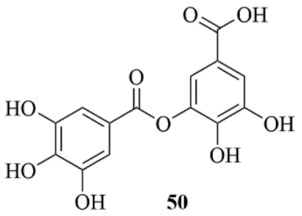

50

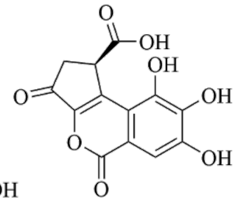

64

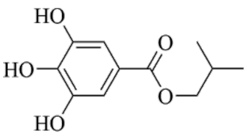

82

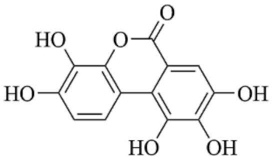

86

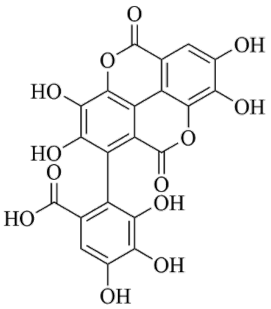

52

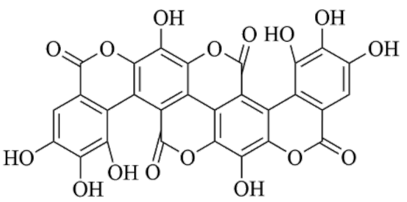

112

Fig. S34 Chemical structures of the identified 122 compounds.

gallotannins

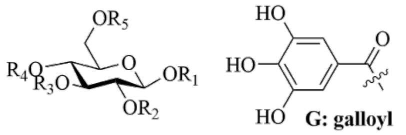

G: galloyl

4: R_5 = G, $R_1 = R_2 = R_3 = R_4$ = H
7: R_4 = G, $R_1 = R_2 = R_3 = R_5$ = H
15: R_1 = G, $R_2 = R_3 = R_4 = R_5$ = H
18: R_3 = G, $R_1 = R_2 = R_4 = R_5$ = H
22: R_2 = G, $R_1 = R_3 = R_4 = R_5$ = H
28: $R_2 = R_4$ = G, $R_1 = R_3 = R_5$ = H
35: $R_2 = R_3$ = G, $R_1 = R_4 = R_5$ = H
38: $R_1 = R_2$ = G, $R_3 = R_4 = R_5$ = H
41: $R_1 = R_3$ = G, $R_2 = R_4 = R_5$ = H
46: $R_1 = R_5$ = G, $R_2 = R_3 = R_4$ = H
48: $R_4 = R_5$ = G, $R_1 = R_2 = R_3$ = H
49: $R_3 = R_4$ = G, $R_1 = R_2 = R_5$ = H
53: $R_3 = R_5$ = G, $R_1 = R_2 = R_4$ = H
68: $R_1 = R_3 = R_5$ = G, $R_2 = R_4$ = H
70: $R_1 = R_4 = R_5$ = G, $R_2 = R_3$ = H
72: $R_1 = R_2 = R_5$ = G, $R_3 = R_4$ = H
75: $R_1 = R_2 = R_4 = R_5$ = G, R_3 = H
89: $R_2 = R_3 = R_4 = R_5$ = G, R_1 = H
92: $R_1 = R_2 = R_3 = R_5$ = G, R_4 = H
97: $R_1 = R_2 = R_3 = R_4 = R_5$ = G

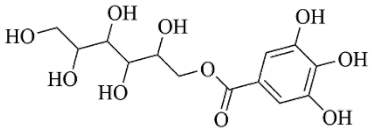

19

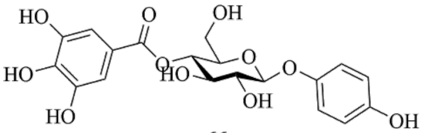

66

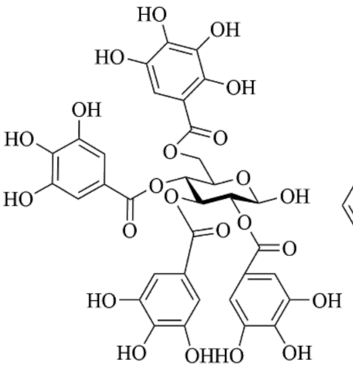

100

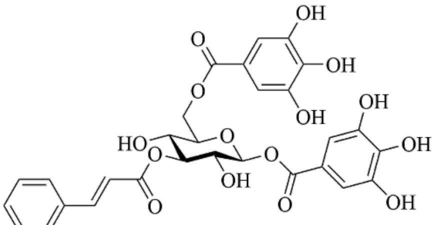

105

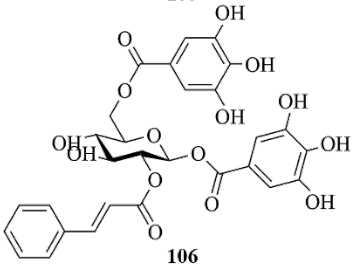

106

ellagitannins

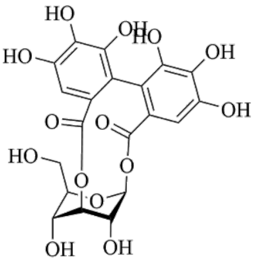

6

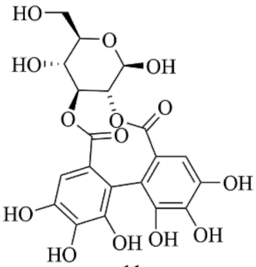

11

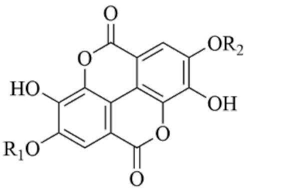

29: R_1 = Glc, R_2 = H
101: R_1 = GlcA, R_2 = H
108: R_1 = GlcA, $R_2 = CH_3$
110: $R_1 = R_2$ = H
111: R_1 = Rha, R_2 = H
116: R_1 = Rha, $R_2 = CH_3$

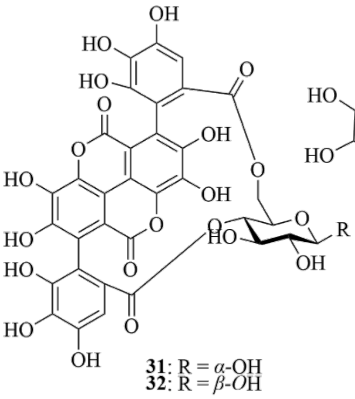

31: R = α-OH
32: R = β-OH

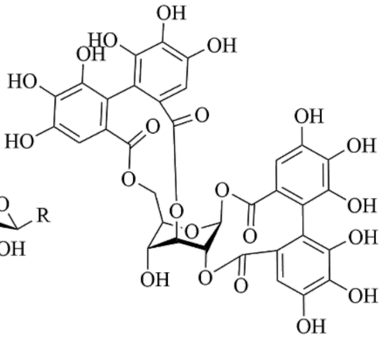

36

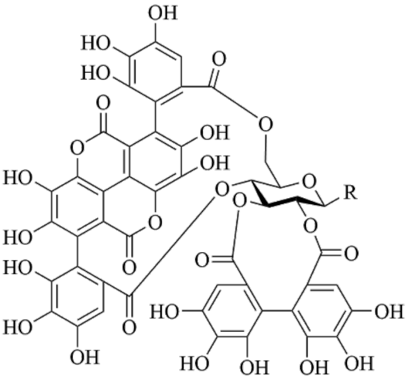

39: R = α-OH
47: R = β-OH

Fig. S34 Chemical structures of the identified 122 compounds (Continued)

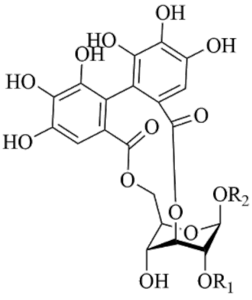

37: R_1 = G, R_2 = H
56: R_1 = H, R_2 = G

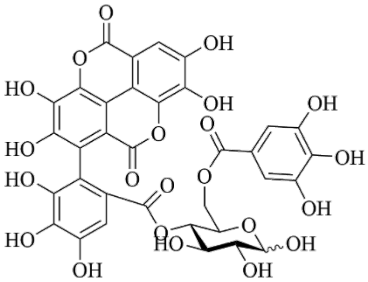

43

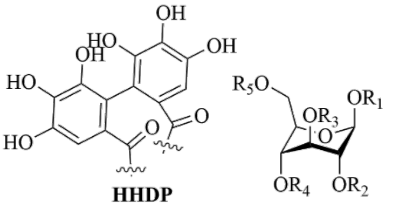

44: $R_1 = R_4$ = G, R_2 = H, $R_3 = R_5$ = HHDP
57: $R_1 = R_2$ = G, R_4 = H, $R_3 = R_5$ = HHDP
61: $R_2 = R_4$ = G, R_1 = H, $R_3 = R_5$ = HHDP
65: $R_2 = R_4$ = G, R_5 = H, $R_1 = R_3$ = HHDP

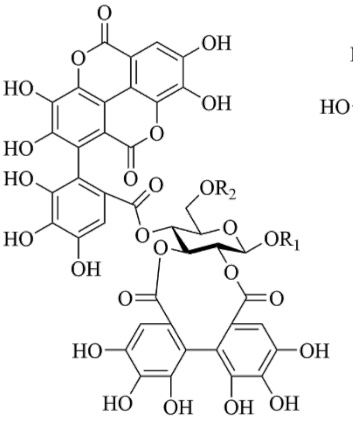

60: R_1 = G, R_2 = H
71: R_1 = H, R_2 = G

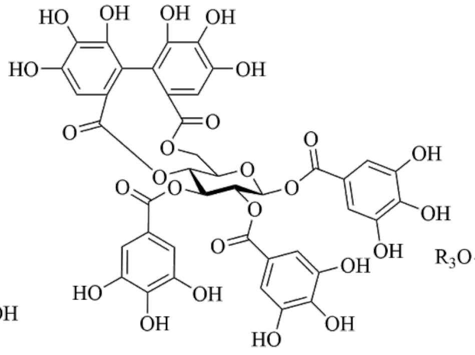

83

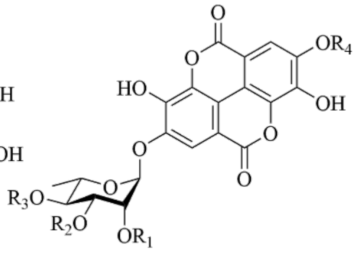

118: $R_1 = R_2 = R_4$ = H, R_3 = G
121: $R_1 = R_4$ = H, $R_2 = R_3$ = G
122: $R_1 = R_3 = R_4$ = G, R_2 = H

chebulic ellagitannins

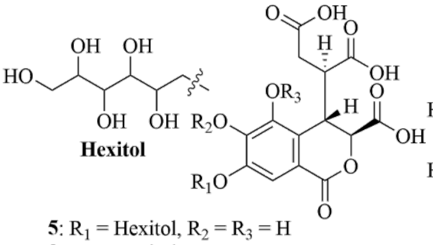

5: R_1 = Hexitol, $R_2 = R_3$ = H
8: R_2 = Hexitol, $R_1 = R_3$ = H
13: R_3 = Hexitol, $R_1 = R_2$ = H

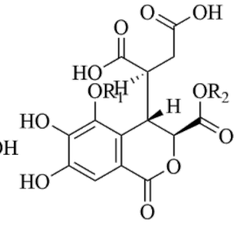

9: R_1 = Glc, R_2 = H
14: R_1 = H, R_2 = Glc

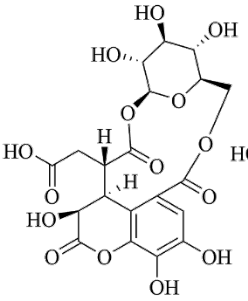

16

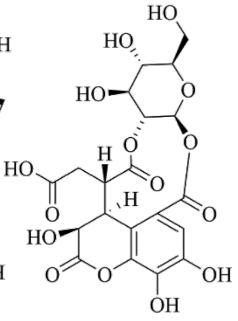

21

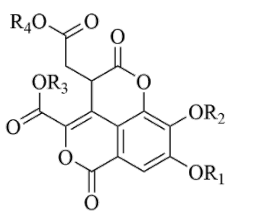

20: $R_1 = R_2$ = H, R_3 = Glc, $R_4 = CH_3$
23: $R_1 = R_4$ = H, R_3 = Glc, $R_2 = CH_3$
24: $R_2 = R_4$ = H, R_3 = Glc, $R_1 = CH_3$
25: $R_2 = R_3$ = H, R_4 = Glc, $R_1 = CH_3$
26: $R_1 = R_3$ = H, R_4 = Glc, $R_2 = CH_3$

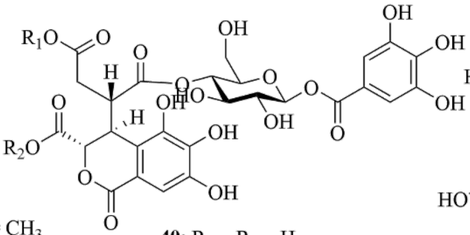

40: $R_1 = R_2$ = H
51: $R_1 = CH_3$, R_2= H
62: R_1 = H, $R_2 = CH_3$

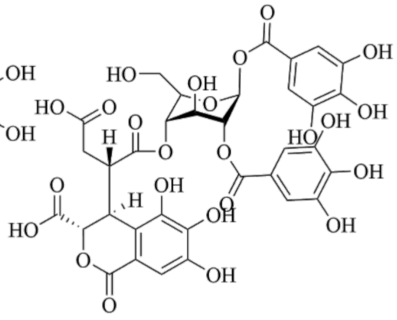

54

Fig. S34 Chemical structures of the identified 122 compounds (Continued)

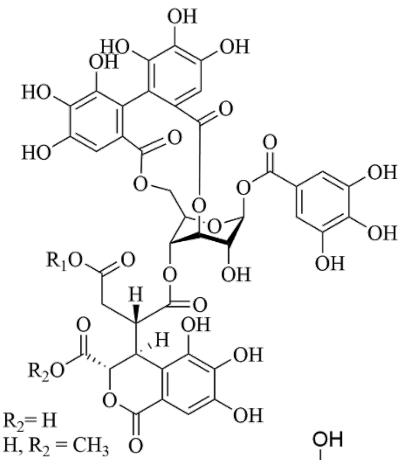

59: $R_1 = R_2 = H$
73: $R_1 = H, R_2 = CH_3$
79: $R_1 = CH_3, R_2 = H$

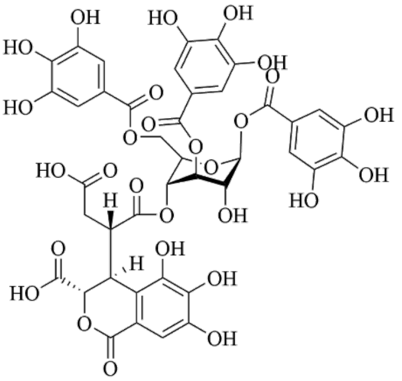

76

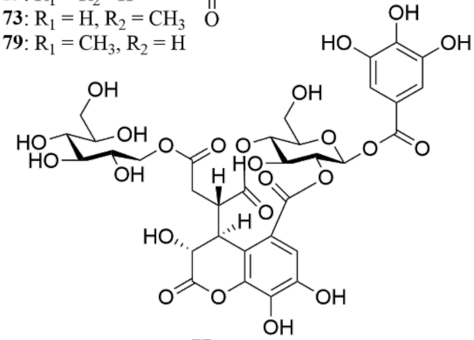

77

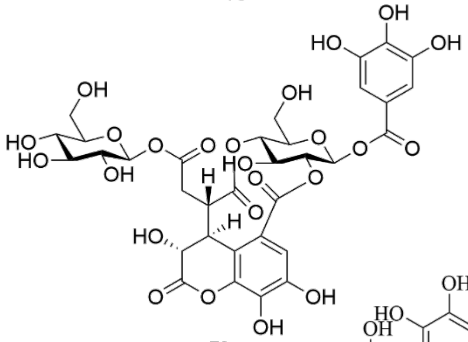

78

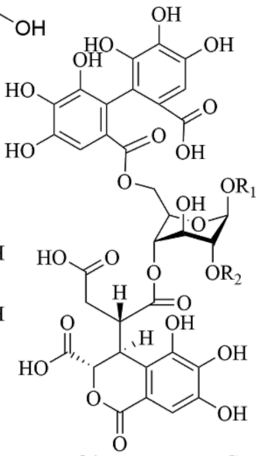

84: $R_1 = H, R_2 = G$
91: $R_1 = G, R_2 = H$

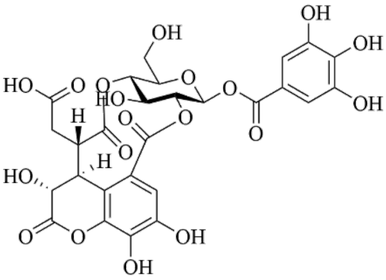

80

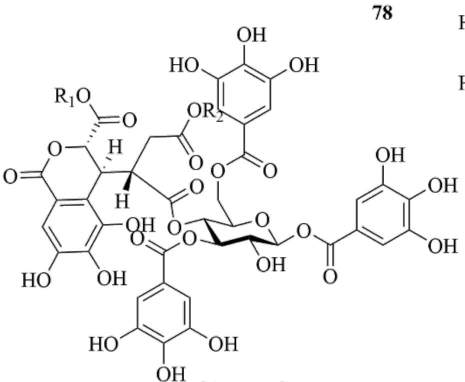

81: $R_1 = CH_3, R_2 = H$
98: $R_1 = H, R_2 = CH_3$

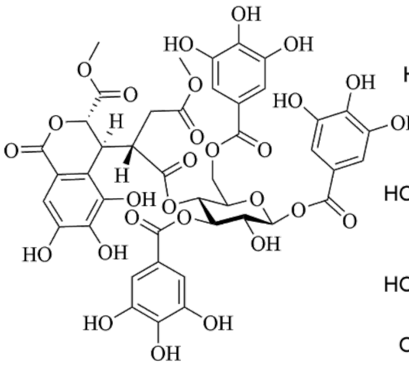

85

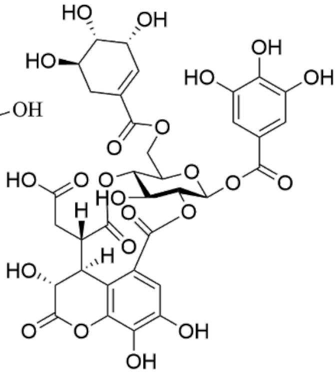

88

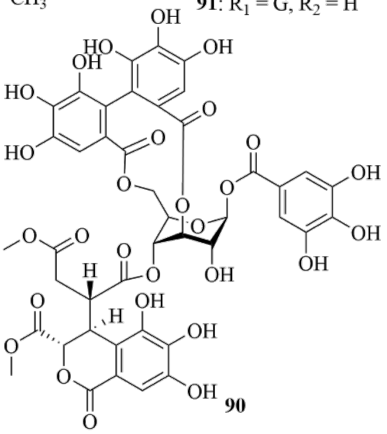

90

Fig. S34 Chemical structures of the identified 122 compounds (Continued)

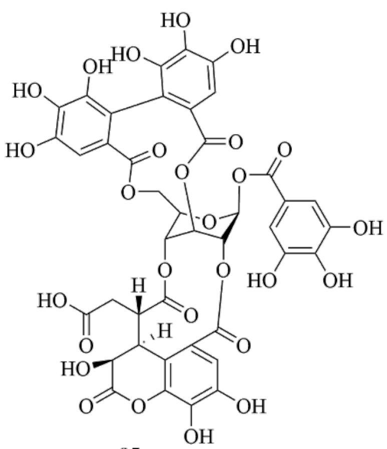

95

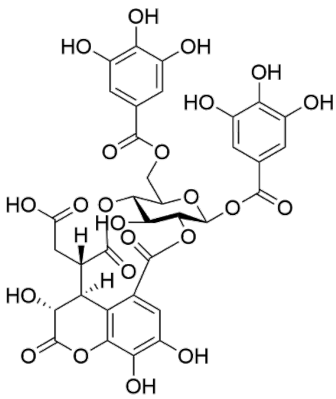

96

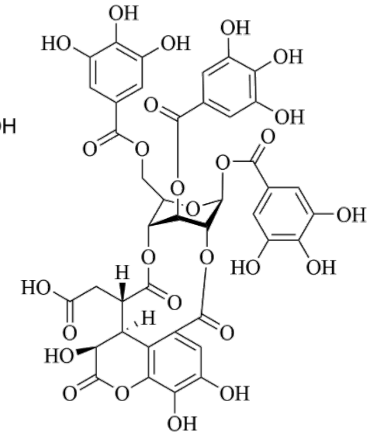

107

flavonoids

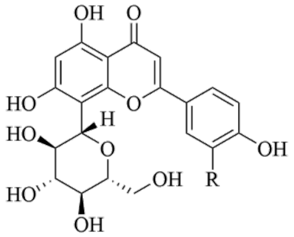

87: R = OH
93: R = H

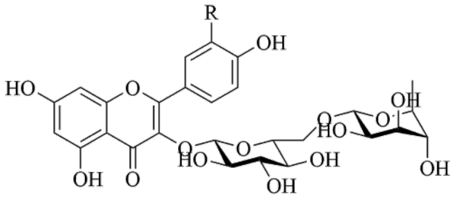

99: R = OH
104: R = H

triterpenoids

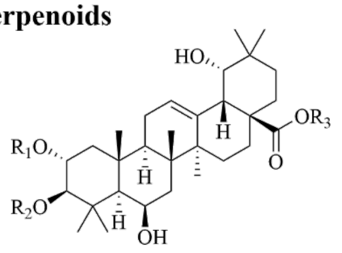

102: R_2 = Glc, R_1 = R_3 = H
103: R_1 = Glc, R_2 = R_3 = H
109: R_3 = Glc, R_1 = R_2 = H

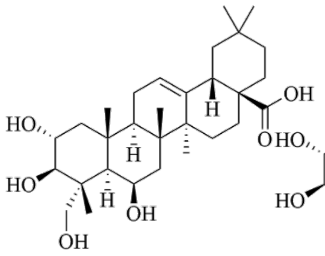

113

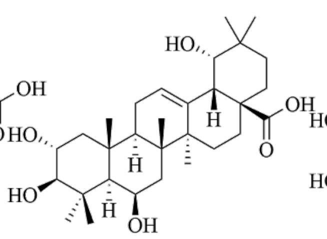

114

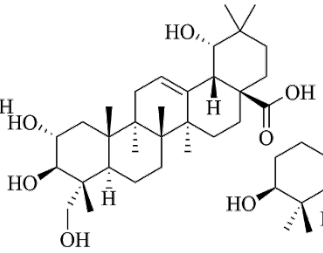

115

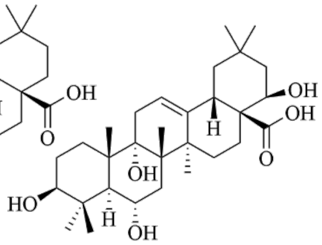

117

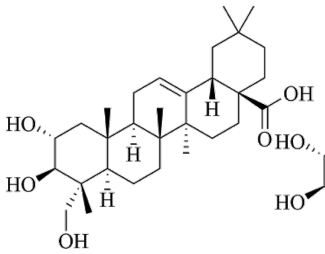

119

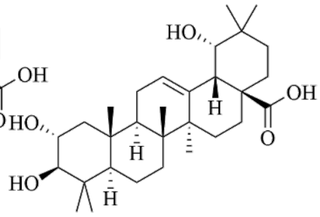

120

Fig. S34 Chemical structures of the identified 122 compounds (Continued)

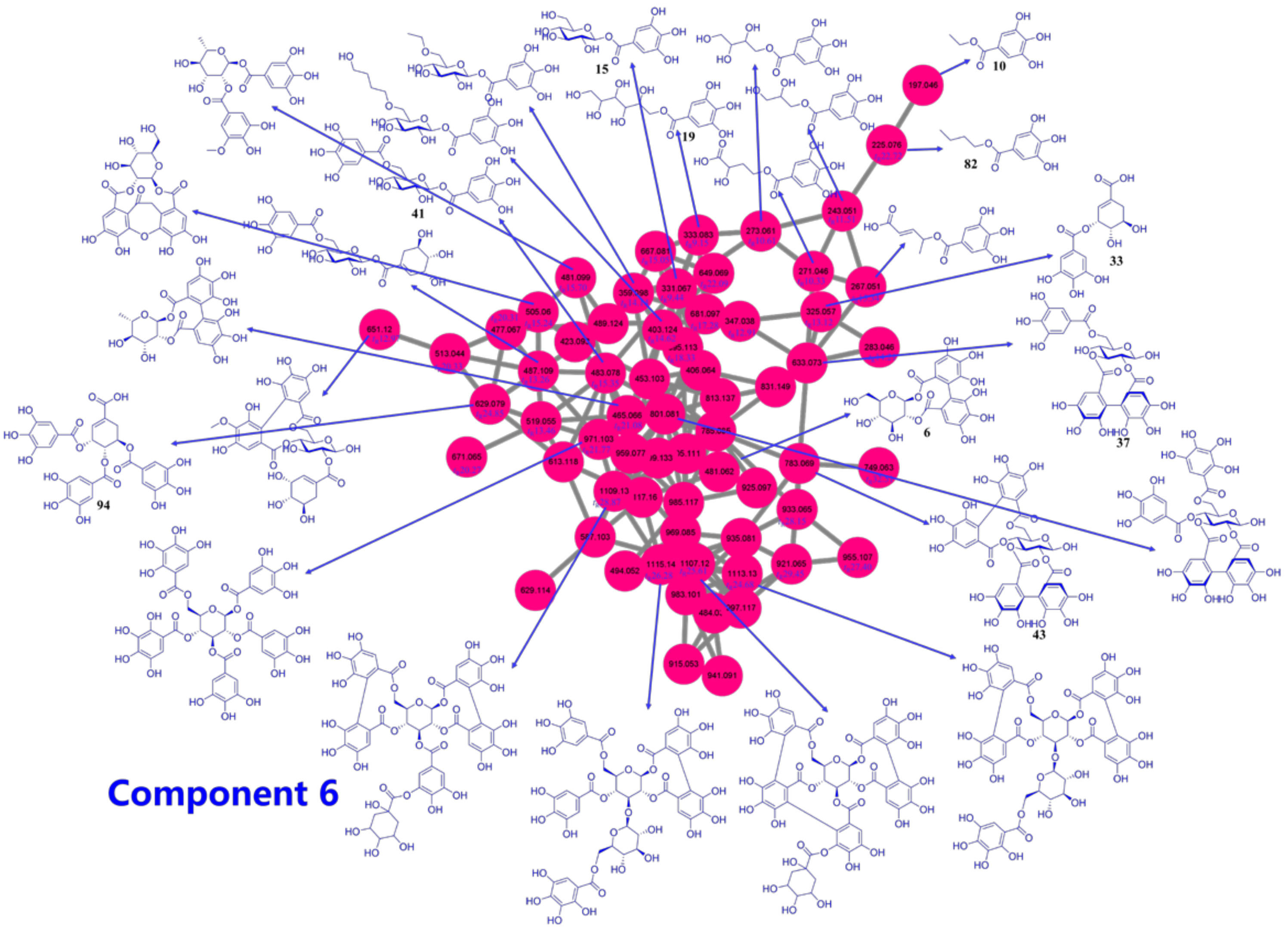

Fig. S35 Annotation on nodes of precursor ion in component **6**.

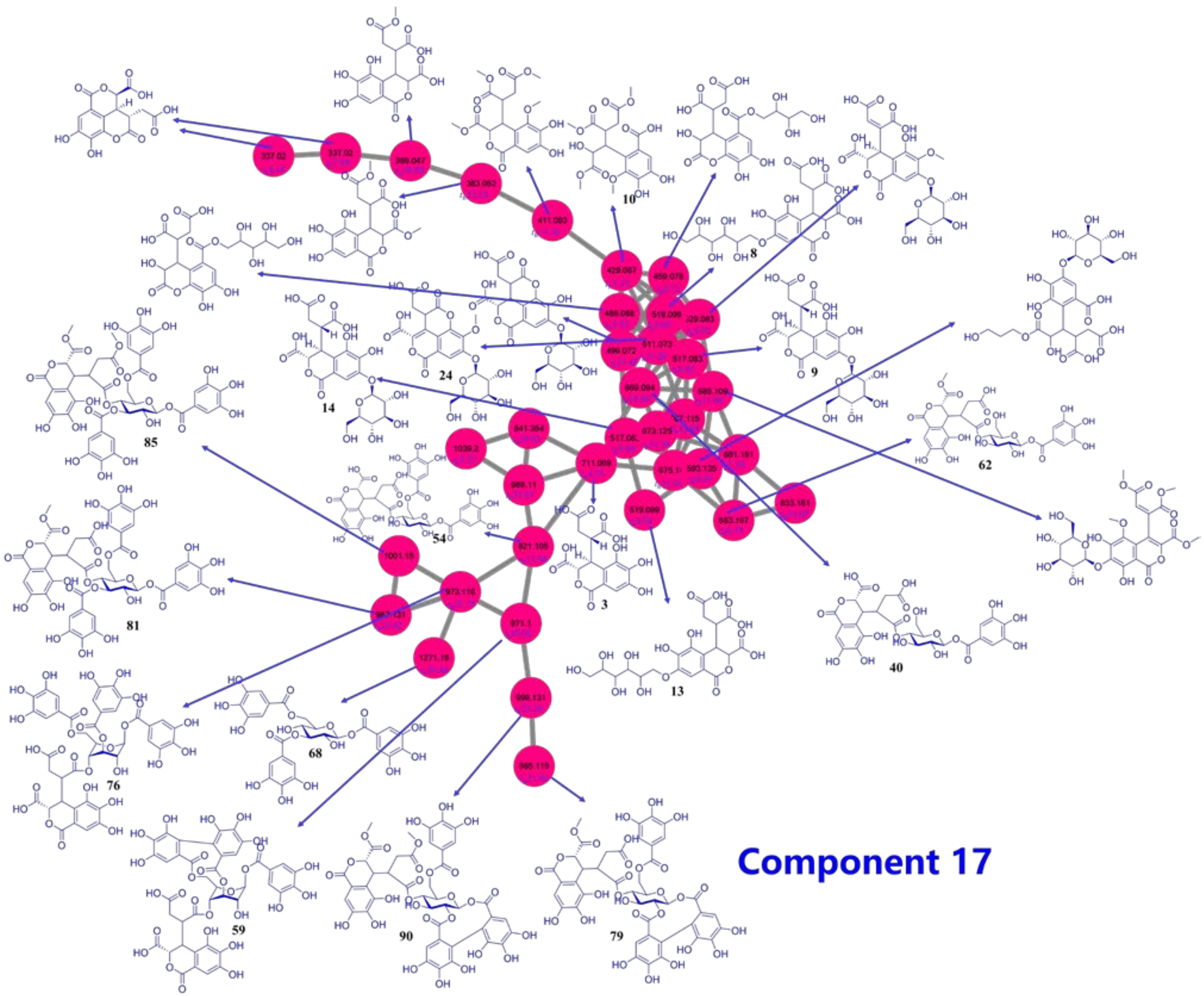

Fig. S36 Annotation on nodes of precursor ion in component **17**.

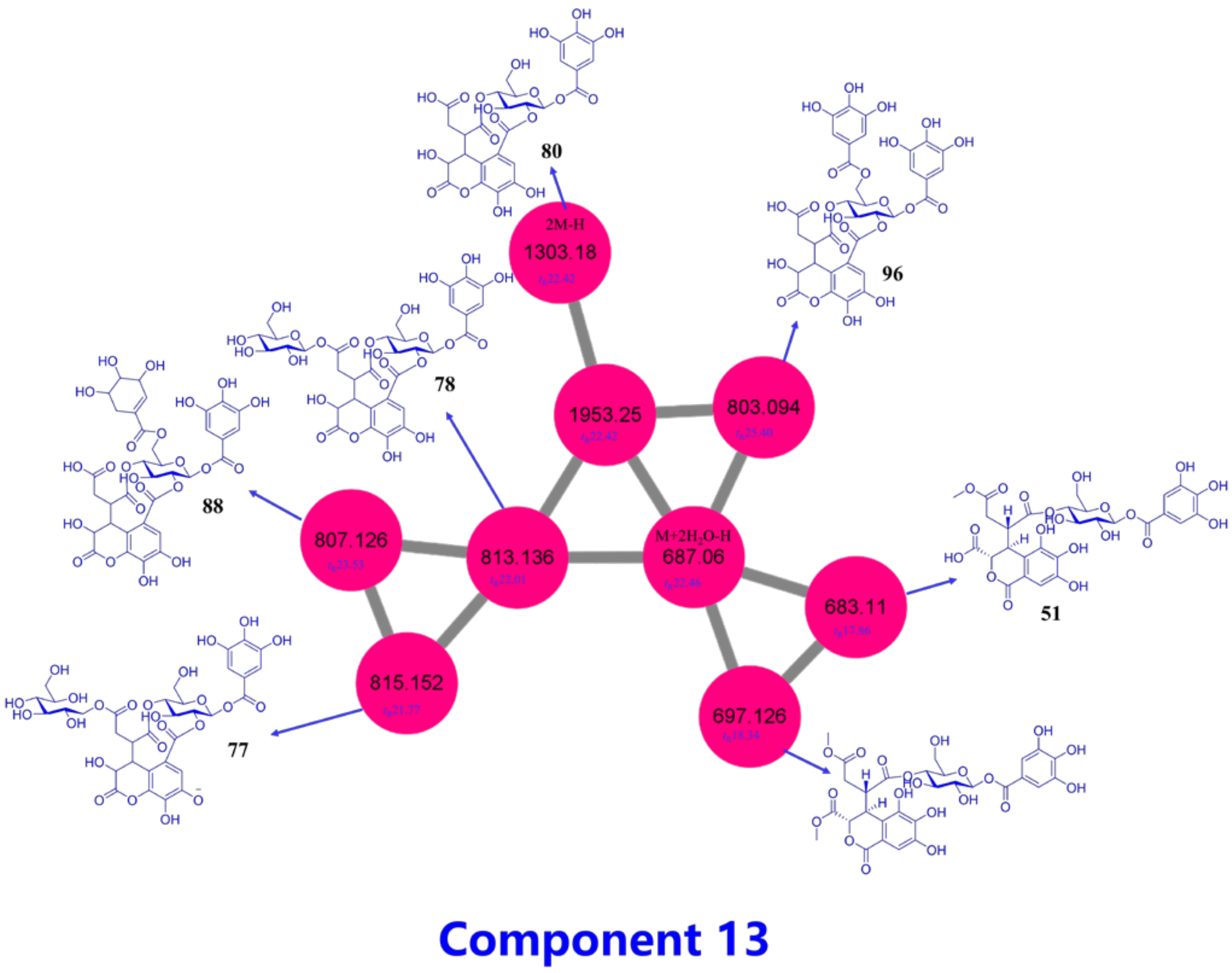

Fig. S37 Annotation on nodes of precursor ion in component **13**.

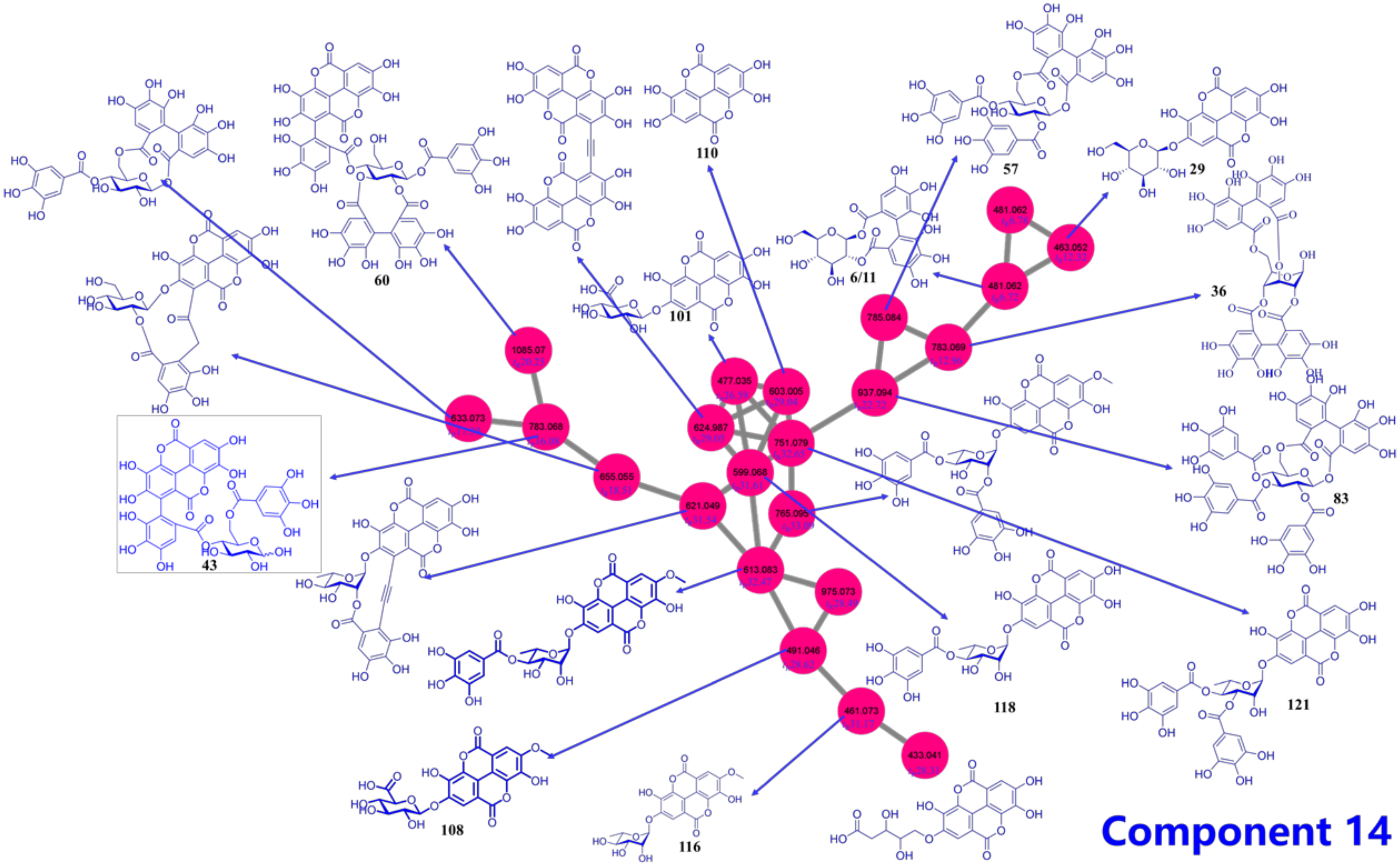

Fig. S38 Annotation on nodes of precursor ion in component **14**.

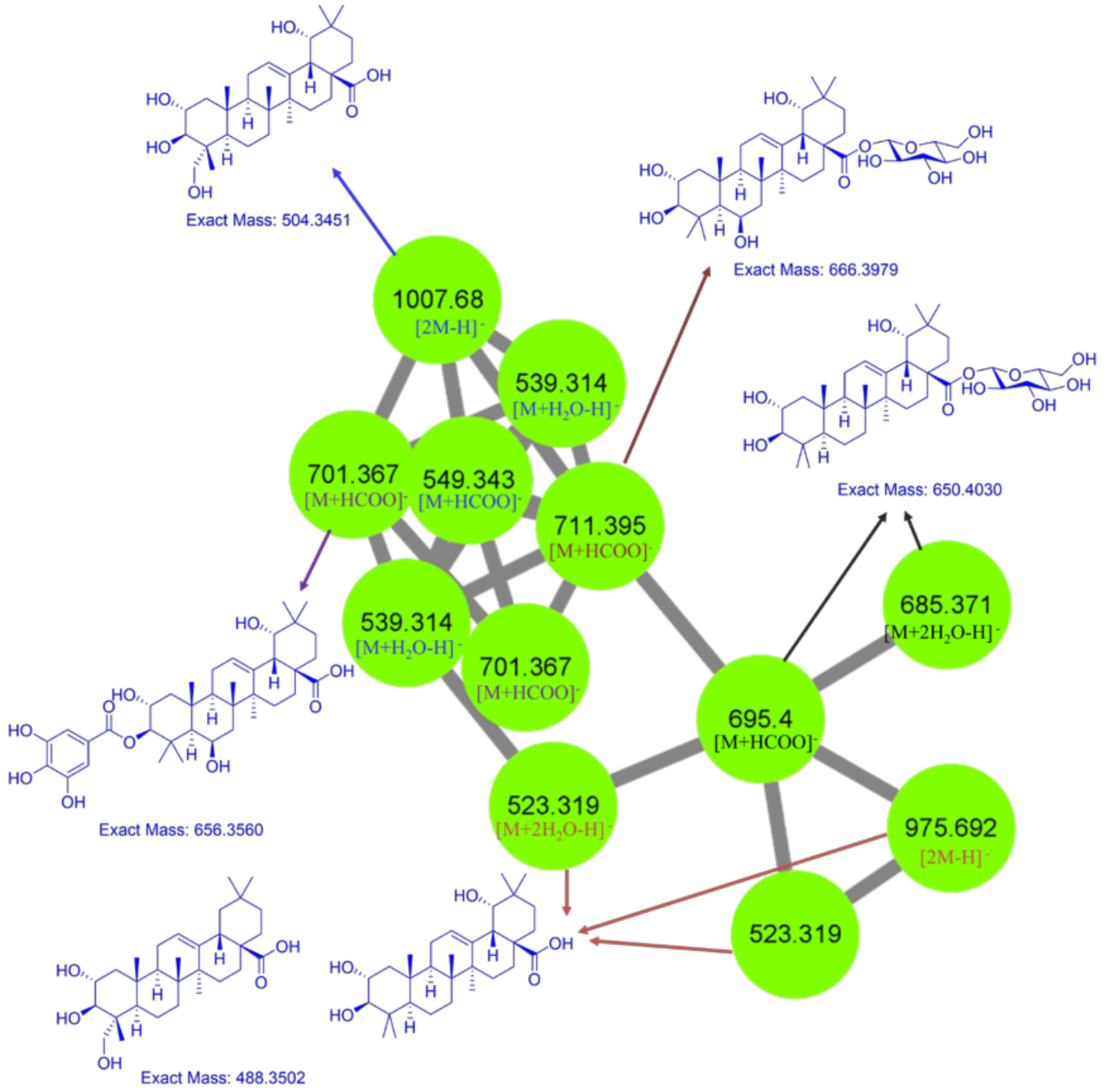

Fig. S39 Annotation on nodes of precursor ion in component **4**.

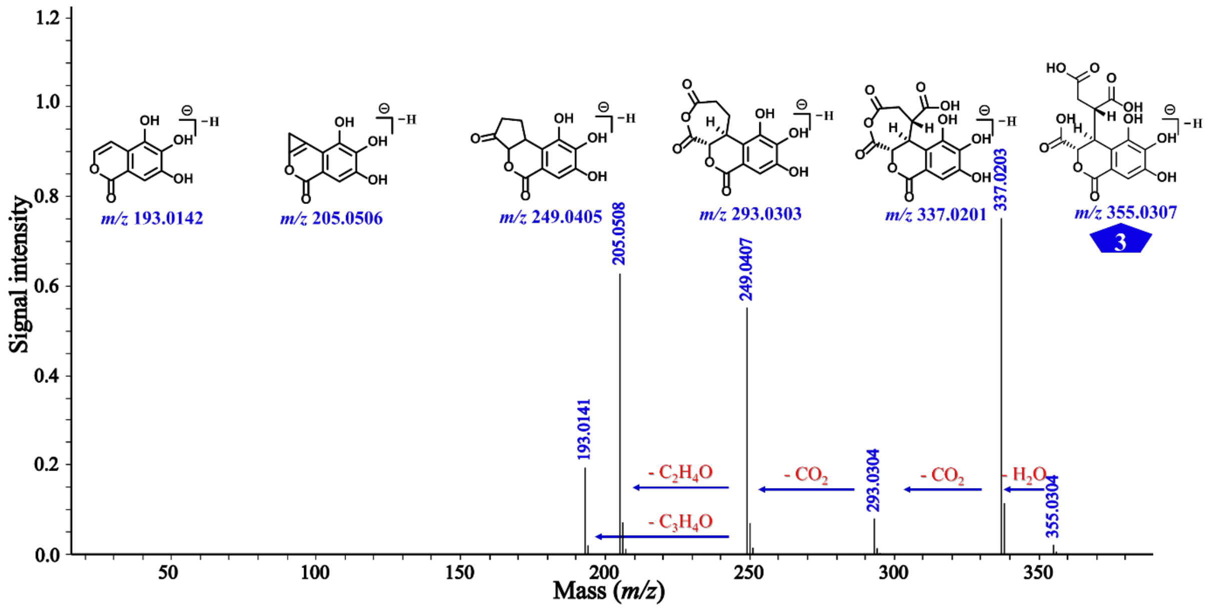

Fig. S40 The cleavage law of **chebulic acid** in mass spectrometry

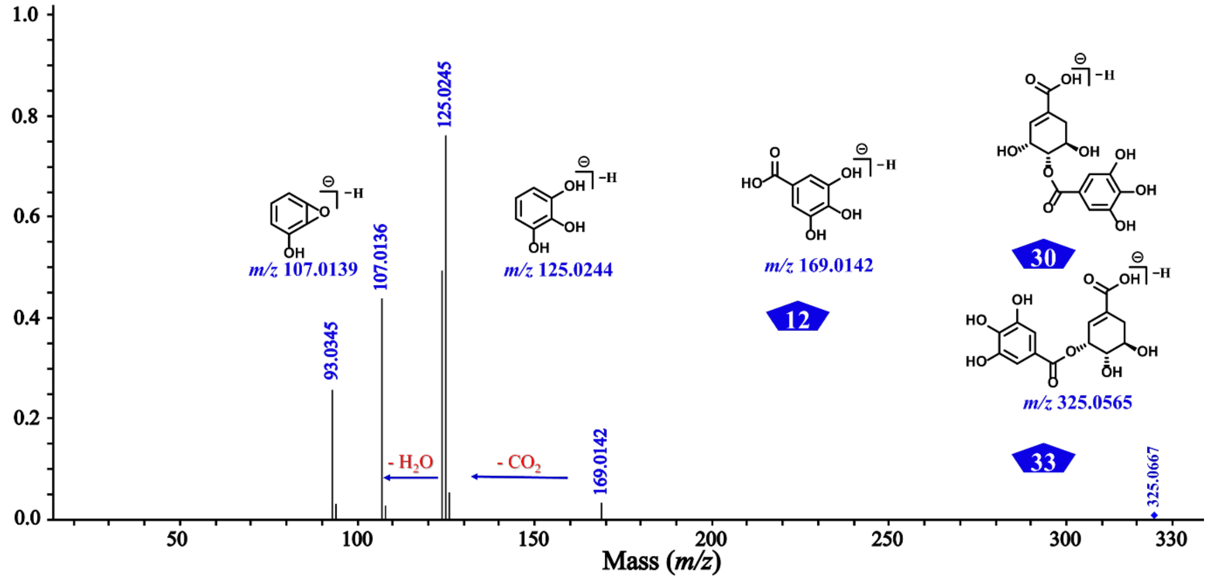

Fig. S41 The cleavage law of **gallic acid**, **4-*O*-galloyl-(-)-shikimic acid**, and **5-*O*-galloyl-(-)-shikimic acid** in mass spectrometry

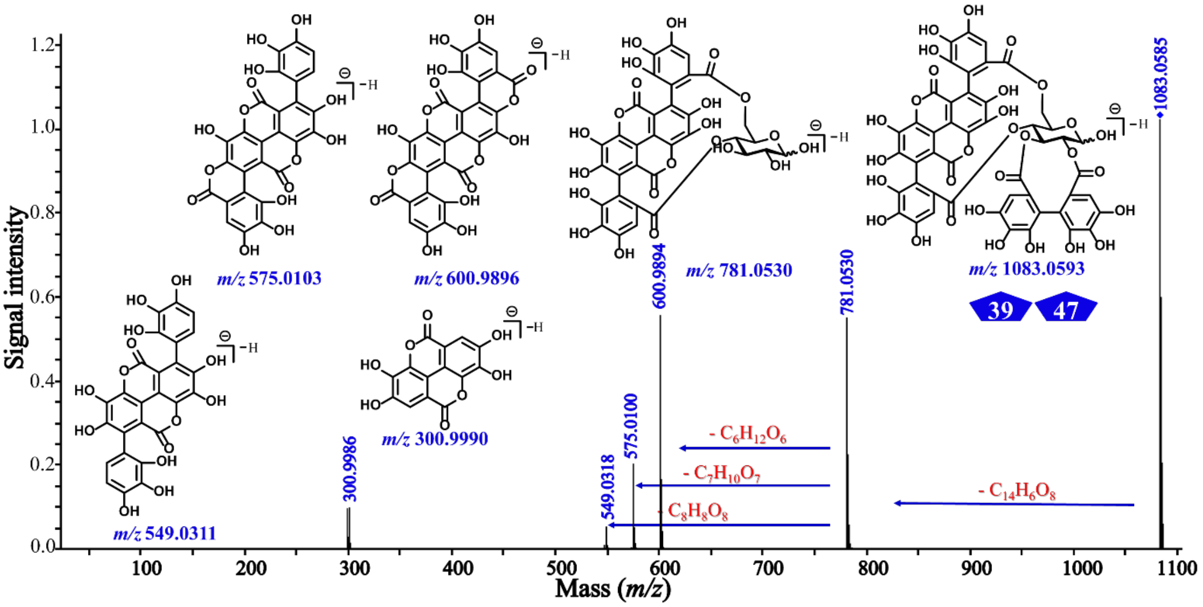

Fig. S42 The cleavage law of **punicalagins A & B** in mass spectrometry

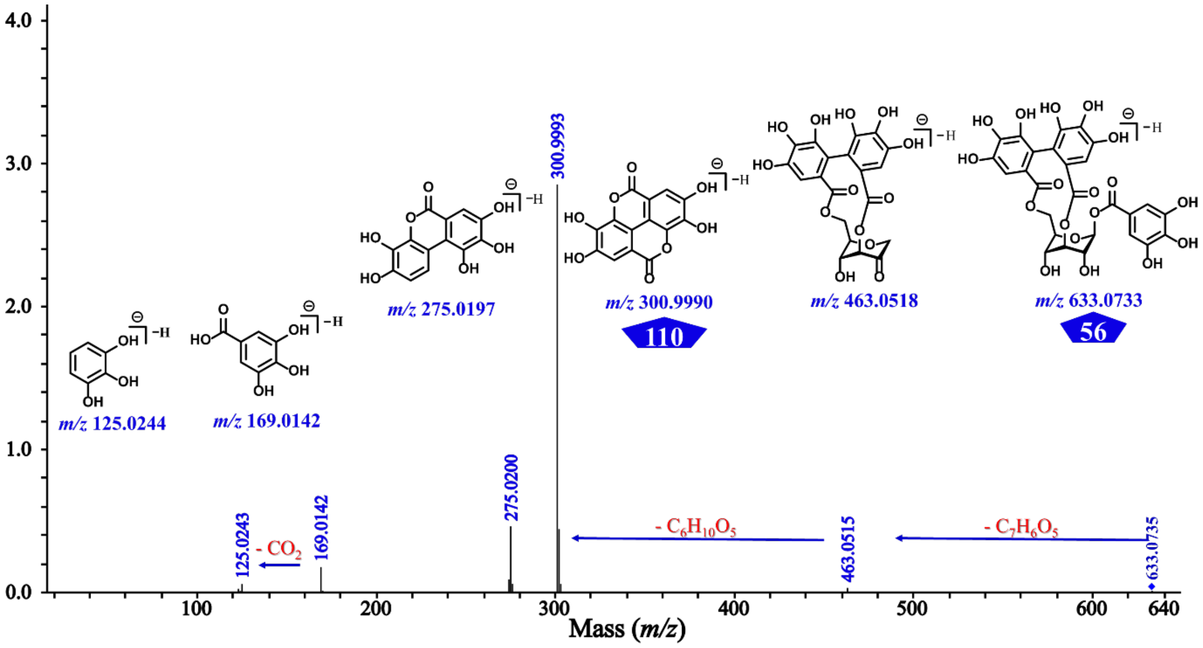

Fig. S43 The cleavage law of **corilagin** and **ellagic acid** in mass spectrometry

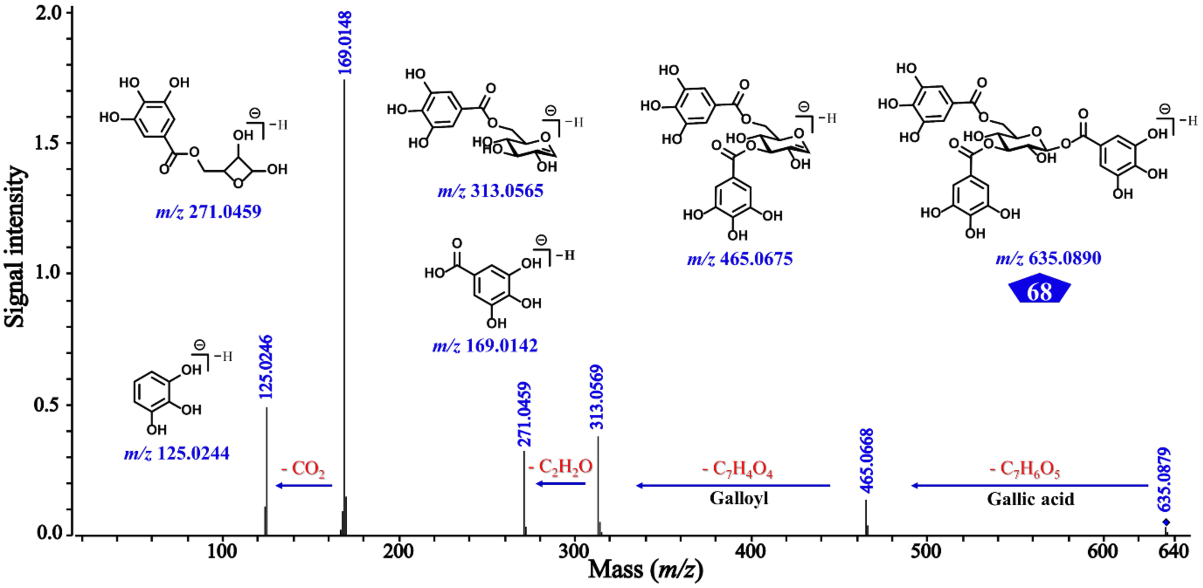

Fig. S44 The cleavage law of **1,3,6-tri-*O*-galloyl-*β*-D-glucose** in mass spectrometry

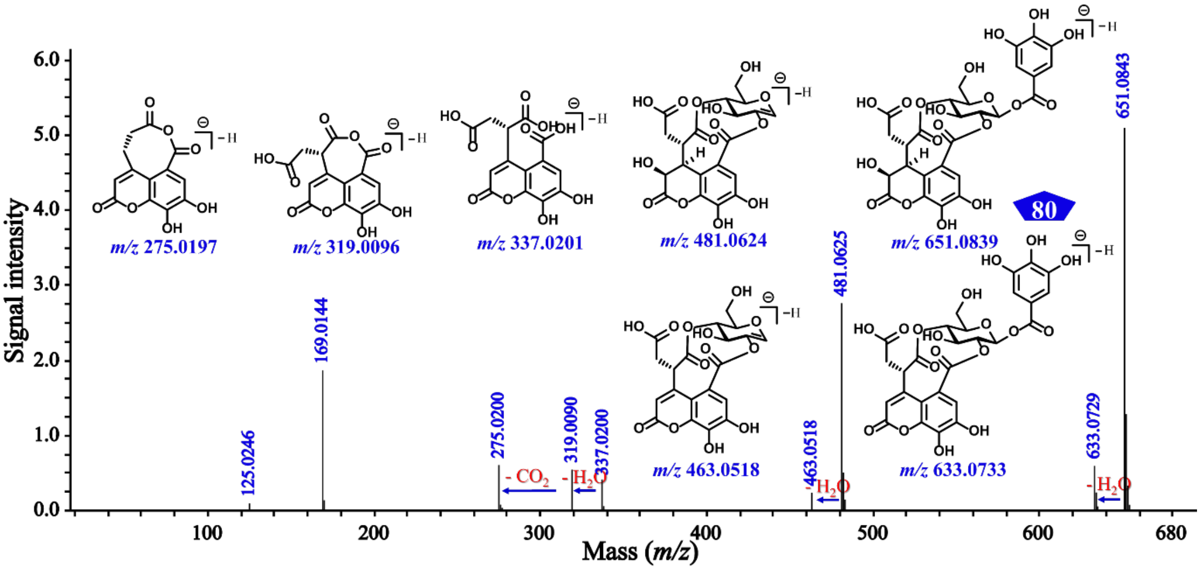

Fig. S45 The cleavage law of **chebulanin** in mass spectrometry

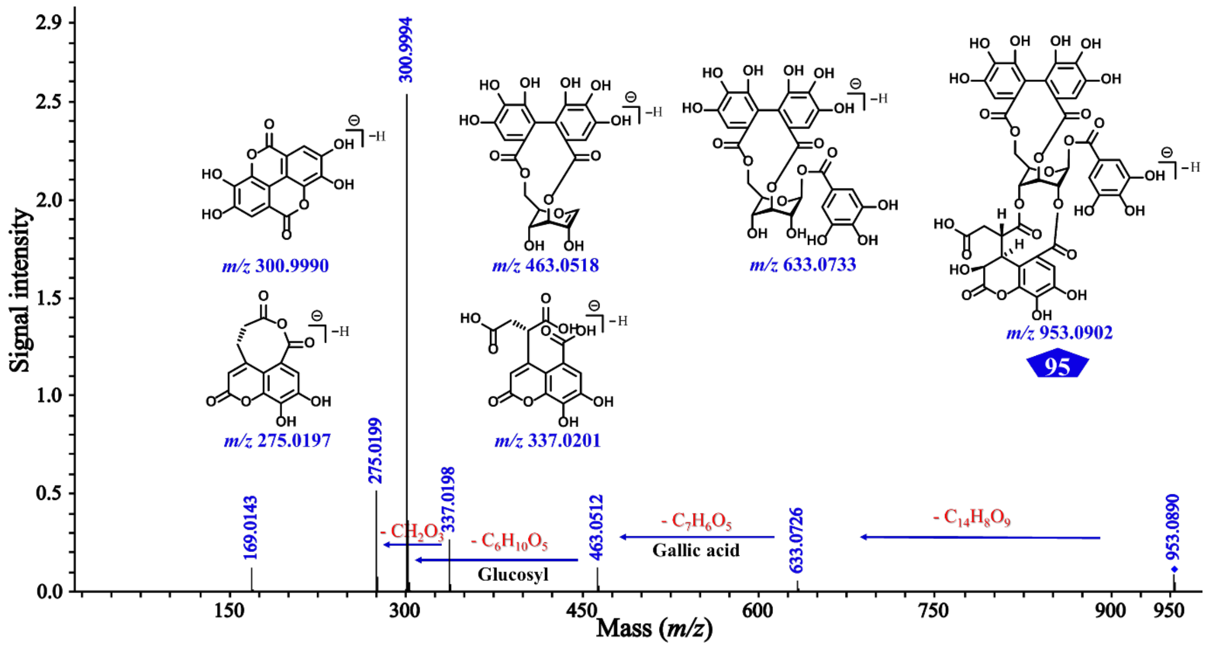

Fig. S46 The cleavage law of **chebulagic acid** in mass spectrometry

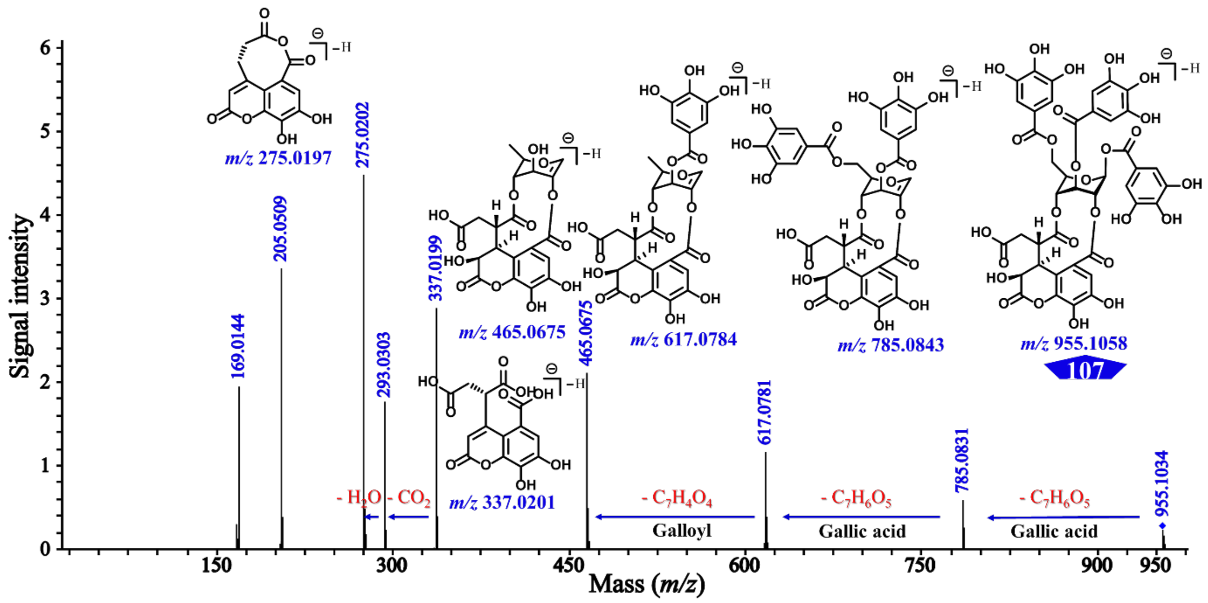

Fig. S47 The cleavage law of **chebulinic acid** in mass spectrometry

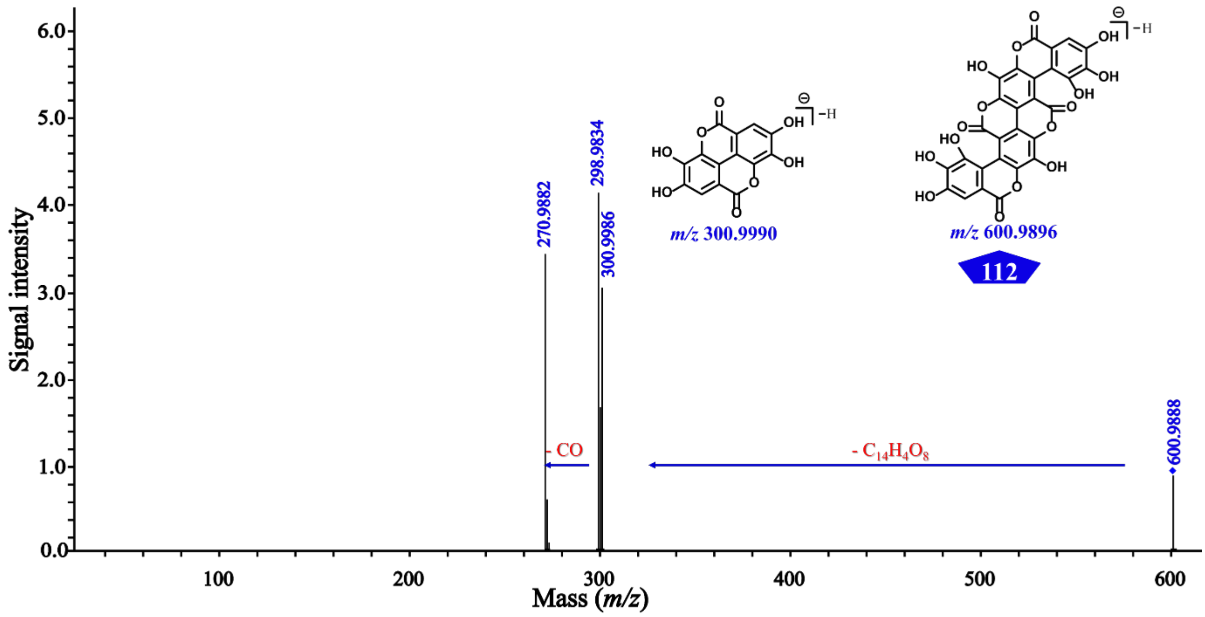

Fig. S48 The cleavage law of **terminalin** in mass spectrometry

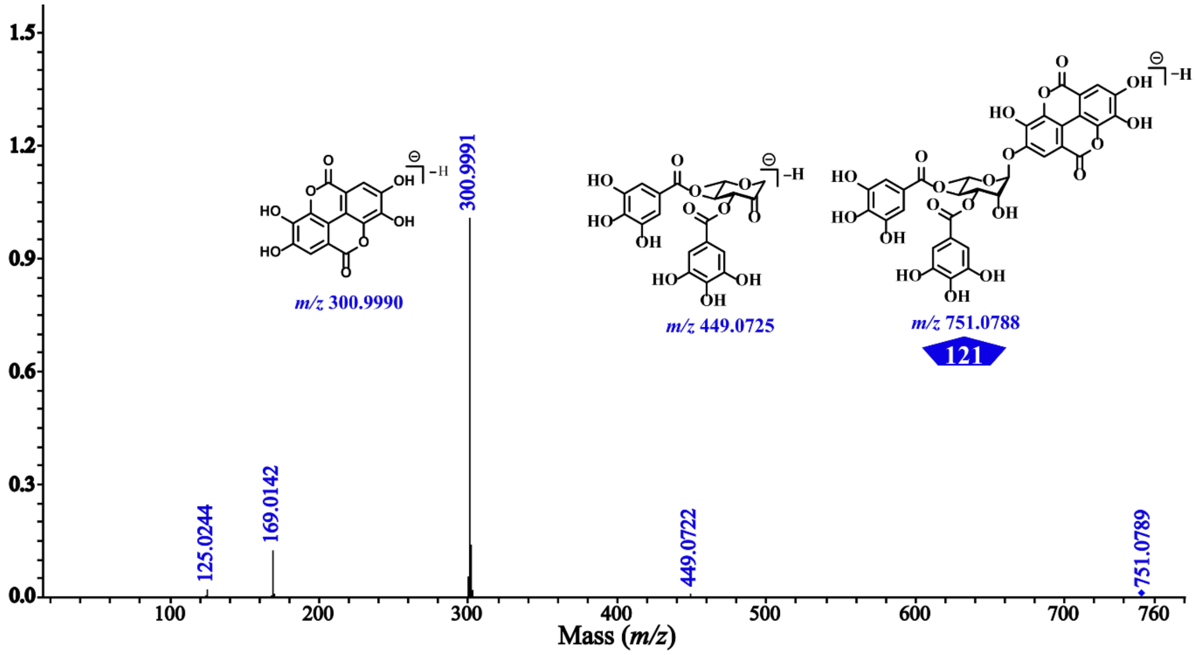

Fig. S49 The cleavage law of **4-*O*-(3'',4''-di-*O*-galloyl-*α*-L-rhamnosyl)ellagic acid** in mass spectrometry

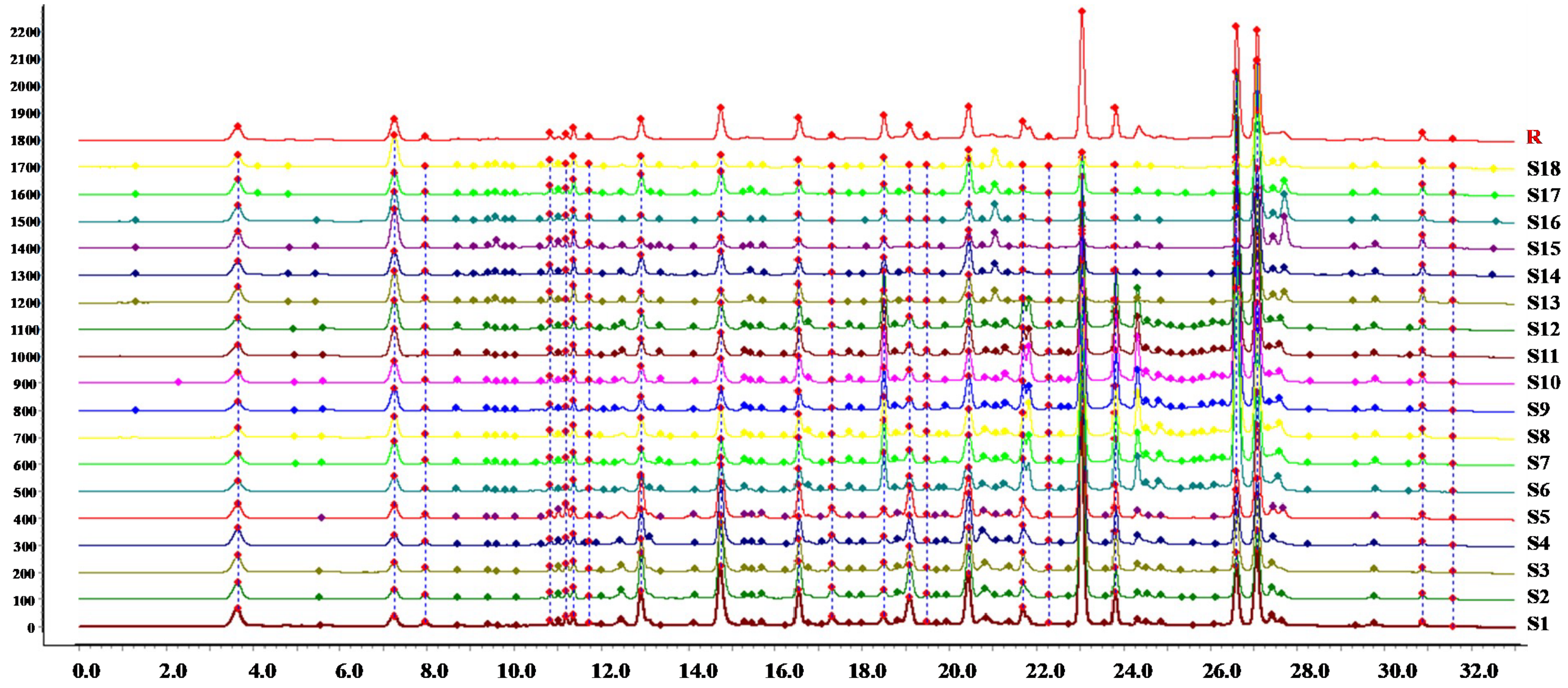

Fig. S50 UPLC fingerprints of 18 batches of CF (Figure 4A).

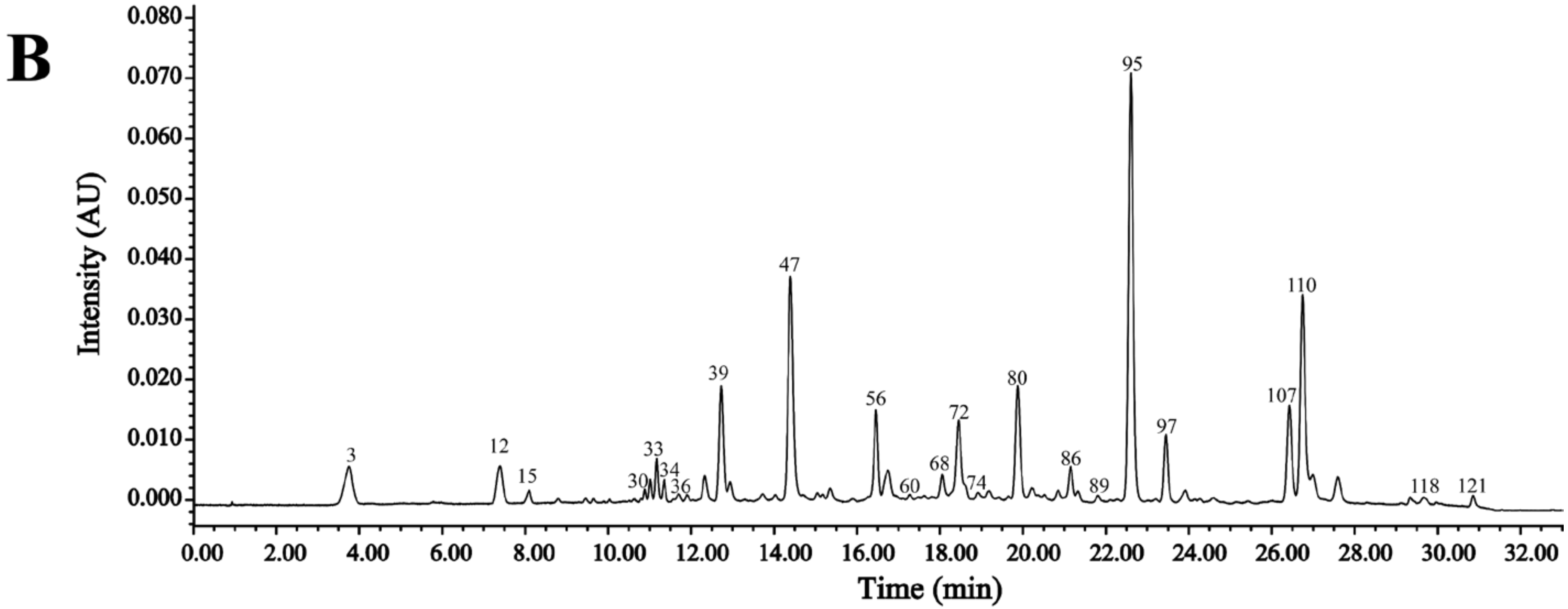

Fig. S51 The representative UPLC fingerprints marked with 23 common peaks (Figure 4B).

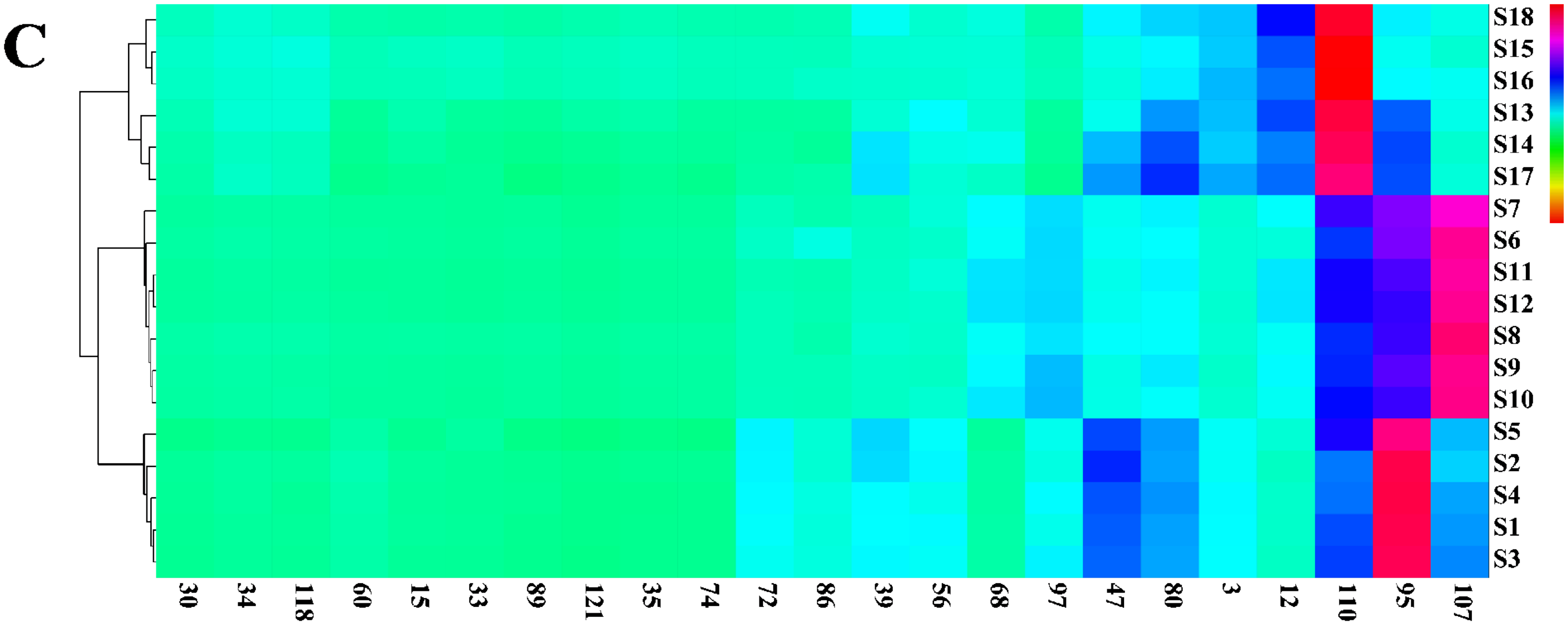

Fig. S52 The heat-map of 18 batches of CF and the twenty-three constituents (Figure 4C).

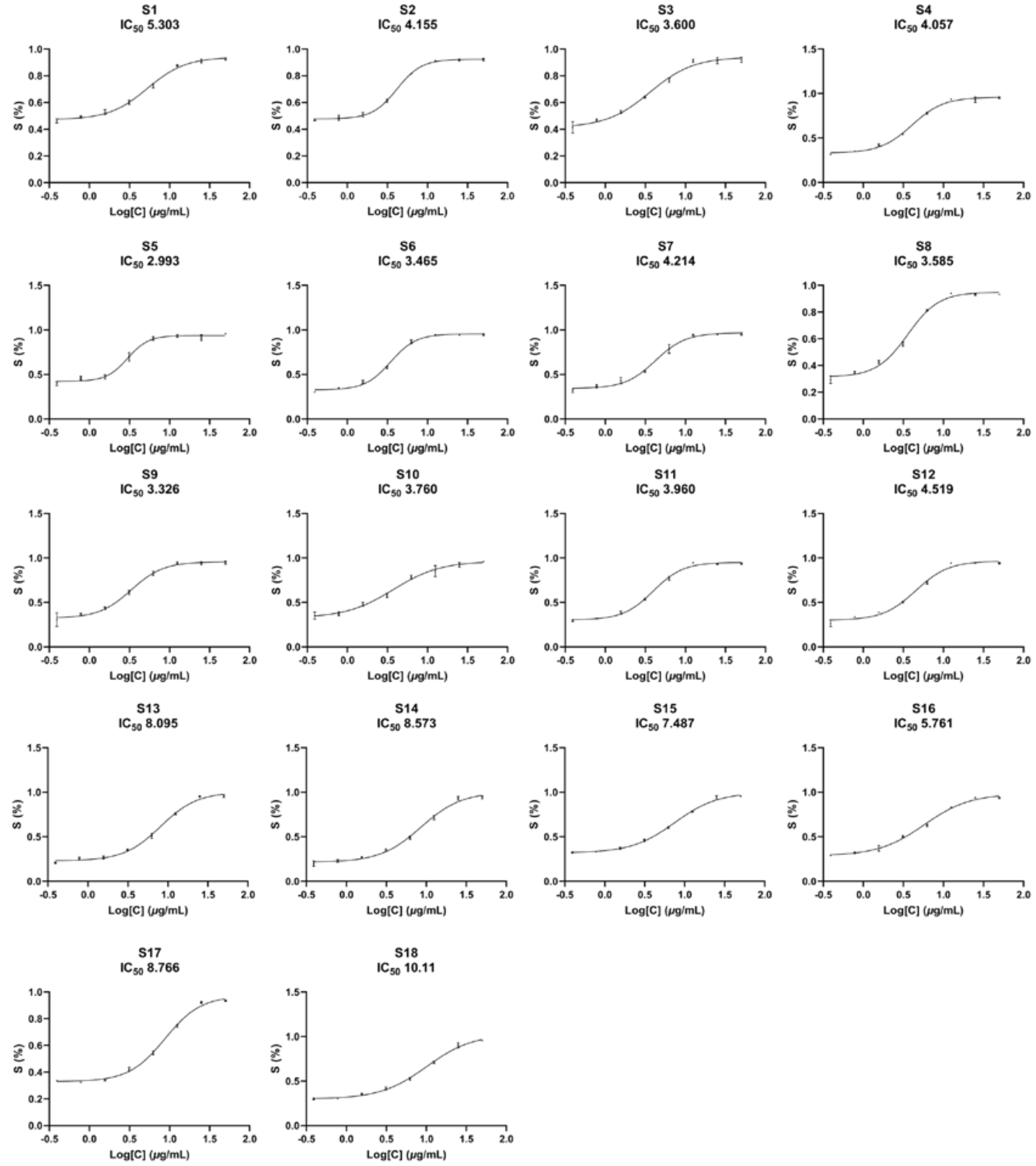

Fig. S53 $DPPH^{\bullet}$ radical scavenging activities of 18 batches of the methanol extracts from CF. The values were expressed by IC_{50} (μg/mL, n = 3).
